# Studying the long-term adaptation of *Haloferax volcanii* to low salt conditions: transcriptomic and genetic analyses

**DOI:** 10.3389/fmicb.2025.1697018

**Published:** 2026-01-15

**Authors:** Andreas Borst, Jörg Soppa

**Affiliations:** Institute for Molecular Biosciences, Goethe University, Frankfurt, Germany

**Keywords:** archaea, complementation, deletion mutants, *Haloferax volcanii*, low salt adaption, RNA-Seq, stress adaptation, transcriptomics

## Abstract

*Haloferax volcanii* is a moderately halophilic archaeon with an optimal salt concentration of 2.1 M NaCl. It is an archaeal model species used for the characterization of various biological processes. It can grow in media with widely varying salt concentrations, but the mechanisms for the adaptation to the extremes of low and high salt are unknown. To get an overview of the adaptation to low salt, the transcriptomes of cultures grown at 0.9 M NaCl were compared to that of cultures grown at the optimal salt concentration of 2.1 M NaCl. Nearly 20% of the transcriptome was found to be differentially regulated. Twelve genes or gene cluster that were induced at low salt were chosen and *in frame* deletion mutants were generated. Phenotypic analyses revealed that the absence of four proteins led to a growth defect at low salt or totally inhibited growth. Homologous overproduction of three proteins revealed that high levels of HVO_1863 might be lethal, while the production of HVO_B0276 and HVO_0772 in the cognate deletion mutant rescued the phenotype. Taken together, the lower NaCl concentration limit for growth has been established, an overview of differentially regulated genes is presented, and the relative importance of 12 genes has been characterized. For the first time proteins were identified that are very important or essential for growth at low salt, while they are dispensible for growth at the optimal salt concentration.

## Introduction

Hypersaline environments can be found all around the world at places of high temperatures and intense solar radiation, resulting in an evaporation rate that exceeds the inflow of water. This includes lakes of every size, man-made solar salterns used for salt production, and the Dead Sea. Microorganisms living in these habitats have to cope with the high osmotic pressure, and two different strategies have evolved. Halophilic bacteria typically use the so-called “salt-out strategy” (Sleator and Hill, 2002; Harding et al., 2016; Gunde-Cimerman et al., 2018). The intracellular salt concentration is kept low, and the osmotic balance is brought about by the accumulation of compatible solutes, small organic, often zwitter-ionic molecules, e.g., betaine, amino acids, or glycerol-derivatives. In contrast, halophilic archaea typically use the so-called “salt-in strategy” (Gunde-Cimerman et al., 2018). The high external salt concentration is balanced by an equally high internal salt concentration. When the cells are well energized, KCl is accumulated, generating a KCl gradient (high internally) and a NaCl gradient (high externally). When the cells are later devoid of any energy source, a sodium-proton antiporter can dissipate the NaCl gradient and thereby establish a proton gradient, enabling the production of ATP under starvation conditions. The salt-in strategy is less energy-demanding than the salt-out strategy, however, all cellular processes must be adapted to function in the presence of molar concentrations of salt. This has led to very acidic proteomes (pI 4–5) in haloarchaea, because proteins need a high charge density on their surface to remain stable and soluble under these conditions. Hence usage of the salt-in strategy can be identified by bioinformatic analysis of genome sequences. Typically, haloarchaeal proteins do not only function in the presence of high salt, they also require high salt for their stability and denature under low salt conditions (although exceptions exist). Consequently, not only the proteins, but also haloarchaeal cells need a rather high minimal salt concentration in their environment to survive. Extreme halophilic as well as moderate halophilic haloarchaea exist, which have different optimal and minimal salt concentrations for growth.

Historically, the Dead Sea has been thought to be devoid of life due the extreme conditions, hence its name. However, about 80 years ago Elazari Volcani and others discovered microbial life in the Dead Sea and began to isolate and characterize endogenous species (Wilkansky, 1936; Elazari-Volcani, 1940, 1943, 1944). Since then halophilic archaea, halophilic bacteria, and halophilic algae have been repeatedly isolated from the Dead Sea. Exactly 50 years ago Mullakhanbai and Larsen isolated a halophilic archaeon that they named *Halobacterium volcanii* to honor the pioneering work of Elazari volcani (Mullakhanbhai and Larsen, 1975), which was later renamed to *Haloferax volcanii* (Oren, 2006).

For decades *H. volcanii* was believed to have a pleiomorphic cell shape, be non-motile, require at least 1.7 M salt to grow, and to have an aerobic metabolism. Today we know that none of these early assumptions is entirely true. For example, *H. volcanii* is motile and chemotaxis-positive under microaerobic conditions (Tripepi et al., 2010), it can grow anaerobically via nitrate or DMSO respiration (Wanner and Soppa, 1999), and at very early exponential phase it is rod-shaped (Silva et al., 2021). Several years ago we have established growth of *H. volcanii* in microtiter plates and used it for the comprehensive characterization of several features (Jantzer et al., 2011). Surprisingly, we found that *H. volcanii* can grow at salt concentrations as low as 0.7 M, half the concentration of 1.4 M that was believed to be the lower limit until then (Jantzer et al., 2011). Later, we were unable to obtain growth at 0.7 M salt in Erlenmeyer flasks. We realized that we had failed to take the salt concentration of the inoculum into account. For growth in microtiter plates, 20 μL inoculum was mixed with 130 μL of medium. As the inoculum had been in basal salts containing 2.2 M NaCl, the concentration of the medium of 0.7 M NaCl was increased to 0.9 M in the 150 μL that were used to monitor growth. Therefore, the salt concentration had been in fact slightly higher than reported by us (Jantzer et al., 2011), but still much lower than what had been reported before.

Microorganisms are continuously exposed to rapid changes in their environment, e.g., changes in oxygen availability, concentrations of carbon, nitrogen, phosphorus, etc., light intensity, and many more physical, chemical, and biological environmental parameters. Typically, transcription factor networks have evolved, which mediate differential gene expression to adapt the transcriptome to the momentary conditions. For a rapid adaptation of the transcriptome not only the rapid production of new transcripts is needed, but also the rapid removal of transcripts that are not needed any more. Therefore, typically the half-life of transcripts is much shorter in prokaryotes than in higher eukaryotes, e.g., 2–3 min in *Escherichia coli* or 5–7 min in *Halobacterium salinarum* (Hundt et al., 2007). In addition, various post-transcriptional and post-translational regulatory mechanisms exist to adapt the transcriptome and the proteome as needed. These include small non-coding regulatory RNAs (sRNAs) as well as differential acetylation, methylation, and phosphorylation of proteins (Alam et al., 1989; Smith et al., 1997; Soppa, 2010; Schlesner et al., 2012; Babski et al., 2014; Gelsinger and DiRuggiero, 2018; Couto-Rodríguez et al., 2023).

As the knowledge about the real lower salt concentration for growth was established only rather recently, the mechanisms of adaptation of *H. volcanii* to this low salt conditions is totally unknown. Several studies of osmoadaptation of haloarchaea and of *H. volcanii* exist, but typically the focus was on high-salt adaptation, and, when low salt adaptation was included, the lowest limit was 1.4–1.8 M NaCl. Furthermore, the methods used in earlier studies allowed only the identification of single differentially expressed genes or proteins (Ferrer et al., 1996; Mojica et al., 1997; Bidle, 2003). Several publications reported on the transcriptome, on the proteome or on genome-wide glycosylation of proteins at low salt (Guan et al., 2012; Heyer et al., 2012; Jevtić et al., 2019). However, these studies defined 1.7 M NaCl to 1.8 M NaCl as “low salt,” concentrations that are much higher than the actual lowest salt concentration *H. volcanii* can grow in.

Therefore, we decided to perform a comprehensive comparison of the transcriptomes of cultures grown at 0.9 M NaCl versus cultures grown at the optimal concentration of 2.1 M NaCl. Low salt induced transcripts were identified, and their possible importance was investigated by the generation of *in frame* deletion mutants of the encoding genes. Two mutants with interesting phenotypes were chosen, and their phenotypes were complemented via the homologous production of the respective proteins.

## Results

### Growth under low salt and experimental setup for transcriptome analyses

At first, growth of *H. volcanii* was characterized in synthetic glucose medium with 0.7 M NaCl, 0.9 M NaCl, and the optimal salt concentration of 2.1 M NaCl. There are two ways of varying the salt concentration. Some authors vary the concentrations of all salt in the medium simultaneously (often denoted as x-fold sea salts), while others vary only the concentration of the major salt, NaCl. We chose the latter approach, and the concentrations of all other salts were kept constant (220 mM MgCl_2_, 41 mM MgSO_4_, 133 mM KCl, and 9 mM CaCl). A culture volume of 45 mL was chosen to be able to perform the subsequent RNA-Seq analysis using identical culture conditions.

**FIGURE 1:**
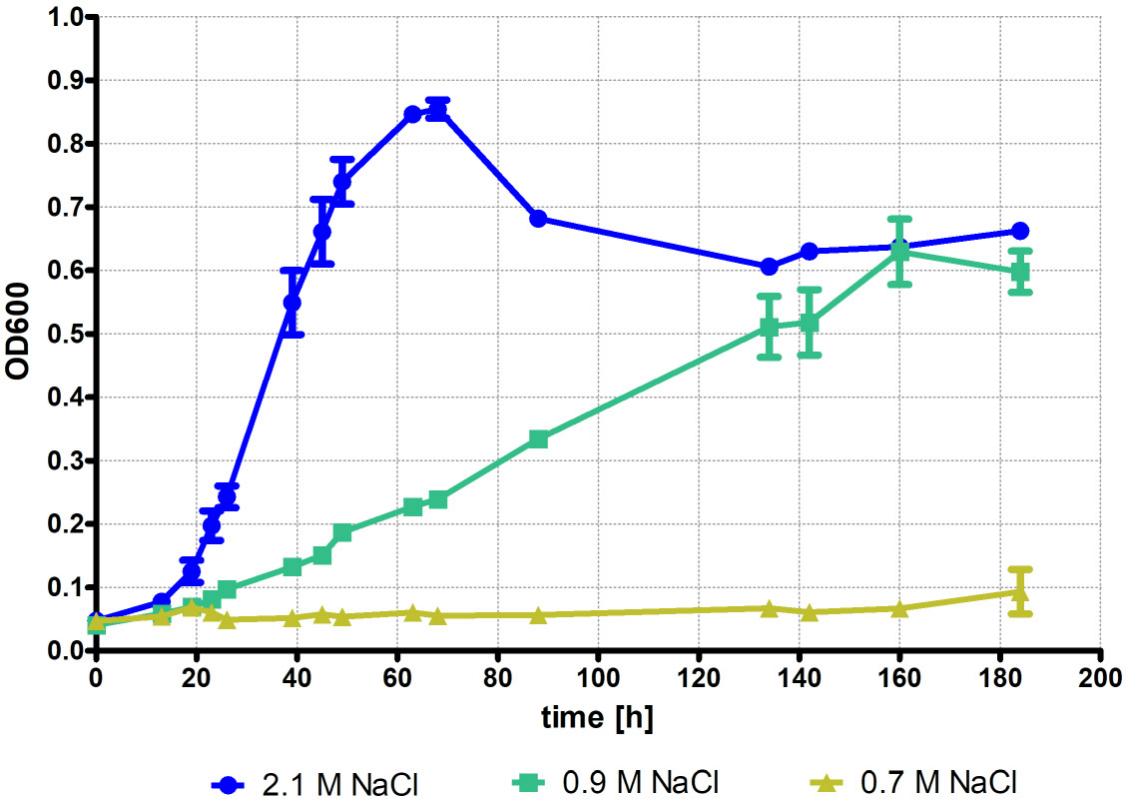
*Haloferax volcanii* H26 wild-type cells were grown in synthetic glucose medium with different NaCl concentrations. A preculture was grown in 2.1 M NaCl, cells were collected, resuspended in basal salts and used to inoculate the test cultures with an OD_600_ of 0.05. Cultures were grown in 45 ml medium in Erlenmeyer flasks with the indicated NaCl concentrations. Growth of three biological replicates was measured frequently over several days. Average values and standard deviations are shown.

The results are shown in Figure 1. It could be verified that *H. volcanii* does not grow at all in 0.7 M salt, and no adaptation to this concentration occurred even after more than 180 h of incubation. In stark contrast, growth in 0.9 M NaCl began after a short lag phase, and steady state growth was observed until 160 h, when stationary phase was reached. The lag time prior to the onset of growth in 2.1 M NaCl was virtually identical, but then growth was much faster, and the stationary phase was reached after 60 h, about 100 h earlier than in the 0.9 M culture. However, growth yields for both cultures were identical. For both conditions growth was very reproducible, and the variances of the three biological replicates were marginal. These results prompted us to compare the transcriptomes of cultures grown in 0.9 M NaCl vs. 2.1 M NaCl.

**FIGURE 2:**
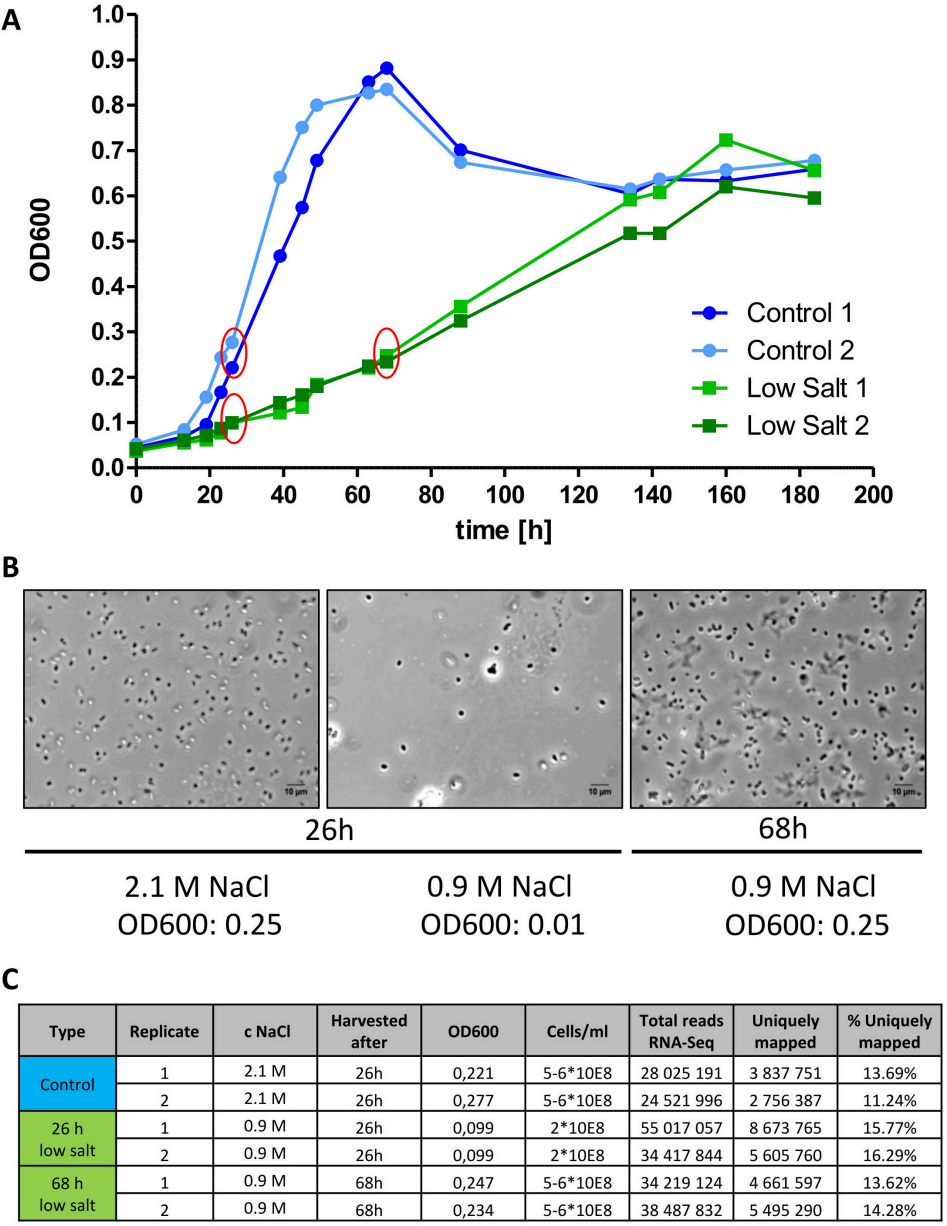
**(A)** Two replicates of H26 were grown in synthetic glucose medium with 2.1 M NaCl (blue) or 0.9 M NaCl (green) in Erlenmeyer flasks (start OD_600_ of 0.05). Red circles indicate cultures and time points at which samples were removed for RNA-Seq analysis. **(B)** Microscopic pictures of H26 taken at indicated NaCl concentrations, time points and OD_600_ values. Contrast enhancement was used to optimize the visualization of cells. The size bars indicate 10 μm. **(C)** Characteristics of the 6 samples and the respective total and uniquely mapped reads from the RNA-Seq analysis.

The experimental design for the RNA-Seq experiment is shown in Figure 2A. We decided to use two biological replicates for the analysis. Again, the growth curves could be reproduced. Samples were removed from the 2.1 M culture after 26 h, during early to mid-exponential growth phase. Samples from the 0.9 M cultures were removed after 26 h, so the incubation time was identical, but the OD_600_ was much lower. In addition, samples were also removed after 68 h, at an identical OD_600_ as for the culture grown in 2.1 M NaCl. This should enable to perform three different transcriptome comparisons to study long-term adaptions of *H. volcanii* to low salt conditions: (1) 0.9 M versus 2.1 M after the identical time, (2) 0.9 M vrsus 2.1 M at the identical OD_600_, and (3) the 0.9 M culture at two different time points, which should clarify whether further adaptation occurred after prolonged growth. Figure 2B shows microscopic images of the cells from the three sample types, revealing that cell size and cell shape were identical.

### RNA isolation and RNA-Seq analyses

RNA was isolated from the six samples using a phenol/chloroform method that was also used for previous RNA-Seq analyses, and residual DNA was removed by DNase digestion (compare Methods). RNA Seq was performed by the company StarSEQ (Mainz, Germany).^1^ The results were sent to us as fastq files, and they have been deposited in the Gene Expression Omnibus (GEO) under the accession number GSE278471.

**FIGURE 3:**
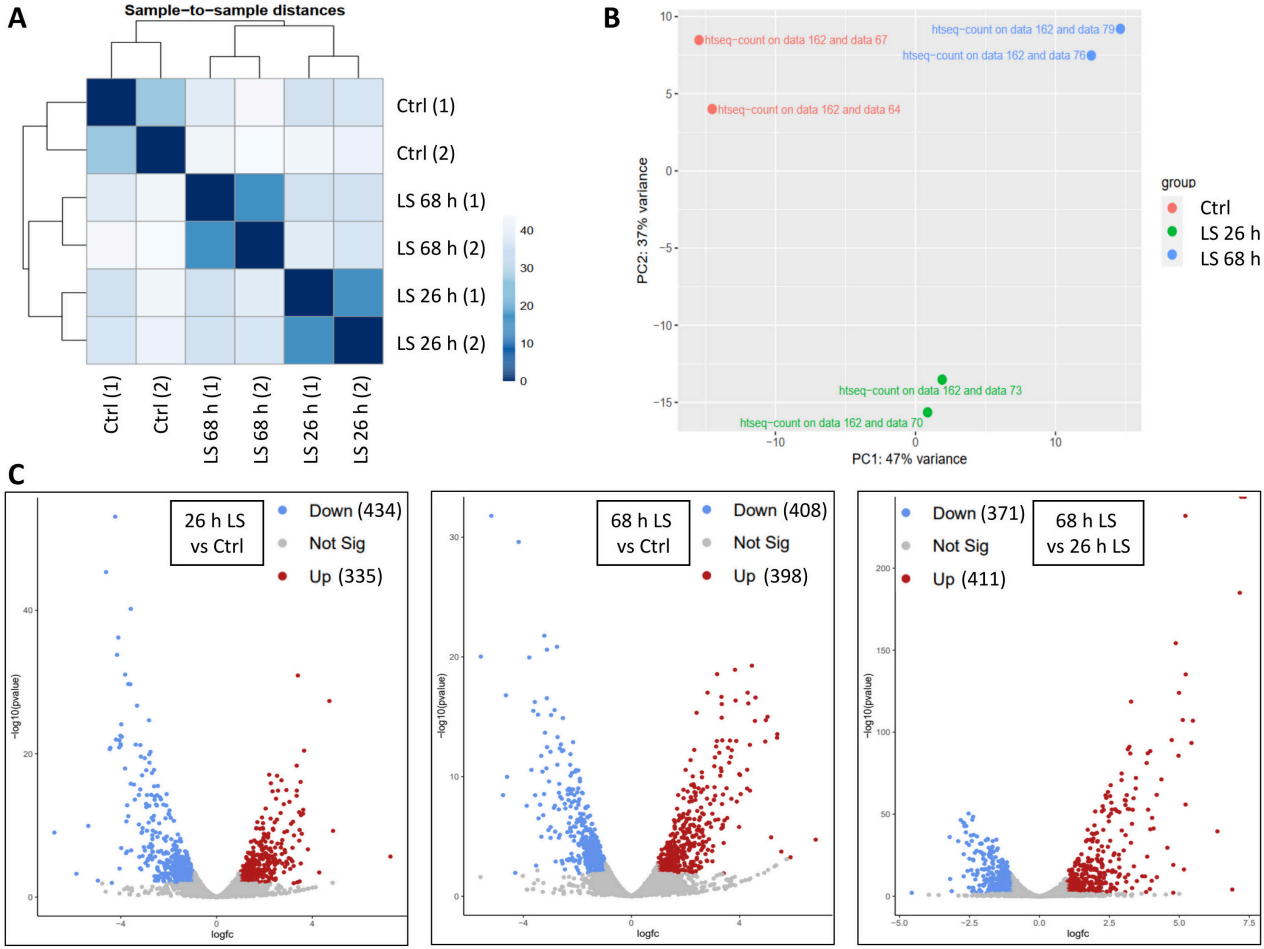
Overview of RNA-Seq quality and results: sample to sample distances **(A)** and Principal component analysis **(B)** as calculated by the DESeq2 tool (2.11.40.8) via the galaxy platform. Volcano plots **(C)** visualize the regulation levels of all genes and the statistical significance of the results. The applied threshholds are indicated (adjusted *p* < 0.05 and at least a two-fold up- or downregulation) for each of the three transcriptomic comparisons. The number of up- and downregulated genes are indicated in brackets. In this plot “fc” denotes “fold change.”

First, quality checks were performed. A sample to sample distance analysis verified the high reproducibility of the results for the biological duplicates (Figure 3A). Similarly, a principle component analysis revealed that the biological replicates of the three conditions grouped together, respectively, and were distant from each other (Figure 3B).

Next, the reads were mapped to the genome sequence of *H volcanii*. A version was used that was curated by Friedhelm Pfeiffer (Martinsried, Germany, obtained at September 13th, 2023) and thus contained an annotation that was up to date. Unfortunately, it turned out that the rRNA removal had not been very efficient. On average 30 million reads had been obtained for each of the six samples, only 3–8 million of which could be uniquely mapped to the genome and were devoid of rRNA sequences (Figure 2C). Nevertheless, the average number of 4 million uniquely mapped reads were enough to represent the transcriptomes and allow quantitative comparisons of transcript abundances, revealing salt-dependent differential regulation of gene expression.

### Transcriptome differences between cultures growing under optimal versus low salt conditions

For the analysis of differential regulation, three comparisons were performed, i.e., both of the low salt samples were compared to the control and the two low salt samples were compared to one another. Only transcripts were analyzed that were detected in both biological replicates. Two volcano plots depicting all transcripts are shown in Figure 3C, the applied threshholds of twofold up- or down-regulation and *P* = 0.05 are indicated. On average, roughly 400 transcripts were upregulated, and 400 transcripts were downregulated in low salt. The difference between the two low-salt samples was in a similar range, indicating that the adaptation to the low salt condition was not completed after 26 h, but a major further remodeling of the transcriptome occurred until 68 h. In total around 800 transcripts were more than twofold differentially regulated, representing nearly 20% of all genes of *H. volcani*.

Complete and detailed overviews of all differentially regulated genes can be found in Supplementary Excel File S1 for the 26 h low salt vs. control comparison in Supplementary Excel File S2 for the 68 h low salt vs. control comparison and in Supplementary Excel File S3 for the 68 h low salt vs. 26 h low salt comparison. The Excel files can be used for searching genes of interest or for performing additional analyses.

### Enriched functional classes and different regulatory patterns

Similar to other databases, the genome database HaloLex (Pfeiffer et al., 2008) sorts the genes of *H. volcanii* into different functional classes [the access to HaloLex is restricted for safety reasons, please contact Friedhelm Pfeiffer to get access (fpf@biochem.mpg.de)]. Supplementary Excel File S4 summarizes gene and protein identifiers (HVO_numbers als well as Uniprot accession numbers), protein names, protein name categories, and functional classes (FC) for all genes of *H. volcanii* to give the readers an easy access to own follow up analyses.

It was analyzed whether some of these functional classes were overrepresented in the genes that are differentially regulated in response to low salt. Indeed, transcripts of the following functional classes exhibited a tendency to be downregulated in both low-salt samples: transcription, translation, energy, carbohydrate, and central intermediates (Supplementary Figures 1, 2). This is in excellent agreement with the fact that *H. volcanii* grows significantly slower in 0.9 M salt than in the control. In contrast, genes of the following functional classes showed a tendency of upregulation in low salt: chaperones, small molecule transport, coenzyme metabolism, replication and repair, and recombination. It is tempting to speculate that many proteins might denature under low salt conditions and need chaperons for refolding or degradation.

Our main focus was the identification of genes and gene clusters that are crucial for the ability of *Haloferax volcanii* to adapt and grow under a very low environmental salinity. Therefore, the following analyses concentrated on up-regulated genes. Follow-up studies might make use of the results to concentrate on other aspects like proteins not needed under low salt conditions or long-term adaptation (68 h vs. 26 h).

### Selection of genes for mutant analysis

**FIGURE 4:**
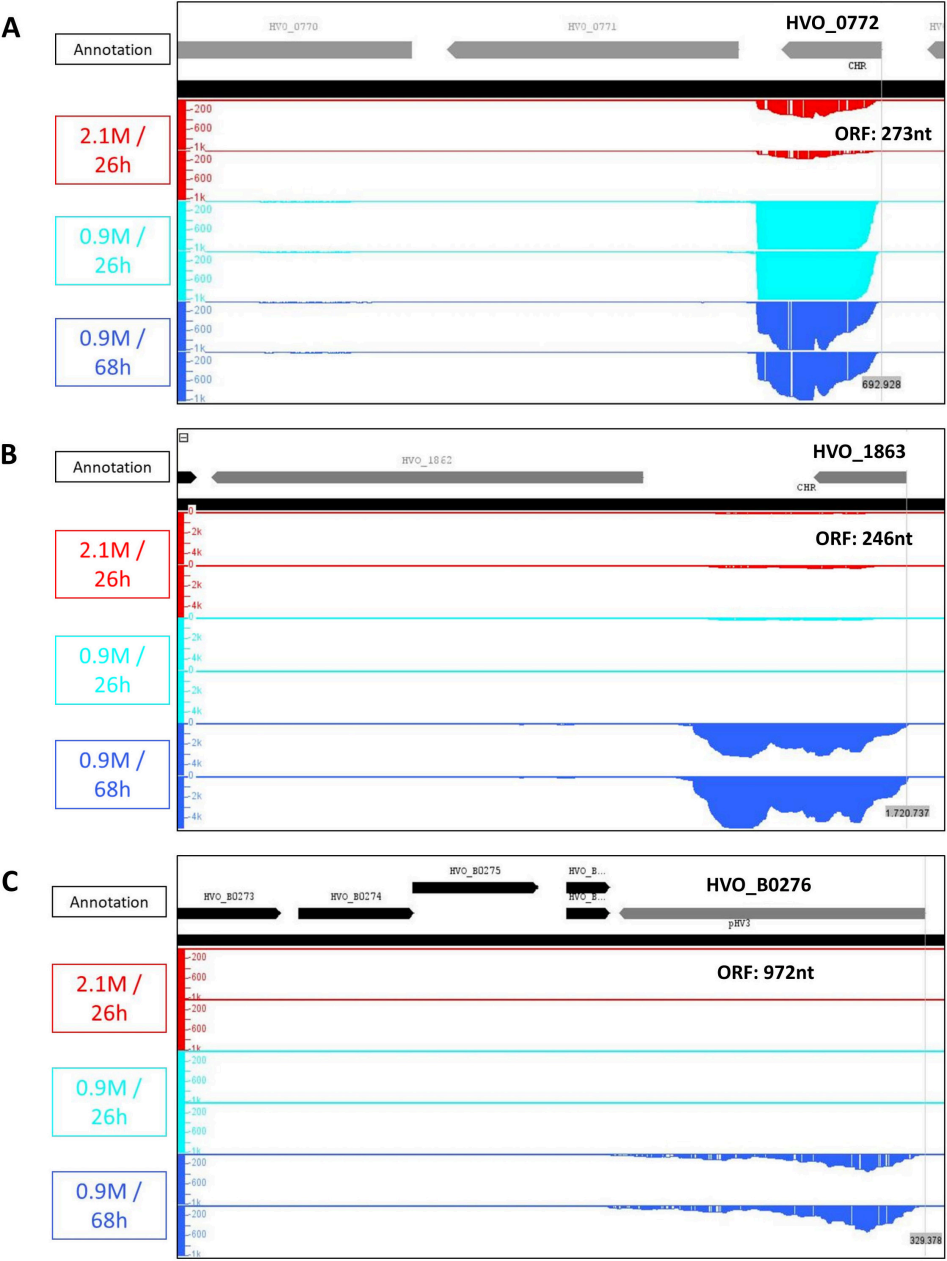
Screenshots from the Integrated Genome Browser for the following three genes to visualize peculiarities discussed in the text: **(A)** HVO_0772, **(B)** HVO_1863, and **(C)** HVO_B0276. The two replicates for the control condition are shown in red, for the 26 h low salt samples in teal, the 68 h low salt samples in blue, and the gene annotation is shown in black (forward strand) and gray (reverse strand). The size of the respective ORF is also indicated.

Nine genes were chosen because of two factors: (1) strong upregulation of more than fourfold (8.6–42.3-fold), and 2) a high base mean (as a measure of the overall expression level in low salt). Gene HVO_0772 was regulated only 4.2-fold, it was added because it encodes a transcription factor that might turn out to be a hub for low-salt induced gene regulation. Gene HVO_2983_A was added because it encodes a very small protein of only 38 amino acids, and because it is a zinc finger protein that had already been studied by us in the course of another project (Üresin et al., in preparation). In all cases the Integrated Genome Browser (IGB) was used to confirm that the read distributions covered the whole ORFs and were compatible with the calculated induction levels. Three examples are shown in Figure 4 to visualize the genomic organization and the differential regulation in low salt. The transcript of HVO_0772 is leaderless (as typical for haloarchaea) and has a rather short 3’-UTR. The transcript level is very high in low salt, which is unusual for a transcription factor. The transcript of HVO_1863 is more than twice as long as the ORF. The very long 3’-UTR might be involved in translational control or regulation of transcript stability. The transcript level of B0276 is very low and it was induced only after 68 h, long after the culture had started to grow. Both facts could shed doubts on a putative essential function of B0276 in low salt. Remarkably, despite their very different features all three genes are of importance for low salt adaptation (see below).

In several cases the IGB browser revealed that the calculation of induction levels can result in false-positives or false negatives. For example, HVO_2578 was calculated to be induced, however, analysis of the sequencing results in the IGB revealed that many reads in fact belonged to a long 3’-UTR of the neighboring gene, HVO_2579. In contrast, the gene cluster HVO_2579-2581 was not calculated as regulated because it slightly failed the significance test in the control versus low salt comparison. However, it was very highly induced in the 68 h versus 26 h comparison, with high statistical significance. The IGB showed that the three genes were induced under low-salt, and, in addition, they had very high transcript levels (Supplementary Figure 3). The lack of significance was based on the variability in the control sample in this case. Therefore, this gene cluster was added to the mutant analysis. Two further examples of induced clusters of genes are HVO_A0205-A0211 encoding the Cas genes and the very large gene cluster HVO_B0048-B0066 encoding “cobalt associated genes” (Supplementary Figure 4). Due to the large number of genes, they were not added to the mutant analysis.

Taken together, in total 11 genes and one gene cluster (HVO_2579–2581; Supplementary Figure 3) were selected for mutant analysis. They are listed in Table 1 together with their regulation levels, protein names and functional categories. Detailed data from the RNA-Seq analysis and screenshots from the IGB for all 11 genes can be found in Supplementary Figure 5. Taken together, the verification of the bioinformatic analysis of differential regulation by analyzing the read distribution turned out to be important to avoid false negative and false positives as well as for the selection of highly-regulated genes.

**TABLE 1:** Selected genes for in frame-deletion and further analysis (FC, function class, see Supplementary Figure 1; AA, amino acids).

Name	Condition	Fold change	FC	AA	Protein name
HVO_0665	0.9M/26 h vs. Ctrl	0.21	COM	307	Adenosine diphosphate thiazole synthase. cysteine-dependent
	0.9M/68 h vs. Ctrl	30.93			
HVO_0772	0.9M/26 h vs. Ctrl	4.17	REG	90	NP_1176A family transcription regulator
	0.9M/68 h vs. Ctrl	2.62			
HVO_0777	0.9M/26 h vs. Ctrl	10.17	GEN	93	HalOD1 domain protein
	0.9M/68 h vs. Ctrl	10.15			
HVO_1561	0.9M/26 h vs. Ctrl	3.95	CHY	57	Conserved hypothetical protein
	0.9M/68 h vs. Ctrl	14.24			
HVO_1556	0.9M/26 h vs. Ctrl	not	CHY	78	Conserved hypothetical protein
	0.9M/68 h vs. Ctrl	15.92			
HVO_1863	0.9M/26 h vs. Ctrl	0.30	CHY	81	Conserved hypothetical protein
	0.9M/68 h vs. Ctrl	8.57			
HVO_2447A	0.9M/26 h vs. Ctrl	not	CHY	88	Conserved hypothetical protein
	0.9M/68 h vs. Ctrl	10.13			
HVO_2583A	0.9M/26 h vs. Ctrl	not	CHY	66	Conserved hypothetical protein
	0.9M/68 h vs. Ctrl	32.84			
HVO_2955A	0.9M/26 h vs. Ctrl	not	CHY	35	Conserved hypothetical protein
	0.9M/68 h vs. Ctrl	20.84			
HVO_2983_A	0.9M/26 h vs. Ctrl	not	CHY	38	Conserved hypothetical protein
	0.9M/68 h vs. Ctrl	2.68			
HVO_B0276	0.9M/26 h vs. Ctrl	not	TP	323	DMT superfamily transport protein
	0.9M/68 h vs. Ctrl	42.16			

Unexpectedly, nine of the 12 genes/gene clusters encode very small proteins (μ-proteins) of less than 100 amino acids. Seven of these are annotated as “conserved hypothetical proteins,” indicating that the majority of proteins that are most important for low salt adaptation have not been studied yet. In addition, it underlines that many μ-proteins may fulfill important functions during growth in low salt.

### Generation and characterization of *in frame* deletion mutants of genes and operons

The well-established Pop-In/Pop-Out method was used to generate *in frame* deletion mutants of the selected genes/operons (Allers et al., 2004; Hammelmann and Soppa, 2008). The presence of homozygous mutants was verified using multi-cycle PCR and Northern blot analysis. The multi-cycle PCR analysis was regularly repeated at the start of new experiments.

**FIGURE 5:**
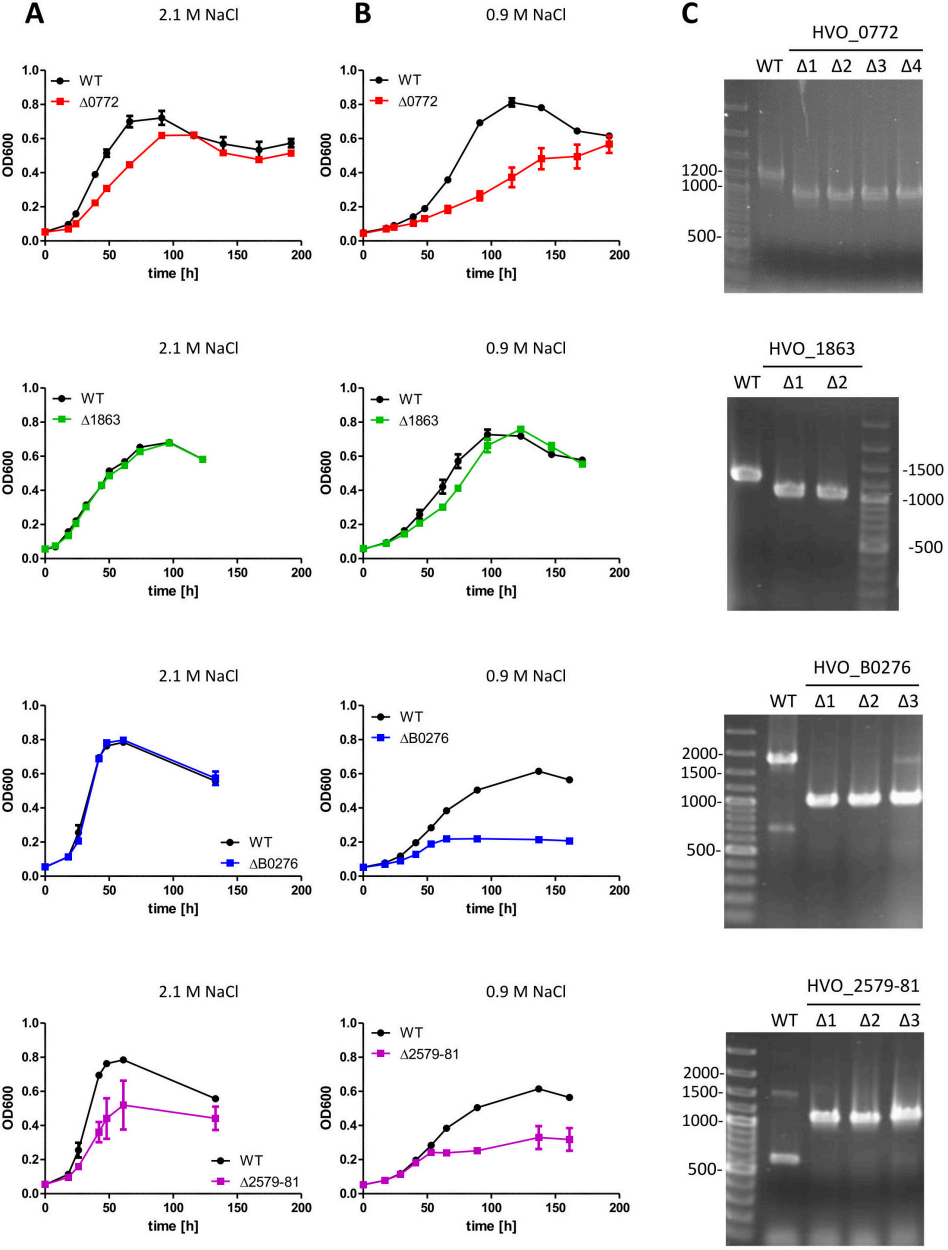
*H. volcanii* H26 wildtype cultures (black curves) and the indicated *in frame* deletion mutants (colored curves) were grown in synthetic glucose medium with the optimal NaCl concentration of 2.1 M **(A**, left panels) or 0.9 M **(B**, middle panels). Cultures were inoculated with a strt OD_600_ of 0.05. The OD_600_ values were recorded at the indicated time points. Average values and their standard deviation representative for three individual experiments are shown. The number of established and investigated deletion mutants was four for ΔHVO_0772, two for ΔHVO_1863, three for ΔHVO_B0276 and ΔHVO_2579–81. **(C)** Multicycle PCR was used to verify the internal deletions of the respective genes at the end of the growth experiment.

Growth of the mutants was characterized in synthetic glucose medium with the low salt concentration of 0.9 M, compared to that with the optimal concentration of 2.1 M. Several independent clones were used for the three biological replicates to exclude that unwanted additional chromosomal mutations might influence the phenotype. The growth of eight of the mutants in low salt was identical to that of the wildtype (Supplementary Figure 6). However, in four cases growth of wildtype and the respective mutant differed at low salt (Figure 5). One example is the deletion mutant of transcription factor HVO_0772, which grows worse than the wildtype at both salt concentrations, however, the defect is much more severe in low salt. A second example is the small 81 aa conserved hypothetical protein HVO_1863. The mutant has a slight, but significant and reproducible growth defect in low salt, which is not present at the optimal salt concentration. The third and most notable example is the “transport protein” HVO_B0276, which shows only very marginal growth in low salt, but does not have any growth defect at 2.1 M NaCl. In light of the low transcript level and the late induction (see above), this result was very unexpected. The fourth example is the operon HVO_2579–2581, which has a growth defect under both conditions, however, the defect is somewhat more severe in low salt. Due to the growth defect also at the optimal growth condition and because complementation would have required the simultaneous production of several proteins, the operon mutant was excluded from further analyses.

### Complementation of selected deletion mutants

The ORFs of the three remaining genes were cloned into the haloarchaeal expression vector pSZ, a modified version of the vector pSD1-R1/6. Thereby the ORFs were placed downstream of a strong constitutive promoter (Danner and Soppa, 1996). Sequences for either an N-terminal or a C-terminal hexahistidin tag were added during cloning, so that two different expression plasmids were generated for each mutant. The resulting plasmids were used to transform the respective deletion mutants. The empty vector pSZ was used as a negative control, because we have noticed previously that in some cases the presence of an empty vector can influence the phenotype of *H. volcanii*.

Unexpectedly, we were unable to transform the deletion mutant HVO_1863. Both plasmid variants resulted in extremely few clones after transformation (Supplementary Figure 7). Northern blot analysis revealed that the surviving clones did not contain the HVO_1863 transcript, therefore, it appears that silencing mutations are necessary to survive the presence of the plasmid. It is tempting to speculate that the expression vectors with the strong promoter led to an increased transcript and protein level compared with the wild-type, and that this prevented growth of transformants without silencing mutations.

In contrast, the other expression plasmids resulted in normal transformation efficiencies. Therefore, it was possible to unravel whether the proteins with an N-terminal or a C-terminal tag (or both) can complement the phenotype of the respective deletion mutant.

**FIGURE 6:**
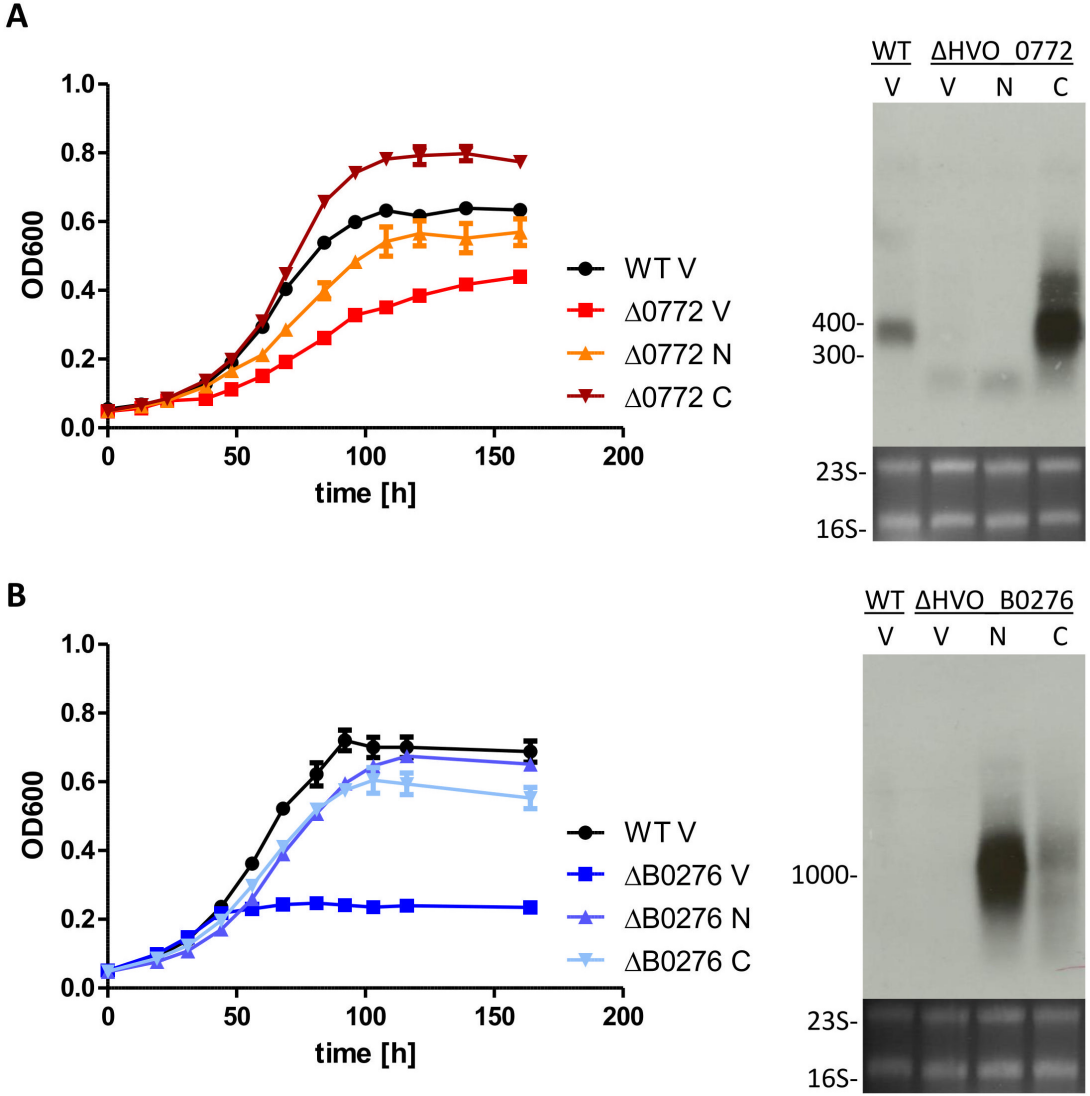
**(A)** Complementation of deletion mutant ΔHVO_0772. **(B)** Complementation of deletion mutant ΔHVO_B0276. Left panels: growth curves (OD_600_) are shown for the wildtype and the respective deletion mutant transformed with the empty vector (V) or the expression vector containing the gene with an N-terminal his-tag (N) or a C-terminal his-tag (C), as indicated. Three biological replicates were performed, and average values and their standard deviations are shown. Right panel: Northern blots for the respective gene. Cells of the four strains shown in the left panel were harvested under control condition with Novobiocin in exponential growth phase and RNA was extracted as described. Fragment sizes (bp) are indicated on the left.

The results for HVO_0772 are shown in Figure 6A. The growth defect of the deletion mutant could be verified, also in the presence of the empty vector (“V”). Presence of the N-terminally tagged protein could largely complement the phenotype, which was remarkable because the transcript level was very low to undetectable. In contrast, the transcript encoding the C-terminally tagged protein had a very high level, exceeding that of the wildtype transcript several fold. As a result, the plasmid-containing mutant grew even considerably better than the wildtype in low salt. Therefore, the ability to grow at 0.9 M NaCl apparently has a clear correlation to the transcript level of the transcription factor HVO_0772.

The very severe growth defect in low salt could be reproduced for the deletion mutant HVO_B0276 even in the presence of the empty vector (“V”). Also, in this case the transcript levels of the two tagged versions were very different, both exceeding the transcript level in the wildtype. Nevertheless, both variants were able to nearly fully complement the phenotype of the mutant. In this case, it seems as if a minimal protein level was needed to guarantee normal growth in low salt, and a higher amount does not add a further benefit (but does not hurt either).

Taken together, in two cases the phenotype of the deletion mutant could be complemented with the cognate gene in an expression vector, verifying that the phenotypes were not caused by secondary mutations in the genome or off-target effects. And in one case it was revealed that high levels of the gene product might be toxic to *H. volcanii*.

## Discussion

*H. volcanii* was isolated from the Dead Sea exactly 50 years ago and described as a moderatedly halophilic archaeon, which has a lower limit of 1.7 M NaCl to sustain growth (Mullakhanbhai and Larsen, 1975). For the subsequent decades, 1.7 M NaCl or 1.4 M NaCl were regarded as the lower limit for growth. Recently, we reported that for growth in microtiter plates in synthetic glucose medium, the actual lower limit is 0.7 M NaCl (Jantzer et al., 2011). Unfortunately, the salt input of the inoculum was not taken into account, and a re-calculation showed that the real NaCl concentration in the experiment was 0.9 M instead of the reported 0.7 M. In the current study we confirmed that *H. volcanii* can grow at 0.9 M NaCl, but cannot grow in 0.7 M NaCl despite very long adaptation times.

While we could show that 0.9 M NaCl is sufficient for growth both in microtiter plates as well as in Erlenmeyer flasks, there is a notable difference. In microtiter plates there was a lag phase of about 30 h (Figure 3 in Jantzer et al., 2011). In contrast, a long lag phase was not observed for growth in Erlenmeyer flasks (Figure 1). The most probable explanation for the different behavior is the very different oxygen availability in microtiter plates versus Erlenmeyer flasks.

For the characterization of the adaptation of *H. volcanii* to the lowest NaCl concentration that enables growth, transcriptome comparisons were performed between cells growing at the optimal NaCl concentration of 2.1 M NaCl, and at the lowest possible NaCl concentration of 0.9 M. We did not aim to study short-term adaptation processes that occur during the lag phase before the onset of growth, but to characterize long-term adaptation that enables exponential growth in low salt. The gene sets that are needed for these two processes are most likely not identical. In a previous project we had characterized short-term as well as long-term adaptation after the switch of the carbon source from casamino acids to glucose (Zaigler et al., 2003). Many genes were induced only during short-term adaptation and were thus needed only for the transition from one steady state to the other.

Notably, already in the past studies aimed at characterization of the adaptation of *H. volcanii* and other halophiles to different salinities. Examples are the studies by Ferrer et al. (1996), Mojica et al. (1997), and Bidle (2003). The studies used 1.6 M NaCl, 1.7 M NaCl, and 2.1 M NaCl as lowest salt concentrations, and the sequences of the differentially regulated genes did not become available.

For the identification of differentially regulated genes the determination of the genome sequence in 2010 was a breakthrough (Hartman et al., 2010). Since then various studies concentrating on single genes have been performed, but also several highly parallel OMICS studies. Examples for the latter are the studies of Babski et al. (2016), Moran-Reyna and Coker (2014), Gelsinger and DiRuggiero (2018), and Laass et al. (2019), and Lange et al. (2007). None of them included different salt concentrations.

However, a label-free proteomic analysis compared the proteomes of cultures grown at “low salt” (1.85 M NaCl), optimal salt (2.57 M NaCl), and high salt (3.29 M NaCl) (Jevtić et al., 2019), which will be discussed below. Differential protein glycosylation was discovered for cultures grown in low salt (1.75 M NaCl) and high salt (3.4 M NaCl) (Kaminski et al., 2013). An “Archaeal Proteome Project” was founded with the aim to collect all proteome analyses with *H. volcanii* at one website to give a comprehensive overview of proteome projects and to enable meta-analyses of the data^2^ (Schulze et al., 2020). Currently it contains 14 data sets with emphasis on different biological questions, e.g., characterization of the glycoproteome of *H. volcanii*.

However, neither any of the OMICs studies nor any gene-specific study used a NaCl concentration of less than 1.6 M. Therefore, the current study, i.e., the comprehensive characterization of the adaptation of the *H. volcanii* transcriptome to a salt concentration of as low as 0.9 M NaCl, is an unprecedented endeavor. The growth of *H. volcanii* is indistinguishable at NaCl concentrations of 1.2 M and above and the optimal concentration of 2.1 M NaCl.

However, the growth rate of *H. volcanii* is considerably lower at 0.9 M salt than at higher salt concentrations, therefore, it seems that not all biological processes of the cell have the same efficiency, but bottle necks exist that limit growth. We made an attempt to identify putative bottle necks using experimental evolution. *H. volcanii* cultures were incubated at 0.7 M/0.6 M/0.5 M NaCl for several weeks. However, no growth was observed under any of these conditions (unpublished results). Therefore, it seems that 0.9 M NaCl is a very stringent lower limit for growth of *H. volcanii*, at least in synthetic medium with glucose as sole carbon and energy source.

**FIGURE 7:**
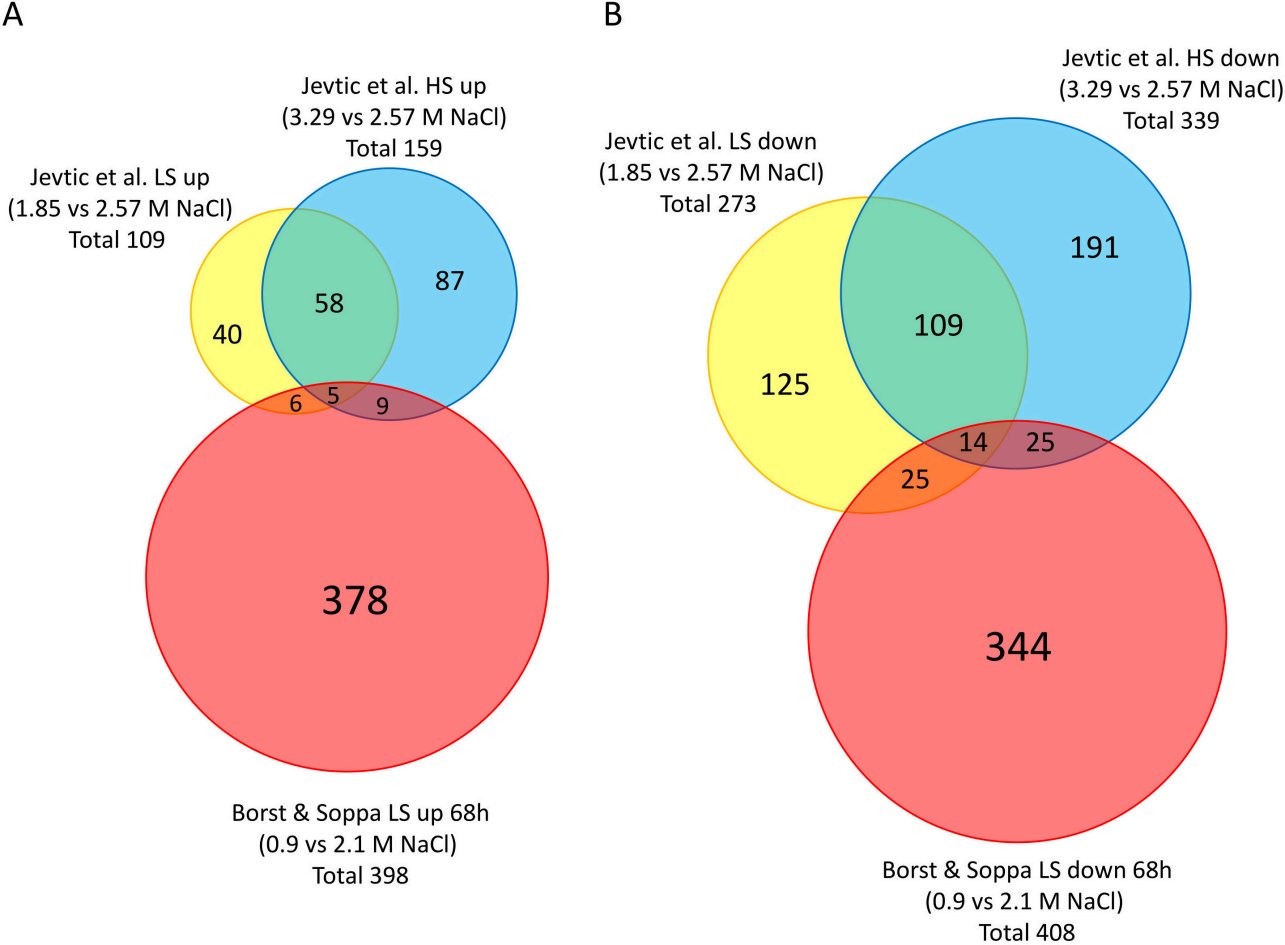
Venn diagrams comparing the transcriptome comparisons reported here with the proteome comparisons reported by Jevtić et al. (2019). **(A)** Comparison of unregulated transcripts and proteins, respectively. **(B)** Comparison of down-regulated transcripts and proteins, respectively. The source (this study or Jevtic et al.), the NaCl concentrations, and the numbers of transcripts or proteins are indicated. The sizes of the circles correspond to the numbers of regulated transcripts or proteins. The data reported by Jevtic et al. were reanalyzed using the same thresholds than in the current study (differential regulation at least twofold, *P* < 0.05).

About 400 genes were more than twofold upregulated and 400 genes more than twofold downregulated in 0.9 M compared to 2.1 M NaCl (Table 1). Together, these are nearly 20% of all 4,200 protein-coding genes of *H. volcanii*. To get an insight whether differential regulation of these genes is specific for the low salt stress, or whether they are generally salt-regulated, our transcriptome comparison of 2.1 M versus 0.9 M NaCl was compared to the proteome comparisons of 2.57 M versus 1.85 M NaCl and of 2.57 M versus 3.29 M NaCl reported by Jevtić et al. (2019). Figure 7 shows two Venn diagrams with comparisons of the upregulated and the down-regulated genes, respectively, using the same cut-off as in the present study (differential regulation at least twofold, *P* < 0.05). The number of differentially upregulated transcripts at 0.9 M NaCl is much higher than the number of upregulated proteins at 1.85 M NaCl or 3.29 M NaCl. And the overlap is marginal, indicating that low salt adaptation is very different from the variation of the NaCl concentration around the optimal concentration. Nevertheless, although the growth rates and growth yields are identical from 1.85 M NaCl to 3.29 M NaCl (compare Figure 3 in Jantzer et al., 2011), variation of the NaCl concentration resulted in a moderate change of the proteome.

It has been reported that two different glycosylation pathways exist, and the one of them is specific for the low salt concentration of 1.75 M NaCl, which is encoded by the gene cluster HVO_2046 to HVO_2061 (Kaminski et al., 2013). In contrast, a subsequent comprehensive glycoproteomics study revealed that the enzymes encoded by the “low salt cluster” are equally present at higher salt concentrations, and that both glycosylation pathways act simultaneously (Schulze et al., 2021). Nevertheless, the transcriptome data were analyzed to unravel whether an indication for salt-dependent differential regulation could be found. The genome contains 25 “archaeal glycosylation” (agl) genes, which are mainly concentrated on the gene clusters HVO_1517—HVO_1531 and HVO_2026—HVO_2061, respectively, with three exceptions. The analysis of the transcriptome data in the Integrated Genome Browser revealed that the transcript levels of all agl genes were extremely low at both salt concentrations. In addition, only four of 25 agl genes are included in Supplementary Table 3, two of each gene cluster. Taken together, the comparison of the transcriptomes at 0.9 M NaCl and 2.1 M NaCl did not yield any indication of differential glycosylation at low versus optimal salt.

From the 400 upregulated genes 11 genes and one gene cluster were selected for mutant analysis. Nine of the 11 selected proteins were very small (below 100 aa), With the sole exception of HVO_2983_A, the nine proteins were selected based on their high induction levels and their high transcript levels during growth in low salt. The high fraction of μ-protein genes among the genes with highest induction levels prompted us to analyze whether μ-protein genes might be enriched in the whole set of low salt-induced genes. However, this was not the case, the fraction in the whole set of differentially regulated genes was 16%, the same fraction as in the whole proteome.

The structures of the 11 selected proteins and the HVO 2579–81 cluster, as predicted by AlphaFold3, as well as protein details derived from the HaloLex database are shown in Supplementary Figure 8. Based on the results, three of these proteins seem to be of specific interest. HVO_1863 is a “conserved hypothetical protein” of 81 aa. It has a globular structure with two alpha helices, two loops and a flexible N-teminus. Blast searches in the NCBI non-redundant protein sequence database revealed that the 1000 closest hits are all orthologs from haloarchaea, therefore, the protein is highly conserved within this group. In contrast, no orthologs could be found within the Crenarchaeota, Bacteria, or Eukarya.

A multiple sequence alignment with the 99 most similar proteins revealed that seven positively charged amino acids (lysine/arginine) and five serines/threonines are 100% conserved (Supplementary Figure 9), which is highly enriched compared with the overall proteome. Under low salt its transcript is up-regulated 8.6-fold, which indicates its importance under this condition. Deletion of the cognate gene led to a slight, but consistent growth defect at 0.9 M NaCl. Strikingly, the protein could not be overproduced, but the presence of an expression vector with a strong promoter prevented the growth of transformants. If a high protein level would really be toxic, this would represent the first example that overproduction of a specific endogenous protein in *H. volcanii* results in cell death.

HVO_B0276 is annotated to be a “DMT superfamily transport protein.” Blast searches revealed that the 1,000 closest hits were all from haloarchaea. In Crenarchaeaota, only a single protein with convincing similarity was detected, and only three similar proteins in bacteria, indicating horizontal transfer. No similar proteins were found in Eukarya. The predicted structure consists of eight membrane-spanning helices and an additional helix in the cytoplasm or periplasm. The transcript is induced more than 40-fold in low salt, one of the highest induction level observed in the whole transcriptome. The deletion of the cognate gene resulted in a complete loss of the ability to grow at 0.9 M NaCl, obviously, it represents a central bottle neck at this salt concentration. In stark contrast, at 2.1 M NaCl the deletion mutant had absolutely no growth defect. Production of HVO_B0276 in the deletion mutant fully restored growth, even when the transcript level was extremely low, and, therefore, it is tempting to speculate that also the protein level was not very high. It would be interesting to unravel which transport function is so important solely under low salt concentrations.

HVO_0772 is annotated to be a transcription factor. Blast searches revealed that the 1,000 closest hits were all from haloarchaea. In contrast to the other two proteins mentioned above, many similar sequences were also found in Crenarchaeota. In addition, also 16 bacterial species contain similar transcription factors, albeit with lower similarity than within the Archaea. No similar proteins were found in the Eukarya. It has mainly a globular structure containing a helix-turn-helix motiv and a third helix. It also has an unstructured N-terminus. A multiple sequence alignment revealed that eight lysines/arginine are 100% conserved and two additional arginines are 90% conserved (Supplementary Figure 9), in agreement with its predicted role as a DNA-binding protein. In addition, five serines and one threonine are 100% conserved, indicating that this protein might be subject to posttranslational phosphorylation. The deletion mutant has only a small growth defect at 2.1 M NaCl, but a severe growth defect at 0.9 M NaCl. Strikingly, homologous overproduction resulted in an even enhanced growth rate and growth yield compared to the wildtype. Notably, with HVO_0772 we could identify a first example of a protein that is so crucial for growth at low salt that a higher level results in a “gain of function” phenotype. Many of the most similar proteins are annotated as “MarR-like transcription factors.” In bacteria, MarR is a transcription factor that is involved in stress adaptation (Shimizu, 2016; Wilkinson and Grove, 2006). However, transcription factors from the MarR family are involved in various regulation processes in different bacterial species, so that it is not possible to predict a specific regulatory role for a homologous protein in *H. volcanii*. In an attempt to identify a putative binding motif for HVO_0772, the 200 nt upstream of the 25 most highly low salt-induced genes were analyzed using MEME. Unfortunately, no enriched motif was found. Future studies will be needed to characterize the molecular regulatory mechanism of this predicted transcription factor.

## Conclusion

It could be clarified that the lower limit of growth for *H. volcanii* is 0.9 M NaCl, and no adaptation to 0.7 M NaCl was possible even after prolonged incubation. Transcriptome analyses revealed that about 10% of all genes were upregulated at 0.9 M NaCl compared to 2.1 M NaCl, indicating that the gene products were important for low salt adaptation, and 10% were downregulated. The adaptation is very slow and not complete after 26 h, but many additional genes were differentially regulated between 26 and 68 h. 12 *in frame* deletion mutants of low salt induced genes or gene clusters were generated. The lack of four of the corresponding gene products resulted in a growth defect at low salt, revealing their specific importance and bottle neck roles for growth under this condition. Among them was a membrane transporter, which was absolutely essential for growth solely at low salt, and a transcription factor of the MarR family. The current study gives an unprecedented insight into the adaptation of *H. volcanii* to the lowest NaCl concentration it is able to live in. It will enable further studies to unravel the molecular mechanisms of low-salt adaptation.

## Materials and methods

### Strains, media, and culture conditions

The *H. volcanii* H26 strain served as the wild type in this study and all further strains used in this study are based on it. The strains were grown in complex or synthetic media with glucose as sole carbon source (Allers et al., 2004; Dambeck and Soppa, 2008; Jantzer et al., 2011). All strains with expression plasmids derived from pSD1/pSZ were grown in the presence of Novobiocin (0.5 μg/mL) (Danner and Soppa, 1996). Varying NaCl concentrations were used, while other salts remained unchanged throughout the whole study.

The E. coli strain XL1-Blue MRF’ (Agilent Technologies, Waldbronn, Germany) was used for cloning and construction of all plasmids used in this stud

### Generation of *in frame* deletion mutants

All deletion mutants of *H. volcanii* used in this study have been constructed using the established Pop-In-Pop-Out method and the pMH101 vector (Allers et al., 2004; Hammelmann and Soppa, 2008). The oligonucleotides for mutant construction and for all other experimental approaches of this study are listed in Supplementary Table 1. The presence of the shortened ORF and absence of the native genomic locus has frequently been checked by multicycle PCR on isolated genomic DNA.

### Complementation of mutants

For the homologous expression, the ORF of the respective gene was cloned into the expression vector pSZ, containing a strong constitutive promoter. This vector is a modified version of the pSD1-R1/6 expression vector (Danner and Soppa, 1996), where the *Nco*I site is replaced by a *Nde*I restriction site (Zahn et al., 2021). The ORF of the respective gene containing either codons for a N-terminal or C-terminal hexa-Histidine-tag was cloned into the vector using *Nde*I and *Kpn*I restriction enzymes. Oligonucleotides used to amplify the gene from genomic DNA are also listed in Supplementary Table 1. The sequences of the resulting plasmids were verified by sequencing (GATC/Eurofins). PEG-mediated transformation was used to introduce the expression or empty vector in the desired *Haloferax volcanii* strain.

### Growth analyses

Growth assays were generally performed in 100 mL Erlenmeyer flasks (45 mL of culture volume; for RNA extraction and RNA-Seq) or test glasses (12 mL culture volume; for growth analysis of deletion mutants) to minimize evaporation during growth and keep the salt concentration stable over several days. Synthetic glucose medium was used with indicated NaCl concentration.

Before each growth experiment, a pre-culture was grown in glucose medium with 2.1 M NaCl to mid-exponential growth. To set the OD_600_ to 0.05 at the start of each growth experiment, an amount of the pre-culture was taken to obtain 4 × 10^7^ cells/ml for the final volume and cells were gently pelleted. The supernatant was discarded, and the cell-pellet resuspended in basal salts (1/100 of the final volume to not change the NaCl concentration significantly) and transferred to the flasks/glasses with the respective amount of glucose medium and gently mixed. Cells were grown for 120–200 h at 42°C in an orbital shaker with 250 rpm. The OD_600_ was frequently measured using the microtiter plate photometer Spectramax 340 (Molecular Devices, Ismaning, Germany). Therefore, every time 150 μL of each culture was taken and transferred to a 96-well microtiter plate. An uninoculated medium control was always used as a negative control.

### RNA isolation and RNA-Seq

*H. volcanii* cultures were grown as described above. Ten milliliter of cell culture was harvested after 26 h while 5 mL was taken after 68 h by centrifugation, and total RNA was isolated using phenol/chloroform extraction Afterward, the RNA was DNase-I (Thermo Fisher Scientific) digested and again purified with phenol/chloroform extraction. Total DNA removal is more complicated for the polyploid *H. volcanii*, which contains about 20 copies its chromosome, than for monoploid species. If necessary, the DNaseI treatment was repeated once. The absence of traces of DNA was verified by multi-cycle PCR, while 23S and 16S RNA integrity was confirmed on a RNA-gel. The RNA was shipped to StarSEQ GmbH (Mainz, Germany), where after another quality check was done (Qubit, RIN ≥ 7). StarSeq then performed rRNA depletion (NEBNext rRNA depletion Kit), library preparation (NEBNext Ultra II directional, dual index library), and RNA-Seq (Illumina Next Seq 500; 25 mio PE reads with 2 × 150 nt). The results were transmitted as fastq files.

### Differential expression analysis of RNA-Seq results

All downstream analysis of the RNA-Seq results were done using the galaxy platform.^3^ The “FastQC” tool (version 0.11.9) was used for quality control, while the “RNA STAR” tool (2.7.8a) was applied for mapping to the *Haloferax volcanii* reference genome (Hartman et al., 2010) and soft-clipping of non-matching sequences. Standard parameters were applied, except for the following: “Maximum ratio of mismatches to mapped length,” which was set to 0.04; “Minimum alignment score, normalized to read length” and “Minimum number of matched bases, normalized to read length,” which were set to 0.66, respectively. Afterward, the tool “htseq-count” (version 0.9.1) was used for counting all mapped reads of annotated genes. An up-to-date annotation file (September 2023) was thankfully provided by Friedhelm Pfeiffer of the Max Planck Institute of Biochemistry in Martinsried (Munich, Germany). Finally, differential expression was calculated with the “DESeq2” tool (2.11.40.8), and the data were exported for further procession to Microsoft Excel. Significance was obtained by filtering for results with adjusted *p* < 0.05 and at least a two-fold up- or downregulation. For visualization, the “bamCoverage” tool (version 3.5.1.0.0) was used to create normalized bigwig files, which can be accessed with the Integrated Genome Browser. Volcano plots were generated using the “create a volcano plot” tool (Galaxy Version 4.0.0). Raw sequencing data in FASTQ format and the normalized bigwig files are available through the Gene Expression Omnibus (GEO)^4^ under accession number GSE278471.

### Northern blot analyses

Cells were grown to mid-exponential growth phase (about 4–6 × 10^8^ cells/mL), harvested and RNA extracted with phenol/chloroform extraction. For expression level analysis, Northern blots were performed as described previously.^5^ Primers for generation of Digoxigenin-labeled probes of selected genes are listed in Supplementary Table 1. For probe detection, an anti-DIG antibody coupled to alkaline phosphatase was applied and the chemiluminescence substrate CDP star was used according to the manufacturer’s instructions (Roche, Mannheim, Germany). Signals were visualized using Xray films (GE Healthcare), and the sizes compared with the RiboRuler Low Range or high Range RNA Ladder (Thermo Fisher Scientific).

### Databases and bioinformatics analyses

The HaloLex database was used as a resource for nucleotide and protein sequences^6^ (Pfeiffer et al., 2008). The access is restricted for safety reasons, please contact Friedhelm Pfeiffer to gain access (fpf@biochem.mpg.de). Visualization of transcript reads was done with the Integrated Genome Browser (IGB) (Freese et al., 2016; Nicol et al., 2009). Homologous sequences were searched with BlastP provided by NCBI (Altschul et al., 1990). Multiple sequence alignment was performed with the ClustalOmega and MView tool from the EMBL-EBI site^7^ (Sievers et al., 2011). Protein structure predictions were generated using AlphaFold 3^8^ (Abramson et al., 2024). Statistical analysis for over- and underrepresented function classes was done via the galaxy platform and the “goseq—tests for overrepresented gene categories” tool (Galaxy Version 1.50.0) and the Wallenius non-central hypergeometric distribution method.

## Data Availability

The datasets presented in this study can be found in online repositories. The names of the repository/repositories and accession number(s) can be found at: https://www.ncbi.nlm.nih.gov/geo/, GSE278471.

## Footnotes

1
https://www.starseq.com/

2
https://archaealproteomeproject.org/

3
https://usegalaxy.eu/

4
https://www.ncbi.nlm.nih.gov/geo

5
https://protocolexchange.researchsquare.com/article/pex-535/v1

6
https://www.halolex.mpg.de

7
https://www.ebi.ac.uk/jdispatcher/msa/clustalo

8
https://alphafoldserver.com

## References

[B1] Abramson J. Adler J. Dunger J. Evans R. Green T. Pritzel A. (2024). Accurate structure prediction of biomolecular interactions with AlphaFold 3. *Nature* 630 493–500. 10.1038/s41586-024-07487-w 38718835 PMC11168924

[B2] Alam M. Lebert M. Oesterhelt D. Hazelbauer G. L. (1989). Methyl-accepting taxis proteins in *Halobacterium halobium*. *EMBO J.* 8 631–639. 10.1002/j.1460-2075.1989.tb03418.x 2721495 PMC400850

[B3] Allers T. Ngo H.-P. Mevarech M. Lloyd R. G. (2004). Development of additional selectable markers for the halophilic archaeon *Haloferax volcanii* based on the *leuB* and *trpA* genes. *Appl. Environ. Microbiol.* 70 943–953. 10.1128/AEM.70.2.943-953.2004 14766575 PMC348920

[B4] Altschul S. F. Gish W. Miller W. Myers E. W. Lipman D. J. (1990). Basic local alignment search tool. *J. Mol. Biol.* 215 403–410. 10.1016/S0022-2836(05)80360-2 2231712

[B5] Babski J. Haas K. A. Näther-Schindler D. Pfeiffer F. Förstner K. U. Hammelmann M. (2016). Genome-wide identification of transcriptional start sites in the haloarchaeon *Haloferax volcanii* based on differential RNA-Seq (dRNA-Seq). *BMC Genomics* 17:629. 10.1186/s12864-016-2920-y 27519343 PMC4983044

[B6] Babski J. Maier L.-K. Heyer R. Jaschinski K. Prasse D. Jäger D. (2014). Small regulatory RNAs in Archaea. *RNA Biol.* 11 484–493. 10.4161/rna.28452 24755959 PMC4152357

[B7] Bidle K. A. (2003). Differential expression of genes influenced by changing salinity using RNA arbitrarily primed PCR in the archaeal halophile *Haloferax volcanii*. *Extremophiles* 7 1–7. 10.1007/s00792-002-0289-0 12579374

[B8] Couto-Rodríguez R. L. Koh J. Chen S. Maupin-Furlow J. A. (2023). Insights into the Lysine Acetylome of the Haloarchaeon *Haloferax volcanii* during Oxidative Stress by Quantitative SILAC-Based Proteomics. *Antioxidants* 12:1203. 10.3390/antiox12061203 37371933 PMC10294847

[B9] Dambeck M. Soppa J. (2008). Characterization of a *Haloferax volcanii* member of the enolase superfamily: deletion mutant construction, expression analysis, and transcriptome comparison. *Arch. Microbiol.* 190 341–353. 10.1007/s00203-008-0379-1 18493744

[B10] Danner S. Soppa J. (1996). Characterization of the distal promoter element of halobacteria in vivo using saturation mutagenesis and selection. *Mol. Microbiol.* 19 1265–1276. 10.1111/j.1365-2958.1996.tb02471.x 8730868

[B11] Elazari-Volcani B. (1940). Algæ in the bed of the dead sea. *Nature* 145:975. 10.1038/145975a0

[B12] Elazari-Volcani B. (1943). Bacteria in the bottom sediments of the dead sea. *Nature* 152 274–275. 10.1038/152274c0

[B13] Elazari-Volcani B. (1944). A ciliate from the dead sea. *Nature* 154:335. 10.1038/154335a0

[B14] Ferrer C. Mojica F. J. Juez G. Rodríguez-Valera F. (1996). Differentially transcribed regions of *Haloferax volcanii* genome depending on the medium salinity. *J. Bacteriol.* 178 309–313. 10.1128/jb.178.1.309-313.1996 8550436 PMC177657

[B15] Freese N. H. Norris D. C. Loraine A. E. (2016). Integrated genome browser: visual analytics platform for genomics. *Bioinformatics* 32 2089–2095. 10.1093/BIOINFORMATICS/BTW069 27153568 PMC4937187

[B16] Gelsinger D. R. DiRuggiero J. (2018). Transcriptional landscape and regulatory roles of small noncoding RNAs in the oxidative stress response of the haloarchaeon *Haloferax volcanii*. *J. Bacteriol.* 200:e00779-17. 10.1128/JB.00779-17. 29463600 PMC5892119

[B17] Guan Z. Naparstek S. Calo D. Eichler J. (2012). Protein glycosylation as an adaptive response in Archaea: growth at different salt concentrations leads to alterations in *Haloferax volcanii* S-layer glycoprotein N-glycosylation. *Environ. Microbiol.* 14 743–753. 10.1111/j.1462-2920.2011.02625.x 22029420 PMC3414426

[B18] Gunde-Cimerman N. Plemenitaš A. Oren A. (2018). Strategies of adaptation of microorganisms of the three domains of life to high salt concentrations. *FEMS Microbiol. Rev.* 42 353–375. 10.1093/femsre/fuy009 29529204

[B19] Hammelmann M. Soppa J. (2008). Optimized generation of vectors for the construction of *Haloferax volcanii* deletion mutants. *J. Microbiol. Methods* 75 201–204. 10.1016/j.mimet.2008.05.029 18582505

[B20] Harding T. Brown M. W. Simpson A. G. B. Roger A. J. (2016). Osmoadaptative strategy and its molecular signature in obligately halophilic heterotrophic protists. *Genome Biol. Evol.* 8 2241–2258. 10.1093/gbe/evw152 27412608 PMC4987115

[B21] Hartman A. L. Norais C. Badger J. H. Delmas S. Haldenby S. Madupu R. (2010). The complete genome sequence of *Haloferax volcanii* DS2, a model archaeon. *PLoS One* 5:e9605. 10.1371/JOURNAL.PONE.0009605 20333302 PMC2841640

[B22] Heyer R. Dörr M. Jellen-Ritter A. Späth B. Babski J. Jaschinski K. (2012). High throughput sequencing reveals a plethora of small RNAs including tRNA derived fragments in *Haloferax volcanii*. *RNA Biol.* 9 1011–1018. 10.4161/rna.20826 22767255 PMC3495736

[B23] Hundt S. Zaigler A. Lange C. Soppa J. Klug G. (2007). Global analysis of mRNA decay in *Halobacterium salinarum* NRC-1 at single-gene resolution using DNA microarrays. *J. Bacteriol.* 189 6936–6944. 10.1128/JB.00559-07 17644597 PMC2045193

[B24] Jantzer K. Zerulla K. Soppa J. (2011). Phenotyping in the archaea: optimization of growth parameters and analysis of mutants of *Haloferax volcanii*. *FEMS Microbiol. Lett.* 322 123–130. 10.1111/j.1574-6968.2011.02341.x 21692831

[B25] Jevtić Ž Stoll B. Pfeiffer F. Sharma K. Urlaub H. Marchfelder A. (2019). The response of *Haloferax volcanii* to salt and temperature stress: a proteome study by label-free mass spectrometry. *Proteomics* 19:e1800491. 10.1002/pmic.201800491 31502396

[B26] Kaminski L. Guan Z. Yurist-Doutsch S. Eichler J. (2013). Two distinct N-glycosylation pathways process the *Haloferax volcanii* S-layer glycoprotein upon changes in environmental salinity. *mBio* 4:e00716-13. 10.1128/mBio.00716-13 24194539 PMC3892788

[B27] Laass S. Monzon V. A. Kliemt J. Hammelmann M. Pfeiffer F. Förstner K. U. (2019). Characterization of the transcriptome of *Haloferax volcanii*, grown under four different conditions, with mixed RNA-Seq. *PLoS One* 14:e0215986. 10.1371/journal.pone.0215986 31039177 PMC6490895

[B28] Lange C. Zaigler A. Hammelmann M. Twellmeyer J. Raddatz G. Schuster S. C. (2007). Genome-wide analysis of growth phase-dependent translational and transcriptional regulation in halophilic archaea. *BMC Genomics* 8:415. 10.1186/1471-2164-8-415 17997854 PMC3225822

[B29] Mojica F. J. Cisneros E. Ferrer C. Rodríguez-Valera F. Juez G. (1997). Osmotically induced response in representatives of halophilic prokaryotes: the bacterium *Halomonas elongata* and the archaeon *Haloferax volcanii*. *J. Bacteriol.* 179 5471–5481. 10.1128/jb.179.17.5471-5481.1997 9287003 PMC179419

[B30] Moran-Reyna A. Coker J. A. (2014). The effects of extremes of pH on the growth and transcriptomic profiles of three haloarchaea. *F1000Res* 3:168. 10.12688/f1000research.4789.2 25285207 PMC4176423

[B31] Mullakhanbhai M. F. Larsen H. (1975). *Halobacterium volcanii* spec. nov., a Dead Sea halobacterium with a moderate salt requirement. *Arch. Microbiol.* 104 207–214. 10.1007/BF00447326 1190944

[B32] Nicol J. W. Helt G. A. Blanchard S. G. Raja A. Loraine A. E. (2009). The Integrated Genome Browser: free software for distribution and exploration of genome-scale datasets. *Bioinformatics* 25 2730–2731. 10.1093/bioinformatics/btp472 19654113 PMC2759552

[B33] Oren A. (2006). “The Order Halobacteriales,” in *The Prokaryotes*, eds Dworkin M. Falkow S. Rosenberg E. Schleifer K.-H. Stackebrandt E. (New York, NY: Springer), 113–164.

[B34] Pfeiffer F. Broicher A. Gillich T. Klee K. Mejía J. Rampp M. (2008). Genome information management and integrated data analysis with HaloLex. *Arch. Microbiol.* 190 281–299. 10.1007/s00203-008-0389-z 18592220 PMC2516542

[B35] Schlesner M. Miller A. Besir H. Aivaliotis M. Streif J. Scheffer B. (2012). The protein interaction network of a taxis signal transduction system in a halophilic archaeon. *BMC Microbiol.* 12:272. 10.1186/1471-2180-12-272 23171228 PMC3579733

[B36] Schulze S. Adams Z. Cerletti M. Castro R. de Ferreira-Cerca S. (2020). The Archaeal Proteome Project advances knowledge about archaeal cell biology through comprehensive proteomics. *Nat. Commun.* 11:3145. 10.1038/s41467-020-16784-7 32561711 PMC7305310

[B37] Schulze S. Pfeiffer F. Garcia B. A. Pohlschroder M. (2021). Comprehensive glycoproteomics shines new light on the complexity and extent of glycosylation in archaea. *PLoS Biol.* 19:e3001277. 10.1371/journal.pbio.3001277 34138841 PMC8241124

[B38] Shimizu K. (2016). Metabolic regulation and coordination of the metabolism in bacteria in response to a variety of growth conditions. *Adv. Biochem. Eng. Biotechnol.* 155 1–54. 10.1007/10_2015_320 25712586

[B39] Sievers F. Wilm A. Dineen D. Gibson T. J. Karplus K. Li W. (2011). Fast, scalable generation of high-quality protein multiple sequence alignments using Clustal Omega. *Mol. Syst. Biol.* 7:539. 10.1038/msb.2011.75 21988835 PMC3261699

[B40] Silva R. T. de Abdul-Halim M. F. Pittrich D. A. Brown H. J. Pohlschroder M. (2021). Improved growth and morphological plasticity of *Haloferax volcanii*. *Microbiology* 167:001012. 10.1099/mic.0.001012 33459585 PMC8131023

[B41] Sleator R. D. Hill C. (2002). Bacterial osmoadaptation: the role of osmolytes in bacterial stress and virulence. *FEMS Microbiol. Rev.* 26 49–71. 10.1111/j.1574-6976.2002.tb00598.x 12007642

[B42] Smith S. C. Kennelly P. J. Potts M. (1997). Protein-tyrosine phosphorylation in the Archaea. *J. Bacteriol.* 179 2418–2420. 10.1128/jb.179.7.2418-2420.1997 9079930 PMC178981

[B43] Soppa J. (2010). Protein acetylation in archaea, bacteria, and eukaryotes. *Archaea* 2010:820681. 10.1155/2010/820681 20885971 PMC2946573

[B44] Tripepi M. Imam S. Pohlschröder M. (2010). *Haloferax volcanii* flagella are required for motility but are not involved in PibD-dependent surface adhesion. *J. Bacteriol.* 192 3093–3102. 10.1128/JB.00133-10 20363933 PMC2901708

[B45] Wanner C. Soppa J. (1999). Genetic identification of three ABC transporters as essential elements for nitrate respiration in *Haloferax volcani*i. *Genetics* 152 1417–1428. 10.1093/genetics/152.4.1417 10430572 PMC1460679

[B46] Wilkansky B. (1936). Life in the Dead Sea. *Nature* 138:467. 10.1038/138467a0

[B47] Wilkinson S. P. Grove A. (2006). Ligand-responsive transcriptional regulation by members of the MarR family of winged helix proteins. *Curr. Issues Mol. Biol.* 8 51–62. 10.21775/cimb.008.051 16450885

[B48] Zahn S. Kubatova N. Pyper D. J. Cassidy L. Saxena K. Tholey A. (2021). Biological functions, genetic and biochemical characterization, and NMR structure determination of the small zinc finger protein HVO_2753 from *Haloferax volcanii*. *FEBS J.* 288 2042–2062. 10.1111/febs.15559 32905660

[B49] Zaigler A. Schuster S. C. Soppa J. (2003). Construction and usage of a onefold-coverage shotgun DNA microarray to characterize the metabolism of the archaeon *Haloferax volcanii*. *Mol. Microbiol.* 48 1089–1105. 12753198 10.1046/j.1365-2958.2003.03497.x

