# Supplementary material for: Studying the long-term adaptation of Haloferax volcanii to low salt conditions: transcriptomic and genetic analyses
Source: Front Microbiol. 2026 Jan 15;16:1697018. doi: 10.3389/fmicb.2025.1697018 (PMC12852389; doi:10.3389/fmicb.2025.1697018)
Supplement: Supplementary file 1 [file Data_Sheet_1.pdf]

Function Class	# of Genes assigned to Class	26h Low Salt vs Ctrl		68h Low Salt vs Ctrl		68h Low Salt vs 26h Low Salt	
		%up	%down	%up	%down	%up	%down
AA	132	22,0%	11,4%	21,2%	14,4%	6,8%	11,4%
CE	2	0,0%	50,0%	0,0%	50,0%	0,0%	0,0%
CHM	44	2,3%	25,0%	4,5%	22,7%	4,5%	6,8%
CHP	11	9,1%	9,1%	18,2%	9,1%	18,2%	0,0%
CHY	901	5,7%	10,5%	7,3%	6,3%	10,8%	4,1%
CIM	82	6,1%	17,1%	4,9%	26,8%	7,3%	24,4%
COM	114	20,2%	14,0%	21,9%	3,5%	17,5%	8,8%
CP	20	0,0%	5,0%	0,0%	10,0%	5,0%	5,0%
EM	76	9,2%	19,7%	3,9%	11,8%	21,1%	27,6%
GEN	1316	5,5%	12,1%	10,7%	8,2%	11,2%	5,1%
HY	25	0,0%	0,0%	0,0%	4,0%	0,0%	4,0%
ISH	111	1,8%	3,6%	2,7%	4,5%	0,0%	0,9%
LIP	54	9,3%	0,0%	7,4%	20,4%	7,4%	33,3%
MIS	286	7,0%	10,1%	11,9%	11,5%	9,1%	7,0%
MOT	4	0,0%	0,0%	0,0%	0,0%	0,0%	0,0%
NUM	50	28,0%	8,0%	6,0%	24,0%	6,0%	38,0%
REG	141	9,2%	14,9%	7,1%	11,3%	12,8%	8,5%
RMT	49	4,1%	4,1%	4,1%	6,1%	6,1%	6,1%
RRR	92	3,3%	2,2%	10,9%	3,3%	6,5%	6,5%
SEC	15	0,0%	0,0%	0,0%	6,7%	6,7%	6,7%
SIG	63	7,9%	7,9%	1,6%	7,9%	3,2%	7,9%
TC	37	2,7%	2,7%	0,0%	10,8%	13,5%	13,5%
TL	106	0,9%	9,4%	0,0%	30,2%	1,9%	16,0%
TP	393	20,1%	7,1%	15,3%	12,5%	10,4%	22,6%
**ALL**	**4124**	**8,1%**	**10,5%**	**9,7%**	**9,9%**	**10,0%**	**9,0%**

Function Classes

Function classes used for halophilic archaea by our group.

AA: amino acid metabolism
CE: cell envelope
CHM: carbohydrate metabolism
CHP: chaperones
CHY: conserved hypothetical protein
CIM: central intermediary metabolism
COM: coenzyme metabolism
CP: cellular processes
EM: energy metabolism
GEN: general enzymatic function
HY: hypothetical protein
ISH: transposases and ISH-encoded proteins
LIP: lipid metabolism
MIS: miscellaneous
MOT: motility
NOF: no function assigned to experimentally identified protein
NUM: nucleotide metabolism
REG: gene regulation
RMT: RNA maturation
RRR: replication, repair, recombination
SEC: protein secretion
SIG: signal transduction
TC: transcription
TL: translation
TP: small molecule transport

significantly overrepresented
significantly underrepresented

Supplementary Figure S1: Overview of differentially regulated genes assigned to specific function classes. The functional class for each gene has been obtained from the Halolex database (https://www.halolex.mpg.de). The percentage of all at least two-fold significantly regulated genes of each function class has been calculated and compared for all three samples (Ctrl: 2.1 M NaCl; 26 h / Low Salt: 0.9 M). The total number of genes attributed to a function class is also listed for comparison. Statistical analysis for over- and underrepresented function classes was done via the galaxy platform and the “goseq - tests for overrepresented gene categories” tool (Galaxy Version 1.50.0) and the the Wallenius non-central hypergeometric distribution method.
