# Supplementary material for: Studying the long-term adaptation of Haloferax volcanii to low salt conditions: transcriptomic and genetic analyses
Source: Front Microbiol. 2026 Jan 15;16:1697018. doi: 10.3389/fmicb.2025.1697018 (PMC12852389; doi:10.3389/fmicb.2025.1697018)
Supplement: Supplementary file 2 [file Data_Sheet_2.pdf]

26 h Low Salt vs Control upregulated

category	over_represented_pvalue	under_represented_pvalue	numDEInCat	numInCat	p_adjust_over_represented	p_adjust_under_represented
TP	0,000	1,000	79	393	0,000	1,000
AA	0,000	1,000	29	132	0,000	1,000
NUM	0,000	1,000	14	50	0,000	1,000
COM	0,000	1,000	23	114	0,002	1,000
REG	0,168	0,898	13	141	0,840	1,000
CHY	0,390	0,669	51	901	1,000	0,979
EM	0,395	0,750	7	76	1,000	0,979
CHP	0,614	0,770	1	11	1,000	0,979
CE	1,000	0,783	0	2	1,000	0,979
MOT	1,000	0,667	0	4	1,000	0,979
LIP	0,639	0,544	5	54	1,000	0,906
HY	1,000	0,355	0	25	1,000	0,634
SIG	0,818	0,322	5	63	1,000	0,620
SEC	1,000	0,260	0	15	1,000	0,542
TC	0,943	0,228	1	37	1,000	0,517
RMT	0,935	0,189	2	49	1,000	0,473
CP	1,000	0,152	0	20	1,000	0,422
CIM	0,940	0,129	5	82	1,000	0,403
MIS	0,944	0,086	20	286	1,000	0,309
CHM	0,994	0,039	1	44	1,000	0,161
RRR	0,998	0,007	3	92	1,000	0,042
TL	1,000	0,002	1	106	1,000	0,018
ISH	1,000	0,001	2	111	1,000	0,015
GEN	1,000	0,000	73	1316	1,000	0,002

26 h Low Salt vs Control downregulated

category	over_represented_pvalue	under_represented_pvalue	numDEInCat	numInCat	p_adjust_over_represented	p_adjust_under_represented
CHM	0,002	1,000	11	44	0,045	1,000
GEN	0,007	0,995	159	1316	0,074	1,000
EM	0,009	0,996	15	76	0,074	1,000
CIM	0,018	0,992	14	82	0,112	1,000
REG	0,068	0,959	21	141	0,340	1,000
COM	0,089	0,949	16	114	0,372	1,000
CE	0,171	0,992	1	2	0,610	1,000
AA	0,274	0,812	15	132	0,857	1,000
MIS	0,434	0,645	29	286	1,000	1,000
CHP	0,686	0,698	1	11	1,000	1,000
MOT	1,000	0,671	0	4	1,000	1,000
TL	0,671	0,456	10	106	1,000	0,852
SIG	0,696	0,477	5	63	1,000	0,852
NUM	0,751	0,431	4	50	1,000	0,852
CP	0,875	0,399	1	20	1,000	0,852
CHY	0,765	0,275	95	901	1,000	0,686
SEC	1,000	0,206	0	15	1,000	0,573
RMT	0,964	0,119	2	49	1,000	0,370
TC	0,984	0,088	1	37	1,000	0,315
TP	0,959	0,062	28	393	1,000	0,257
HY	1,000	0,044	0	25	1,000	0,221
ISH	0,996	0,012	4	111	1,000	0,075
RRR	0,999	0,007	2	92	1,000	0,061
LIP	1,000	0,004	0	54	1,000	0,053

68 h Low Salt vs Control upregulated

category	over_represented_pvalue	under_represented_pvalue	numDEInCat	numInCat	p_adjust_over_represented	p_adjust_under_represented
AA	0,000	1,000	28	132	0,003	1,000
COM	0,000	1,000	25	114	0,003	1,000
TP	0,003	0,998	60	393	0,022	1,000
GEN	0,030	0,977	141	1316	0,189	1,000
MIS	0,193	0,856	34	286	0,966	1,000
CE	1,000	0,784	0	2	1,000	1,000
CHP	0,287	0,919	2	11	1,000	1,000
MOT	1,000	0,645	0	4	1,000	0,895
RRR	0,574	0,560	10	92	1,000	0,823
LIP	0,841	0,306	4	54	1,000	0,479
NUM	0,886	0,255	3	50	1,000	0,445
REG	0,827	0,267	10	141	1,000	0,445
CHY	0,809	0,231	66	901	1,000	0,444
SEC	1,000	0,217	0	15	1,000	0,444
HY	1,000	0,161	0	25	1,000	0,365
CHM	0,964	0,119	2	44	1,000	0,308
CP	1,000	0,121	0	20	1,000	0,308
RMT	0,962	0,123	2	49	1,000	0,308
EM	0,979	0,062	3	76	1,000	0,222
CIM	0,982	0,049	4	82	1,000	0,204
TC	1,000	0,028	0	37	1,000	0,141
SIG	0,999	0,006	1	63	1,000	0,037
ISH	0,999	0,003	3	111	1,000	0,021
TL	1,000	0,000	0	106	1,000	0,001

68 h Low Salt vs Control downregulated

category	over_represented_pvalue	under_represented_pvalue	numDEInCat	numInCat	p_adjust_over_represented	p_adjust_under_represented
TL	0,000	1,000	32	106	0,000	1,000
CIM	0,000	1,000	22	82	0,001	1,000
NUM	0,003	0,999	12	50	0,025	1,000
CHM	0,017	0,994	10	44	0,107	1,000
LIP	0,022	0,991	11	54	0,110	1,000
AA	0,093	0,944	19	132	0,386	1,000
TP	0,158	0,879	49	393	0,564	1,000
REG	0,185	0,881	16	141	0,577	1,000
CE	0,218	0,987	1	2	0,606	1,000
MIS	0,314	0,752	33	286	0,743	1,000
EM	0,327	0,793	9	76	0,743	1,000
TC	0,459	0,740	4	37	0,956	1,000
CP	0,634	0,651	2	20	1,000	1,000
CHP	0,708	0,673	1	11	1,000	1,000
SEC	0,811	0,522	1	15	1,000	1,000
HY	0,872	0,403	1	25	1,000	1,000
MOT	1,000	0,622	0	4	1,000	1,000
RMT	0,879	0,267	3	49	1,000	0,834
SIG	0,881	0,230	5	63	1,000	0,820
GEN	0,977	0,031	108	1316	1,000	0,127
ISH	0,993	0,021	5	111	1,000	0,105
COM	0,998	0,008	4	114	1,000	0,052
CHY	0,997	0,004	57	901	1,000	0,036
RRR	0,999	0,004	3	92	1,000	0,036

68 h Low Salt vs 26 h Low Salt upregulated

category	over_represented_pvalue	under_represented_pvalue	numDEInCat	numInCat	p_adjust_over_represented	p_adjust_under_represented
EM	0,002	0,999	16	76	0,049	0,999
COM	0,005	0,998	20	114	0,056	0,999
GEN	0,025	0,981	147	1316	0,211	0,999
REG	0,171	0,888	18	141	0,969	0,999
TP	0,194	0,851	41	393	0,969	0,999
CHP	0,279	0,922	2	11	1,000	0,999
TC	0,299	0,849	5	37	1,000	0,999
CHY	0,356	0,688	97	901	1,000	0,999
CE	1,000	0,844	0	2	1,000	0,999
MOT	1,000	0,684	0	4	1,000	0,999
SEC	0,777	0,576	1	15	1,000	0,960
MIS	0,569	0,516	26	286	1,000	0,921
LIP	0,750	0,431	4	54	1,000	0,846
CIM	0,755	0,390	6	82	1,000	0,846
CP	0,855	0,440	1	20	1,000	0,846
RRR	0,813	0,314	6	92	1,000	0,786
RMT	0,863	0,295	3	49	1,000	0,786
NUM	0,869	0,284	3	50	1,000	0,786
AA	0,876	0,205	9	132	1,000	0,786
CHM	0,918	0,227	2	44	1,000	0,786
SIG	0,976	0,085	2	63	1,000	0,426
HY	1,000	0,057	0	25	1,000	0,357
TL	1,000	0,001	2	106	1,000	0,012
ISH	1,000	0,000	0	111	1,000	0,000

68 h Low Salt vs 26 h Low Salt downregulated

category	over_represented_pvalue	under_represented_pvalue	numDEInCat	numInCat	p_adjust_over_represented	p_adjust_under_represented
TP	0,000	1,000	89	393	0,000	1,000
NUM	0,000	1,000	19	50	0,000	1,000
EM	0,000	1,000	21	76	0,000	1,000
LIP	0,000	1,000	18	54	0,000	1,000
CIM	0,001	0,999	20	82	0,007	1,000
TL	0,010	0,996	17	106	0,040	1,000
TC	0,176	0,926	5	37	0,627	1,000
REG	0,333	0,772	12	141	1,000	1,000
AA	0,502	0,608	15	132	1,000	1,000
HY	0,650	0,726	1	25	1,000	1,000
SEC	0,801	0,539	1	15	1,000	1,000
CE	1,000	0,754	0	2	1,000	1,000
MOT	1,000	0,605	0	4	1,000	1,000
COM	0,722	0,398	10	114	1,000	0,829
CP	0,887	0,375	1	20	1,000	0,829
RMT	0,865	0,291	3	49	1,000	0,764
CHP	1,000	0,306	0	11	1,000	0,764
CHM	0,909	0,215	3	44	1,000	0,672
SIG	0,926	0,156	5	63	1,000	0,558
RRR	0,983	0,042	6	92	1,000	0,173
MIS	0,990	0,017	20	286	1,000	0,102
CHY	0,996	0,007	37	901	1,000	0,060
ISH	1,000	0,000	1	111	1,000	0,001
GEN	1,000	0,000	67	1316	1,000	0,000

Supplementary Figure S2: Detailed Overview and Statistical analysis of differentially regulated genes assigned to specific function classes. Statistical analysis for over- and underrepresented function classes was done via the galaxy platform and the "goseq - tests for overrepresented gene categories" tool (Galaxy Version 1.50.0) and the the Wallenius non-central hypergeometric distribution method. The p vale and adjusted p value for over- and underrepresented function classes is shown as well as the number of regulated genes (numDEInCat) and total number of the genes (numInCat) in the respective function class. Significance for overrepresented categories is highlighted in green and for underrepresented categories in red.
