# Supplementary material for: Studying the long-term adaptation of Haloferax volcanii to low salt conditions: transcriptomic and genetic analyses
Source: Front Microbiol. 2026 Jan 15;16:1697018. doi: 10.3389/fmicb.2025.1697018 (PMC12852389; doi:10.3389/fmicb.2025.1697018)
Supplement: Supplementary file 3 [file Data_Sheet_3.pdf]

A

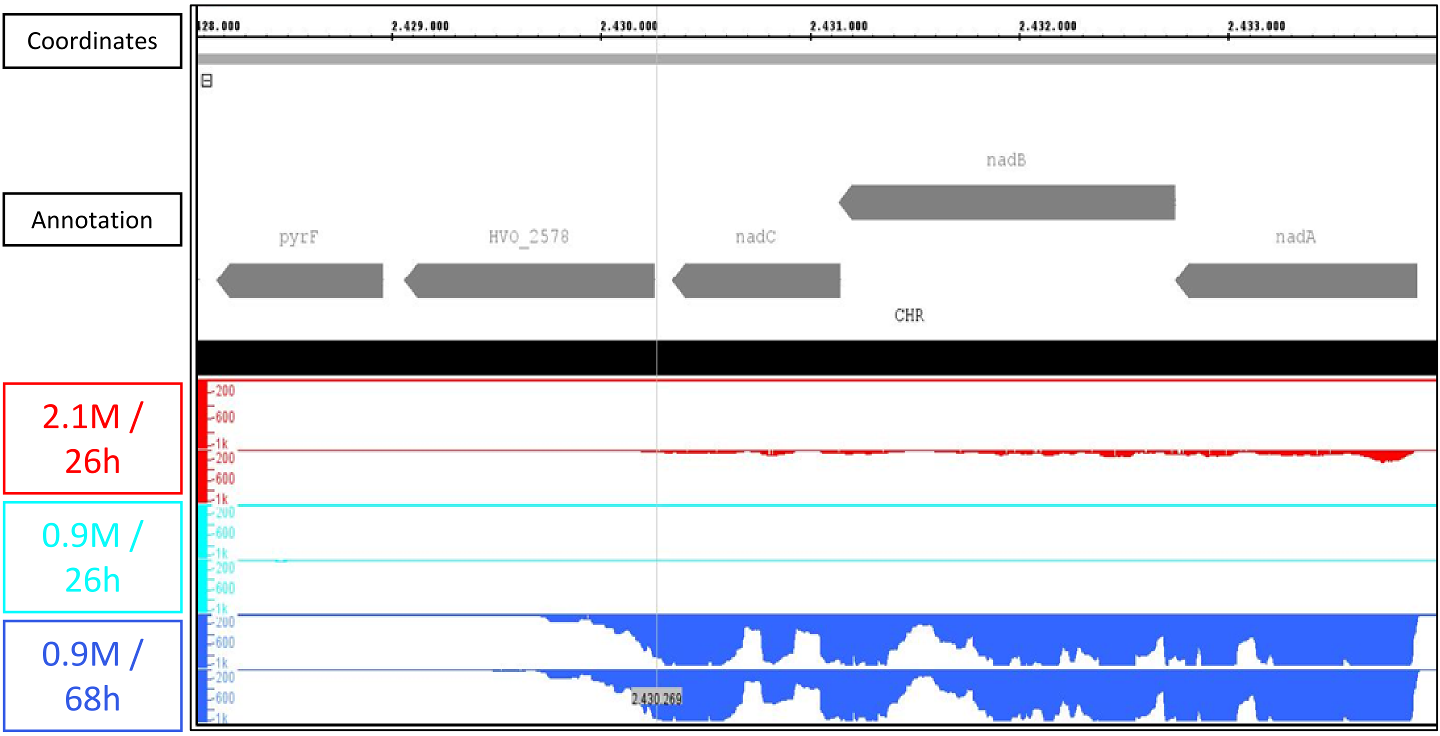

B

Name	Gene name	Base mean	log2(FC)	P-adj	fold change	
HVO_2578		105	-1,22	0,111	0,429	0.9M / 26h vs Ctrl
HVO_2579	nadC	267	-3,54	0,157	0,086	
HVO_2580	nadB	657	-3,69	0,198	0,078	
HVO_2581	nadA	829	-3,74	0,127	0,075	
Name	Gene name	Base mean	log2(FC)	P-adj	fold change	
HVO_2578		776	3,68	0,000	12,826	0.9M / 68h vs Ctrl
HVO_2579	nadC	2648	3,69	0,136	12,894	
HVO_2580	nadB	6498	3,67	0,202	12,755	
HVO_2581	nadA	7837	3,60	0,144	12,109	
Name	Gene name	Base mean	log2(FC)	P-adj	fold change	
HVO_2578		1434	4,87	0,000	29,310	0.9M / 68h vs 0.9M / 26h
HVO_2579	nadC	4771	7,21	0,000	148,115	
HVO_2580	nadB	11693	7,34	0,000	161,776	
HVO_2581	nadA	14045	7,32	0,000	159,517	

Supplementary Figure S3: Analyzing RNA-Seq data of the cluster HVO_2579-2581 via the Integrated genome browser (IGB). A) Shown are visualized read counts for the genes HVO_2578 and the cluster HVO_2579-81. The two replicates for the control condition are shown in red, for 26h low salt in teal, the 68 h low salt in blue and the gene annotation in grey (reverse). B) RNA-Seq data as obtained via the DESeq2 tool from the galaxy platform for the indicated genes and conditions. Genes with an non-significant adjusted p-value are marked red. The fold change as calculated from the log2(FC) is shown in green for upregulated genes.
