# Supplementary material for: Studying the long-term adaptation of Haloferax volcanii to low salt conditions: transcriptomic and genetic analyses
Source: Front Microbiol. 2026 Jan 15;16:1697018. doi: 10.3389/fmicb.2025.1697018 (PMC12852389; doi:10.3389/fmicb.2025.1697018)
Supplement: Supplementary file 4 [file Data_Sheet_4.pdf]

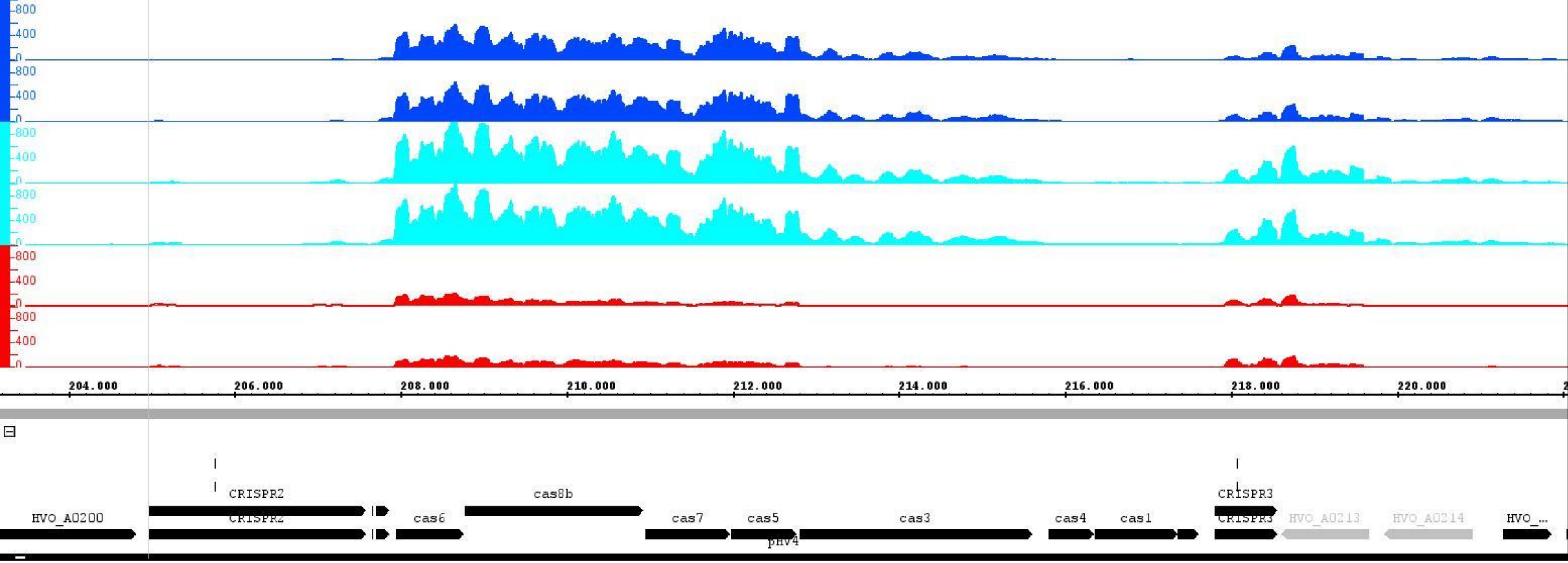

Name	Base mean	log2(FC)	P-adj	fold change	Fclass	AA	Gene name	Protein name	Condition
HVO_A0205	4425	1.67	0.000	3.18	MIS	272	cas6	CRISPR-associated endoribonuclease Cas6	0.9M/26h vs Ctrl
HVO_A0206	8803	2.20	0.000	4.58	MIS	717	cas8b	CRISPR-associated protein Cas8b	
HVO_A0207	2549	2.53	0.000	5.76	MIS	340	cas7	CRISPR-associated protein Cas7	
HVO_A0208	1945	2.52	0.000	5.74	MIS	265	cas5	CRISPR-associated protein Cas5, Hmari subtype	
HVO_A0209	1983	3.65	0.000	12.59	MIS	937	cas3	CRISPR-associated nuclease/helicase Cas3	
HVO_A0210	26	3.08	0.001	8.44	MIS	183	cas4	CRISPR-associated exonuclease Cas4	
HVO_A0211	55	1.42	0.025	2.67	MIS	331	cas1	CRISPR-associated endonuclease Cas1	

Name	Base mean	log2(FC)	P-adj	fold change	Fclass	AA	Gene name	Protein name	Condition
HVO_A0205	2655	1.18	0.002	2.26	MIS	272	cas6	CRISPR-associated endoribonuclease Cas6	0.9M/68h vs Ctrl
HVO_A0206	5231	1.73	0.000	3.32	MIS	717	cas8b	CRISPR-associated protein Cas8b	
HVO_A0207	1609	2.19	0.000	4.56	MIS	340	cas7	CRISPR-associated protein Cas7	
HVO_A0208	1336	2.33	0.000	5.04	MIS	265	cas5	CRISPR-associated protein Cas5, Hmari subtype	
HVO_A0209	1237	3.33	0.000	10.07	MIS	937	cas3	CRISPR-associated nuclease/helicase Cas3	
HVO_A0210	24	3.41	0.000	10.66	MIS	183	cas4	CRISPR-associated exonuclease Cas4	
HVO_A0211	47	1.60	0.020	3.03	MIS	331	cas1	CRISPR-associated endonuclease Cas1	

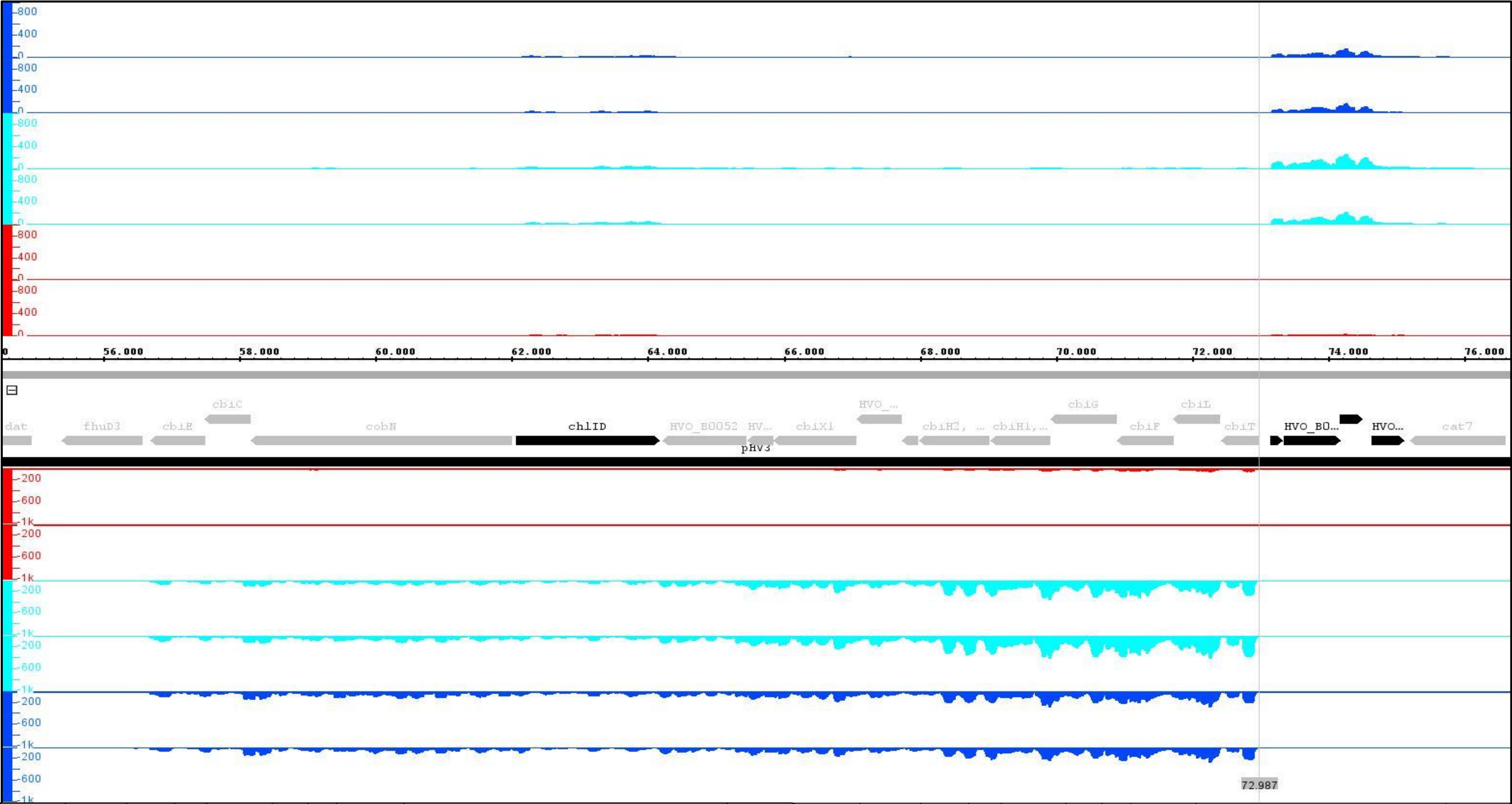

Name	Base mean	log2(FC)	P-adj	fold change	Fclass	AA	Gene name	Protein name	Condition
HVO_B0048	170	1.99	0.000	4.0	COM	270	cbiE	cobalt-precorrin-7 C5-methyltransferase	0.9M/26h vs Ctrl
HVO_B0049	72	1.98	0.005	3.9	COM	226	cbiC	cobalt-precorrin-8 methylmutase	
HVO_B0050	1298	2.47	0.000	5.6	COM	1284	cobN	ATP-dependent cobaltochelatase subunit CobN	
HVO_B0051	295	2.16	0.000	4.5	COM	706	chlID	ATP-dependent cobaltochelatase subunit ChlID	
HVO_B0052	448	2.84	0.000	7.2	GEN	412	0	PQQ repeat protein	
HVO_B0053	111	3.47	0.000	11.0	GEN	127	0	DUF3209 family protein	
HVO_B0054	696	3.02	0.000	8.1	COM	406	cbiX1	sirohydrochlorin cobaltochelatase	
HVO_B0055	246	2.86	0.001	7.3	CHY	225	0	conserved hypothetical protein	
HVO_B0056	31	2.29	0.004	4.9	MIS	84	0	probable ferredoxin (4Fe-4S)	
HVO_B0057	749	3.17	0.000	9.0	COM	344	cbiH2, cobJ2	cobalt-factor-III C17-methyltransferase	
HVO_B0058	556	2.91	0.000	7.5	COM	294	cbiH1, cobJ1	cobalt-factor-III C17-methyltransferase	
HVO_B0059	721	2.86	0.000	7.3	COM	326	cbiG	cobalt-precorrin-5A hydrolase	
HVO_B0060	775	2.44	0.000	5.4	COM	279	cbiF	cobalt-precorrin-4 C11-methyltransferase	
HVO_B0061	739	2.35	0.000	5.1	COM	235	cbiL	cobalt-factor-II C20-methyltransferase	
HVO_B0062	459	2.43	0.000	5.4	COM	184	cbiT	cobalt-precorrin-6B C15-methyltransferase (decarboxylating)	
HVO_B0063	103	3.52	0.000	11.5	GEN	61	0	CbtB family protein	
HVO_B0064	434	3.81	0.000	14.1	GEN	278	0	CbtA family protein	
HVO_B0065	232	3.51	0.000	11.4	GEN	113	0	thioredoxin domain protein	
HVO_B0066	96	2.54	0.000	5.8	REG	164	0	Lrp/AsnC family transcription regulator	

Name	Base mean	log2(FC)	P-adj	fold change	Fclass	AA	Gene name	Protein name	Condition
HVO_B0048	176	2.51	0.000	5.7	COM	270	cbiE	cobalt-precorrin-7 C5-methyltransferase	0.9M/68h vs Ctrl
HVO_B0049	79	2.61	0.000	6.1	COM	226	cbiC	cobalt-precorrin-8 methylmutase	
HVO_B0050	1407	3.05	0.000	8.3	COM	1284	cobN	ATP-dependent cobaltochelatase subunit CobN	
HVO_B0051	198	1.93	0.000	3.8	COM	706	chlID	ATP-dependent cobaltochelatase subunit ChlID	
HVO_B0052	390	3.05	0.000	8.3	GEN	412	0	PQQ repeat protein	
HVO_B0053	81	3.39	0.000	10.5	GEN	127	0	DUF3209 family protein	
HVO_B0054	510	2.95	0.000	7.7	COM	406	cbiX1	sirohydrochlorin cobaltochelatase	
HVO_B0055	168	2.68	0.001	6.4	CHY	225	0	conserved hypothetical protein	
HVO_B0056	20	2.01	0.034	4.0	MIS	84	0	probable ferredoxin (4Fe-4S)	
HVO_B0057	469	2.85	0.000	7.2	COM	344	cbiH2, cobJ2	cobalt-factor-III C17-methyltransferase	
HVO_B0058	360	2.64	0.000	6.2	COM	294	cbiH1, cobJ1	cobalt-factor-III C17-methyltransferase	
HVO_B0059	452	2.53	0.000	5.8	COM	326	cbiG	cobalt-precorrin-5A hydrolase	
HVO_B0060	495	2.13	0.000	4.4	COM	279	cbiF	cobalt-precorrin-4 C11-methyltransferase	
HVO_B0061	537	2.26	0.000	4.8	COM	235	cbiL	cobalt-factor-II C20-methyltransferase	
HVO_B0062	307	2.20	0.001	4.6	COM	184	cbiT	cobalt-precorrin-6B C15-methyltransferase (deca	
HVO_B0063	49	2.78	0.000	6.8	GEN	61	0	CbtB family protein	
HVO_B0064	252	3.38	0.000	10.4	GEN	278	0	CbtA family protein	
HVO_B0065	137	3.09	0.000	8.5	GEN	113	0	thioredoxin domain protein	
HVO_B0066	50	1.87	0.002	3.7	REG	164	0	Lrp/AsnC family transcription regulator	

Supplementary Figure S4: Overview over upregulated clusters HVO_A0205-A0211 (CRISPR/CAS) and B0048-B0066 (Cobalt associated genes). Shown are visualized read counts for the indicated genes in the IGB. The two replicates for the control condition are shown in red, for 26h low salt in teal, the 68 h low salt in blue and the gene annotation in black (forward strand) and grey (reverse strand). RNA-Seq data as obtained via the DESeq2 tool from the galaxy platform for the indicated genes and conditions is shown in the table below.
