# Supplementary material for: Studying the long-term adaptation of Haloferax volcanii to low salt conditions: transcriptomic and genetic analyses
Source: Front Microbiol. 2026 Jan 15;16:1697018. doi: 10.3389/fmicb.2025.1697018 (PMC12852389; doi:10.3389/fmicb.2025.1697018)
Supplement: Supplementary file 5 [file Data_Sheet_5.pdf]

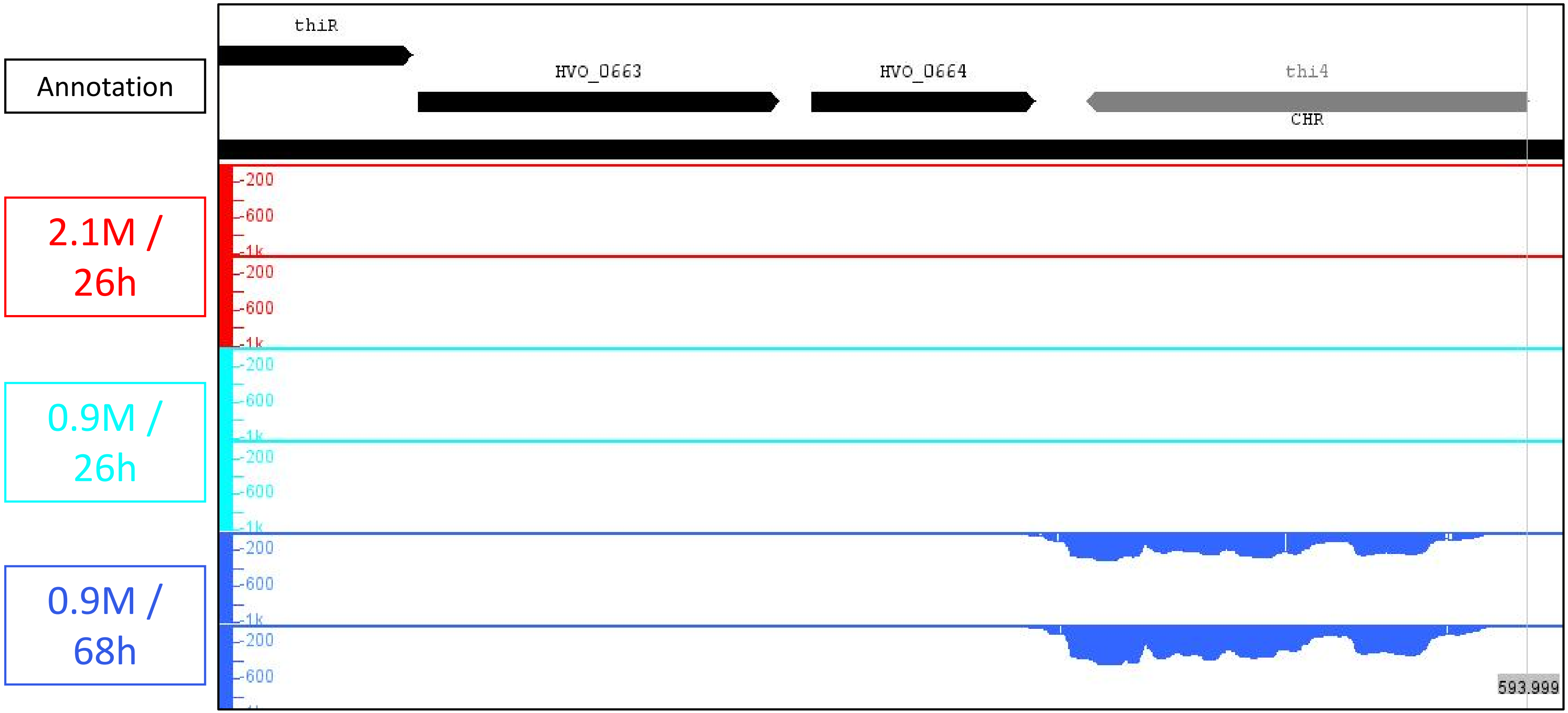

Name	Condition	Base mean	log2(FC)	P-adj	fold change	Fclass	AA	Gene name	Protein Name
HVO_0665	**0.9M/26h vs Ctrl**	55	-2.23	0.026	0.213	COM	307	thi4	adenosine diphosphate thiazole synthase, cysteine-dependent
	**0.9M/68h vs Ctrl**	1098	4.95	0.000	30.93				
	**0.9M/68h vs 0.9M/26h**	2063	7.16	0.000	143.19				

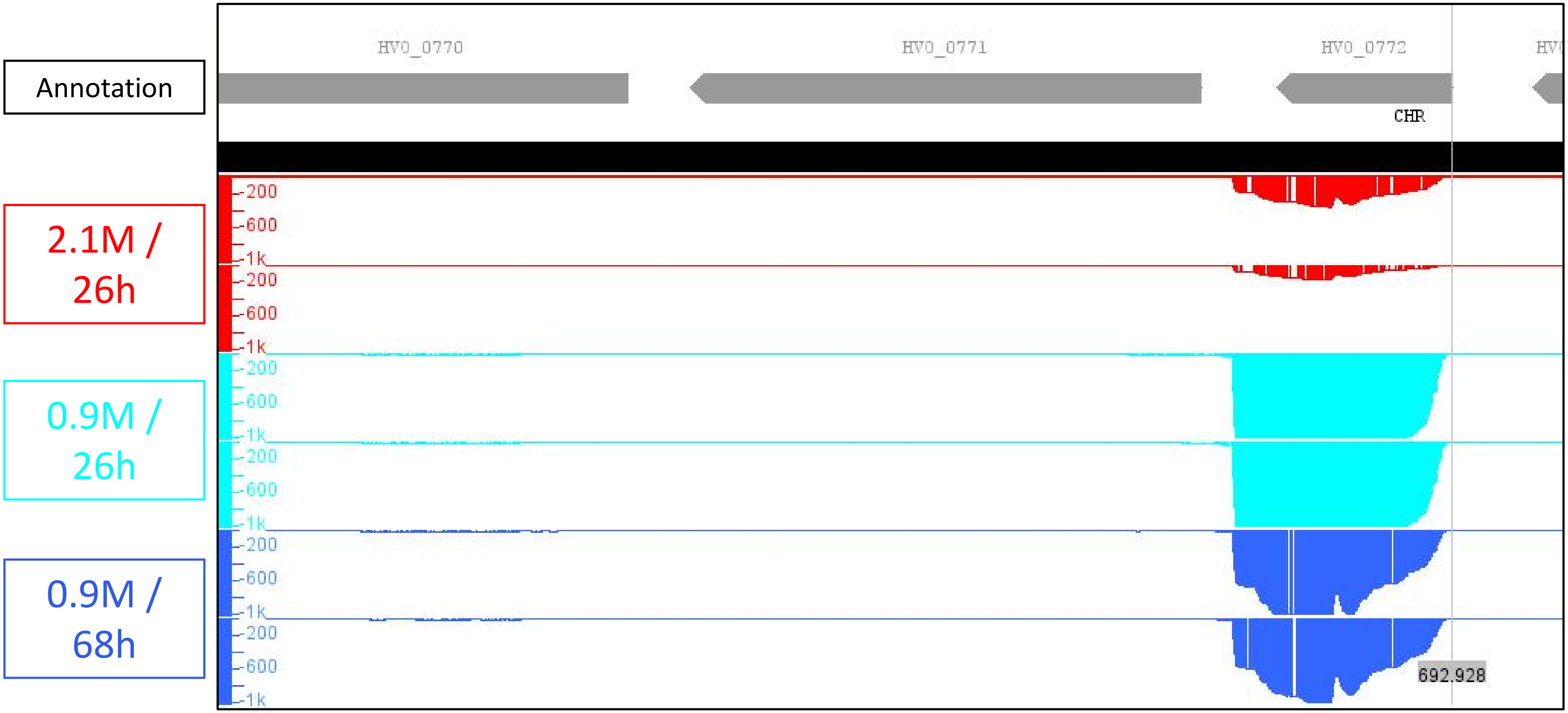

Name	Condition	Base mean	log2(FC)	P-adj	fold change	Fclass	AA	Gene name	Protein Name
HVO_0772	0.9M/26h vs Ctrl	3009	2.06	0.000	4.17	REG	90		NP_1176A family transcription regulator
	0.9M/68h vs Ctrl	1614	1.39	0.003	2.62				
	0.9M/68h vs 0.9M/26h				not				

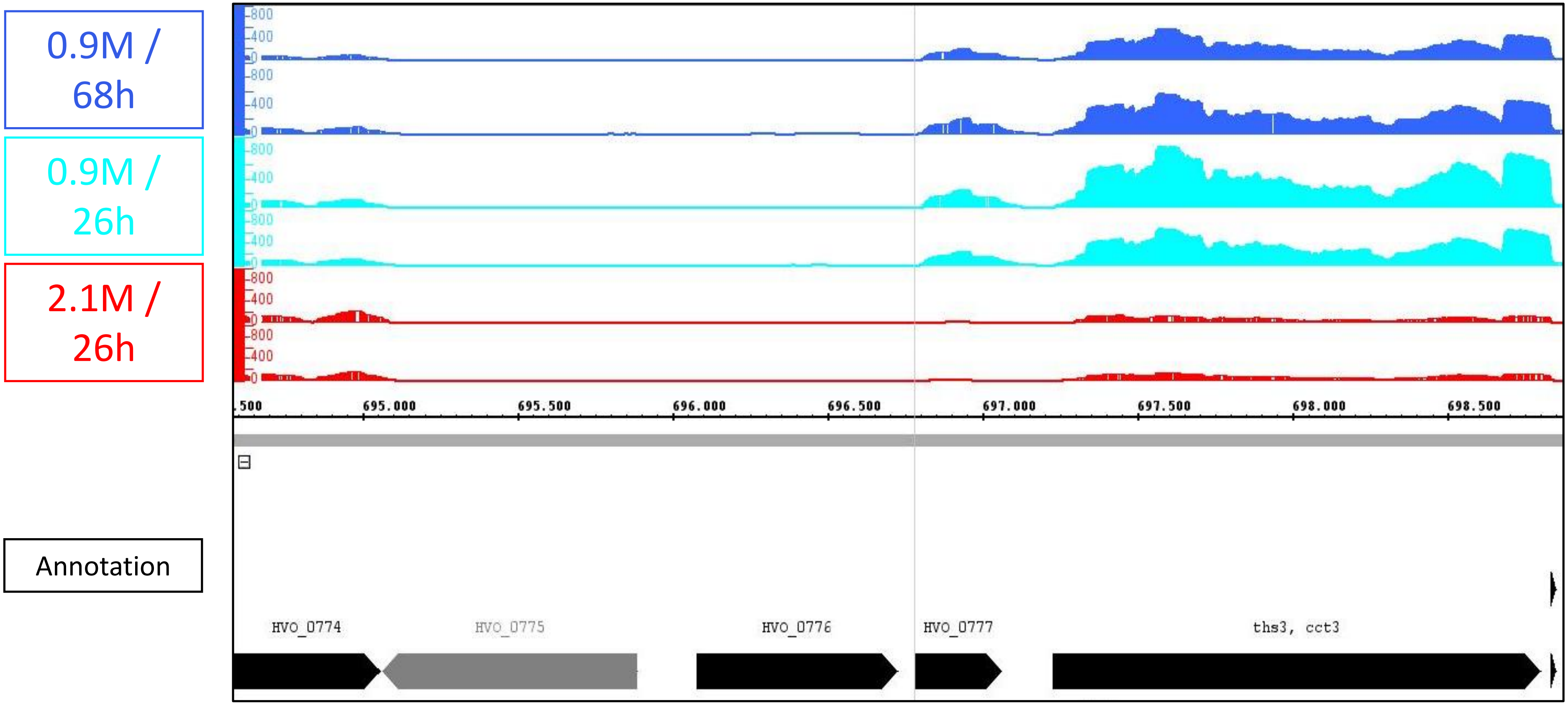

Name	Condition	Base mean	log2(FC)	P-adj	fold change	Fclass	AA	Gene name	Protein Name
**HVO_0777**	**0.9M/26h vs Ctrl**	365	3.35	0.000	10.17	GEN	93		HalOD1 domain protein
	**0.9M/68h vs Ctrl**	280	3.34	0.000	10.15				
	**0.9M/68h vs 0.9M/26h**				not				

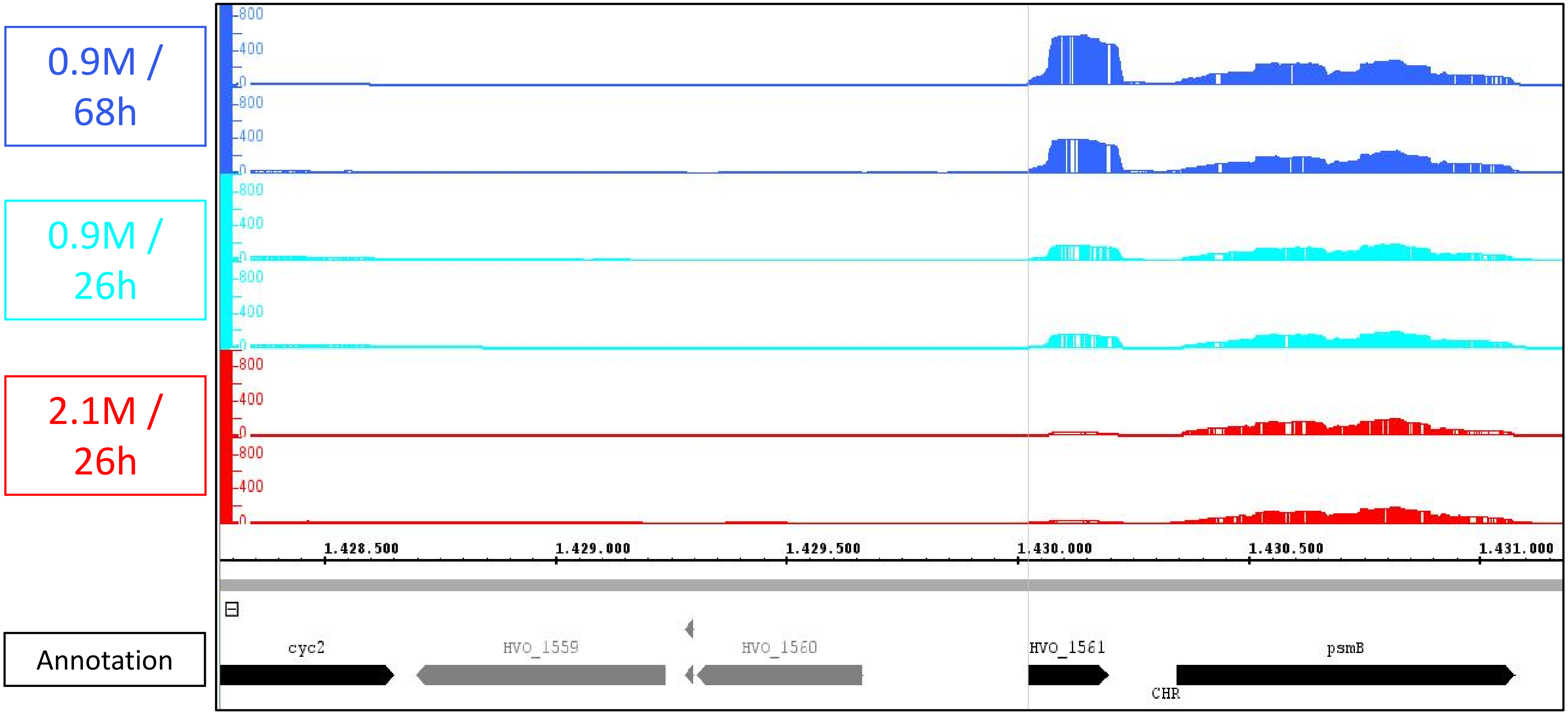

Name	Condition	Base mean	log2(FC)	P-adj	fold change	Fclass	AA	Gene name	Protein Name
**HVO_1561**	**0.9M/26h vs Ctrl**	216	1.98	0.000	3.95	CHY	57		conserved hypothetical protein
	**0.9M/68h vs Ctrl**	511	3.83	0.000	14.24				
	**0.9M/68h vs 0.9M/26h**	1178	1.82	0.000	3.54				

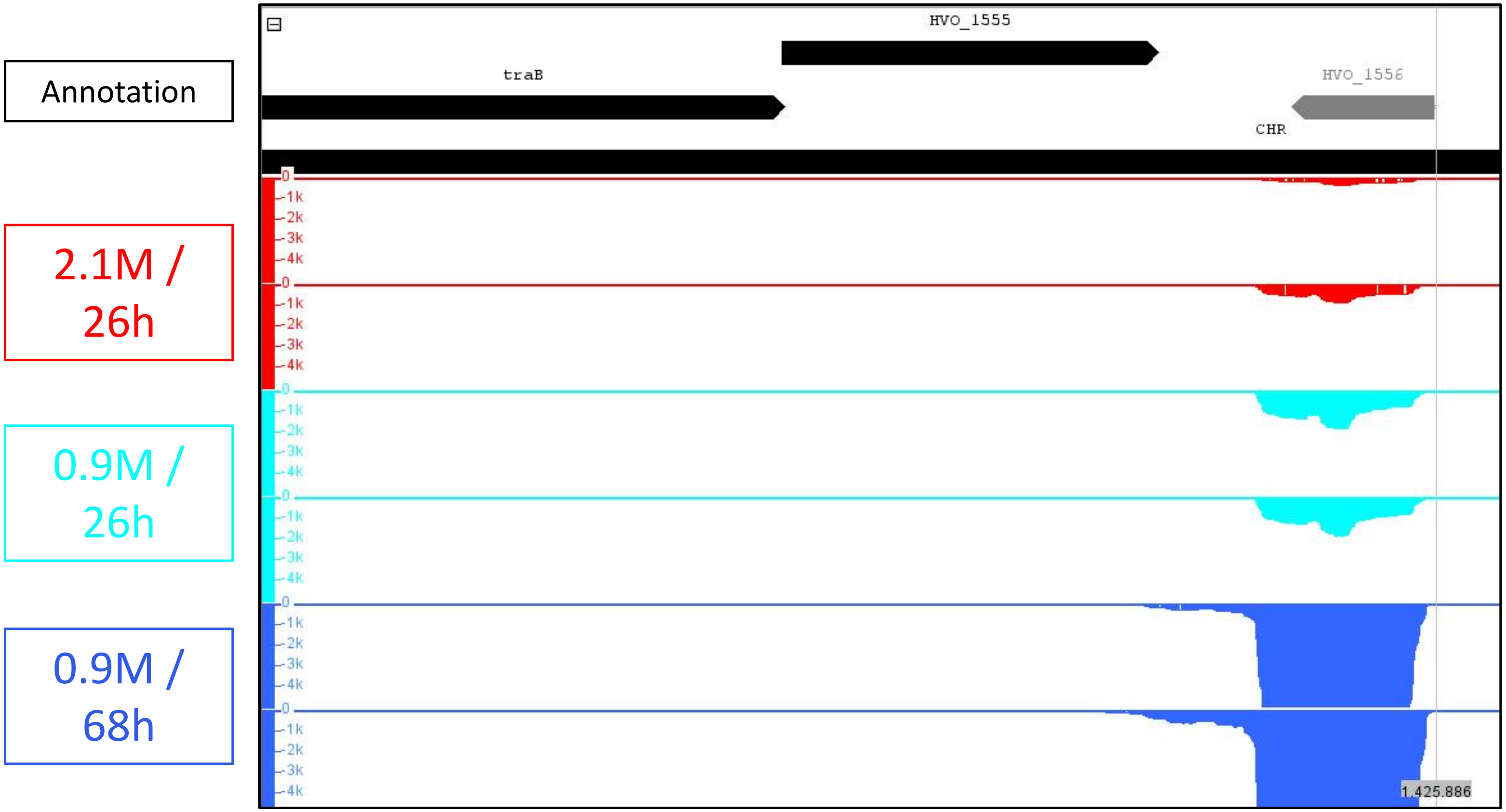

Name	Condition	Base mean	log2(FC)	P-adj	fold change	Fclass	AA	Gene name	Protein Name
HVO_1556	0.9M/26h vs Ctrl				not	CHY	78		conserved hypothetical protein
	0.9M/68h vs Ctrl	17537	3.99	0.000	15.92				
	0.9M/68h vs 0.9M/26h	34243	3.68	0.000	12.79				

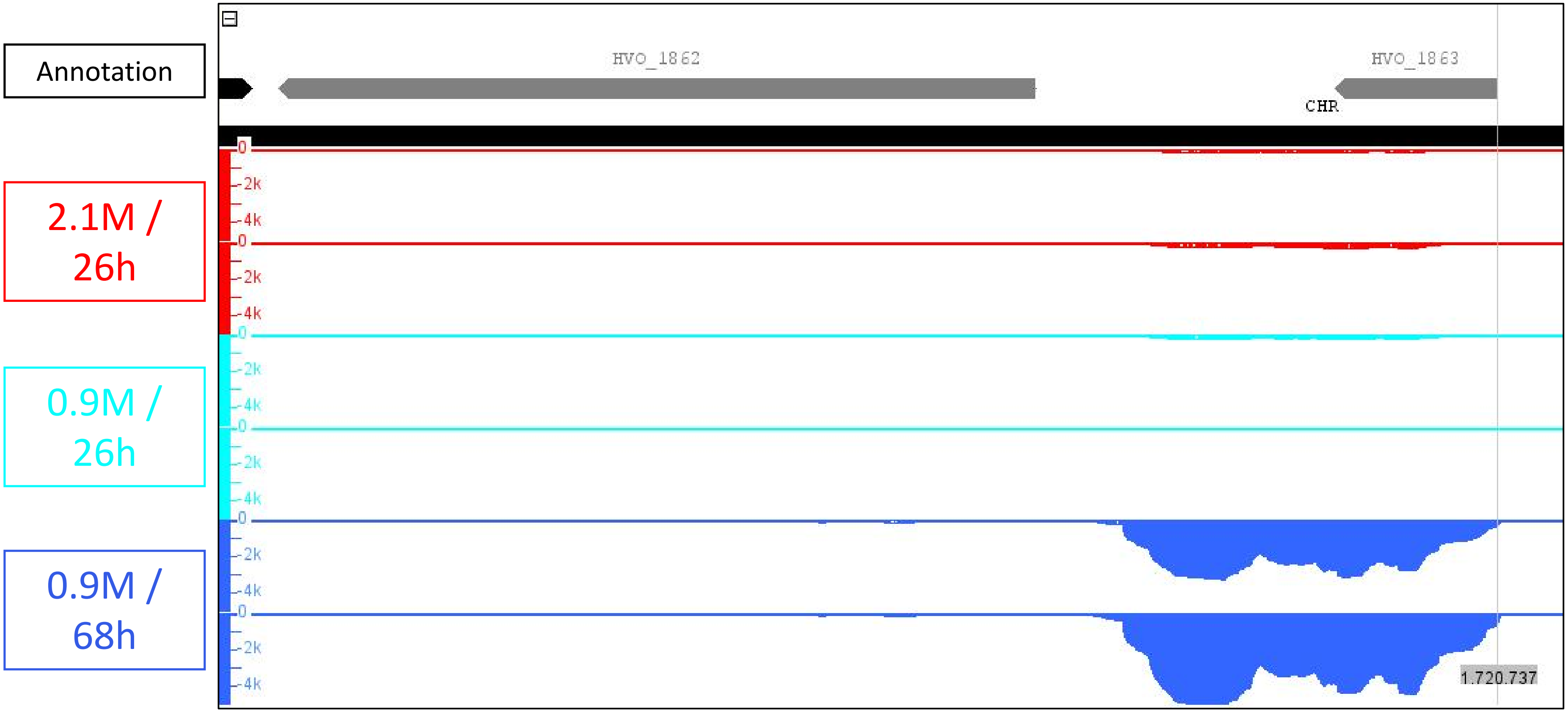

Name	Condition	Base mean	log2(FC)	P-adj	fold change	Fclass	AA	Gene name	Protein Name
HVO_1863	0.9M/26h vs Ctrl	1069	-1.72	0.018	0.30	CHY	81		conserved hypothetical protein
	0.9M/68h vs Ctrl	6050	3.10	0.000	8.57				
	0.9M/68h vs 0.9M/26h	10817	4.78	0.000	27.56				

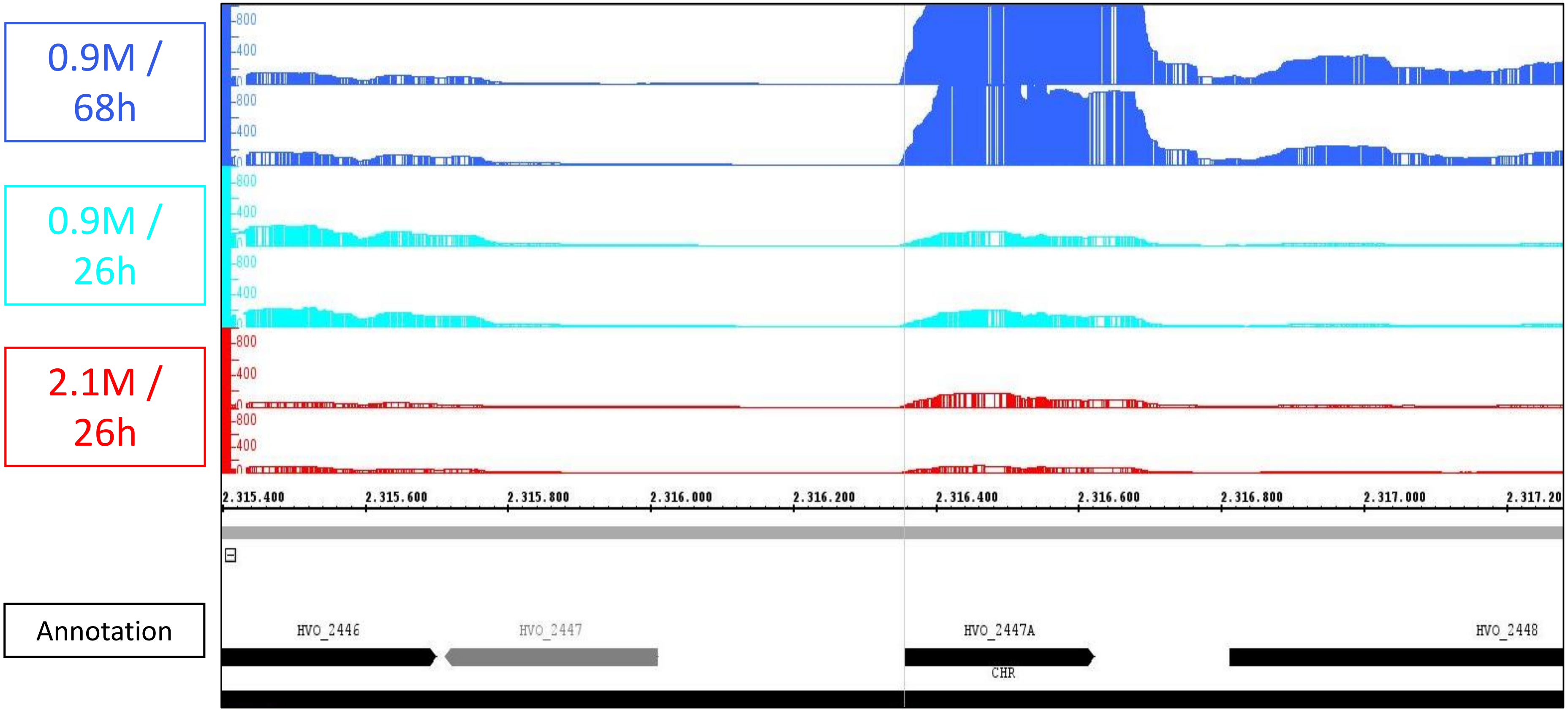

Name	Condition	Base mean	log2(FC)	P-adj	fold change	Fclass	AA	Gene name	Protein Name
HVO_2447A	0.9M/26h vs Ctrl				not	CHY	88		conserved hypothetical protein
	0.9M/68h vs Ctrl	3054	3.34	0.000	10.13				
	0.9M/68h vs 0.9M/26h	5845	3.44	0.000	10.88				

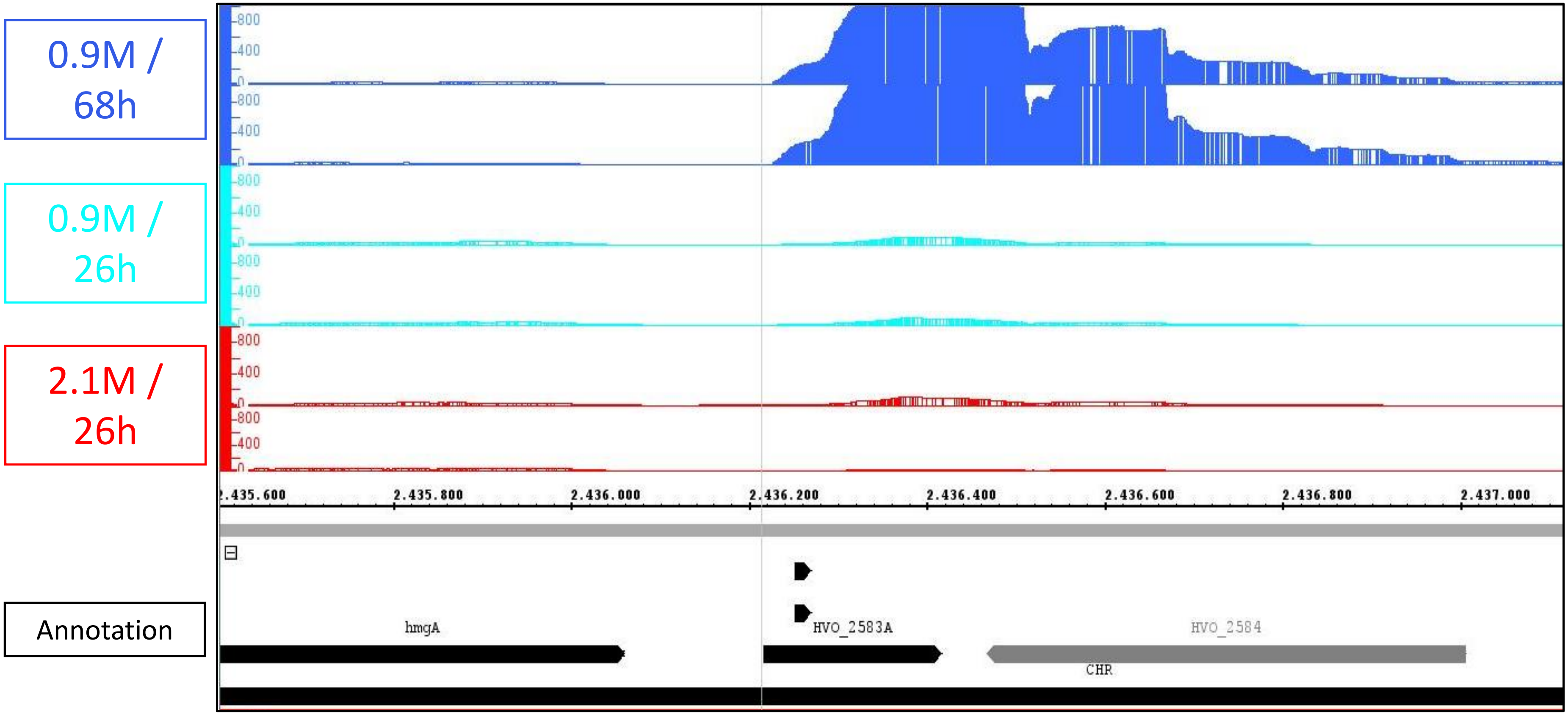

Name	Condition	Base mean	log2(FC)	P-adj	fold change	Fclass	AA	Gene name	Protein Name
HVO_2583A	0.9M/26h vs Ctrl				not	CHY	66		conserved hypothetical protein
	0.9M/68h vs Ctrl	3377	5.04	0.000	32.84				
	0.9M/68h vs 0.9M/26h	6493	5.22	0.000	37.23				

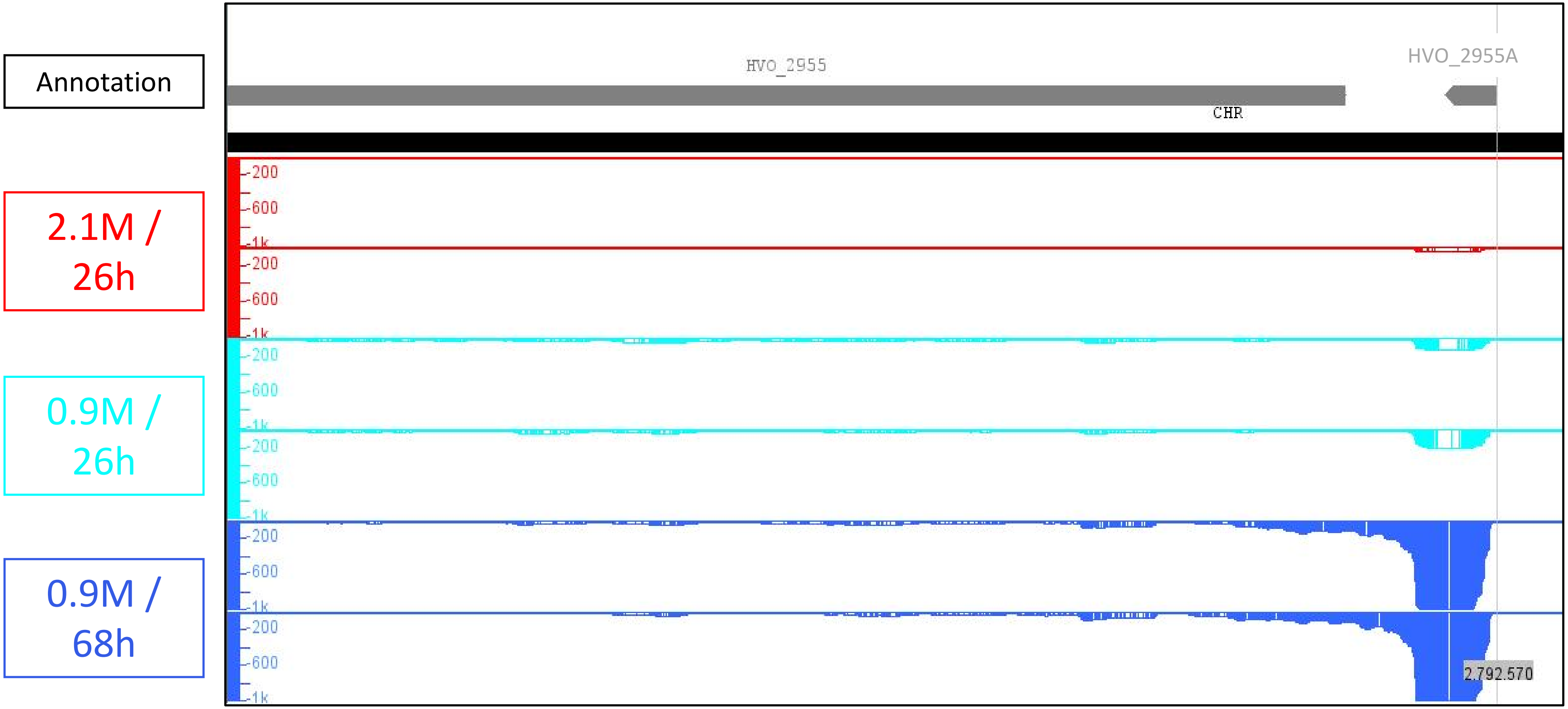

Name	Condition	Base mean	log2(FC)	P-adj	fold change	Fclass	AA	Gene name	Protein Name
HVO_2955A	0.9M/26h vs Ctrl				not	CHY	35		conserved hypothetical protein
	0.9M/68h vs Ctrl	936	4.38	0.000	20.84				
	0.9M/68h vs 0.9M/26h	1909	3.20	0.000	9.20				

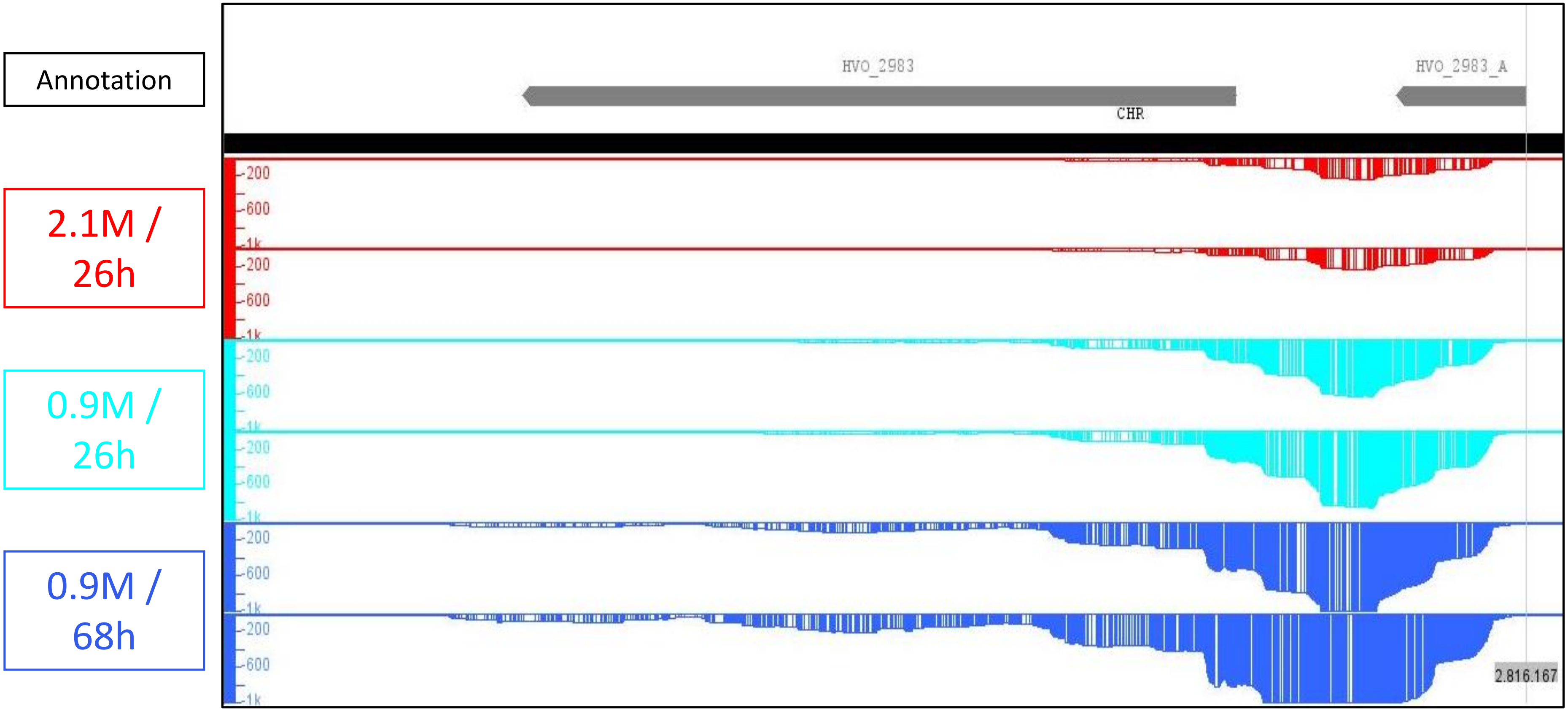

Name	Condition	Base mean	log2(FC)	P-adj	fold change	Fclass	AA	Gene name	Protein Name
**HVO_2983_A**	**0.9M/26h vs Ctrl**				not	CHY	38		conserved hypothetical protein
	**0.9M/68h vs Ctrl**	861	1.42	0.001	2.68				
	**0.9M/68h vs 0.9M/26h**	1809	1.00	0.000	2.01				

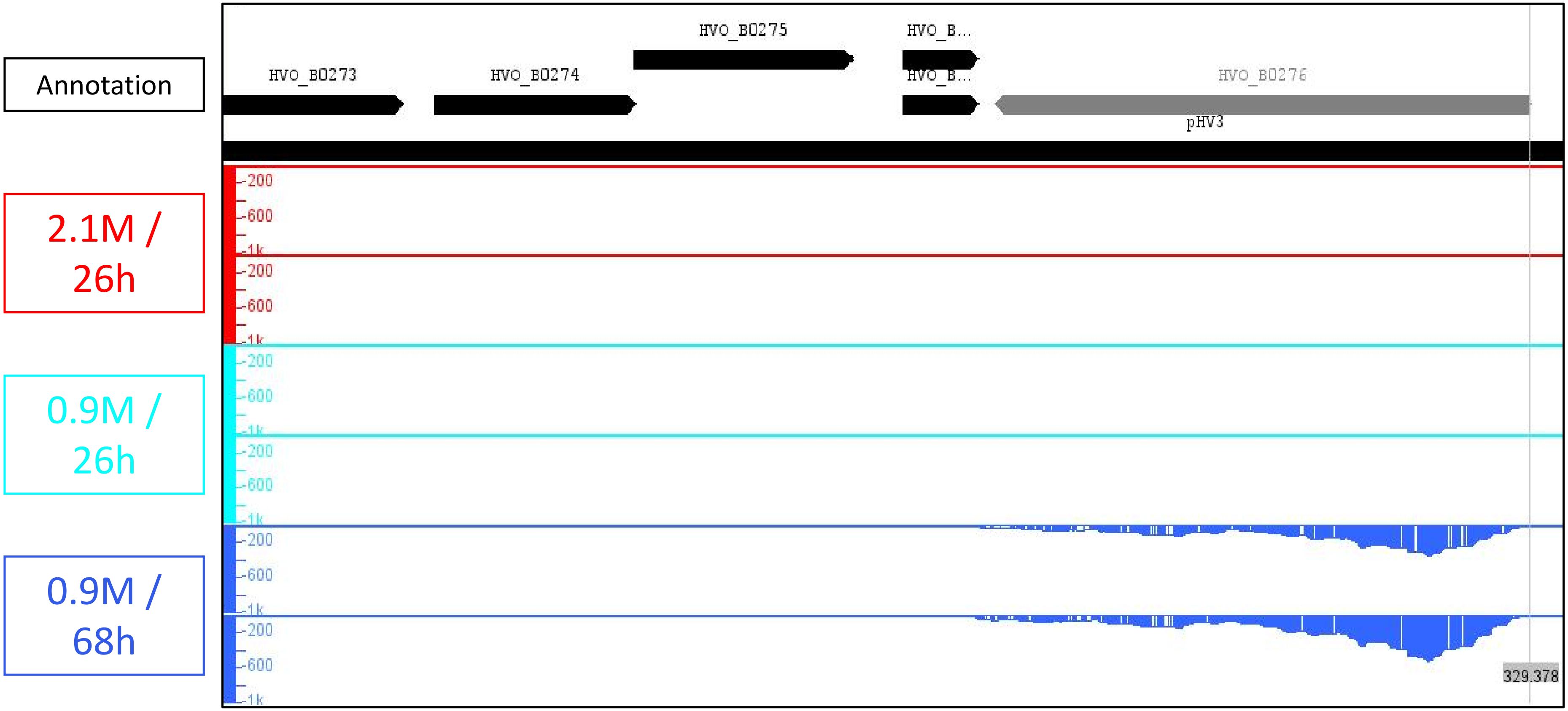

Name	Condition	Base mean	log2(FC)	P-adj	fold change	Fclass	AA	Gene name	Protein Name
HVO_B0276	0.9M/26h vs Ctrl				not	TP	323		DMT superfamily transport protein
	0.9M/68h vs Ctrl	758	5.40	0.000	42.16				
	0.9M/68h vs 0.9M/26h	1463	5.23	0.000	37.49				

Supplementary Figure S5: RNA-Seq data for selected genes chosen for further characterization for their role to low salt survival and adaption. Shown are visualized read counts for the indicated genes. The two replicates for the control condition are shown in red, for 26h low salt in teal, the 68 h low salt in blue and the gene annotation in black (forward strand) and grey (reverse strand). RNA-Seq data as obtained via the DESeq2 tool from the galaxy platform for the indicated genes and conditions is shown in the table below.
