# Supplementary material for: Studying the long-term adaptation of Haloferax volcanii to low salt conditions: transcriptomic and genetic analyses
Source: Front Microbiol. 2026 Jan 15;16:1697018. doi: 10.3389/fmicb.2025.1697018 (PMC12852389; doi:10.3389/fmicb.2025.1697018)
Supplement: Supplementary file 6 [file Data_Sheet_6.pdf]

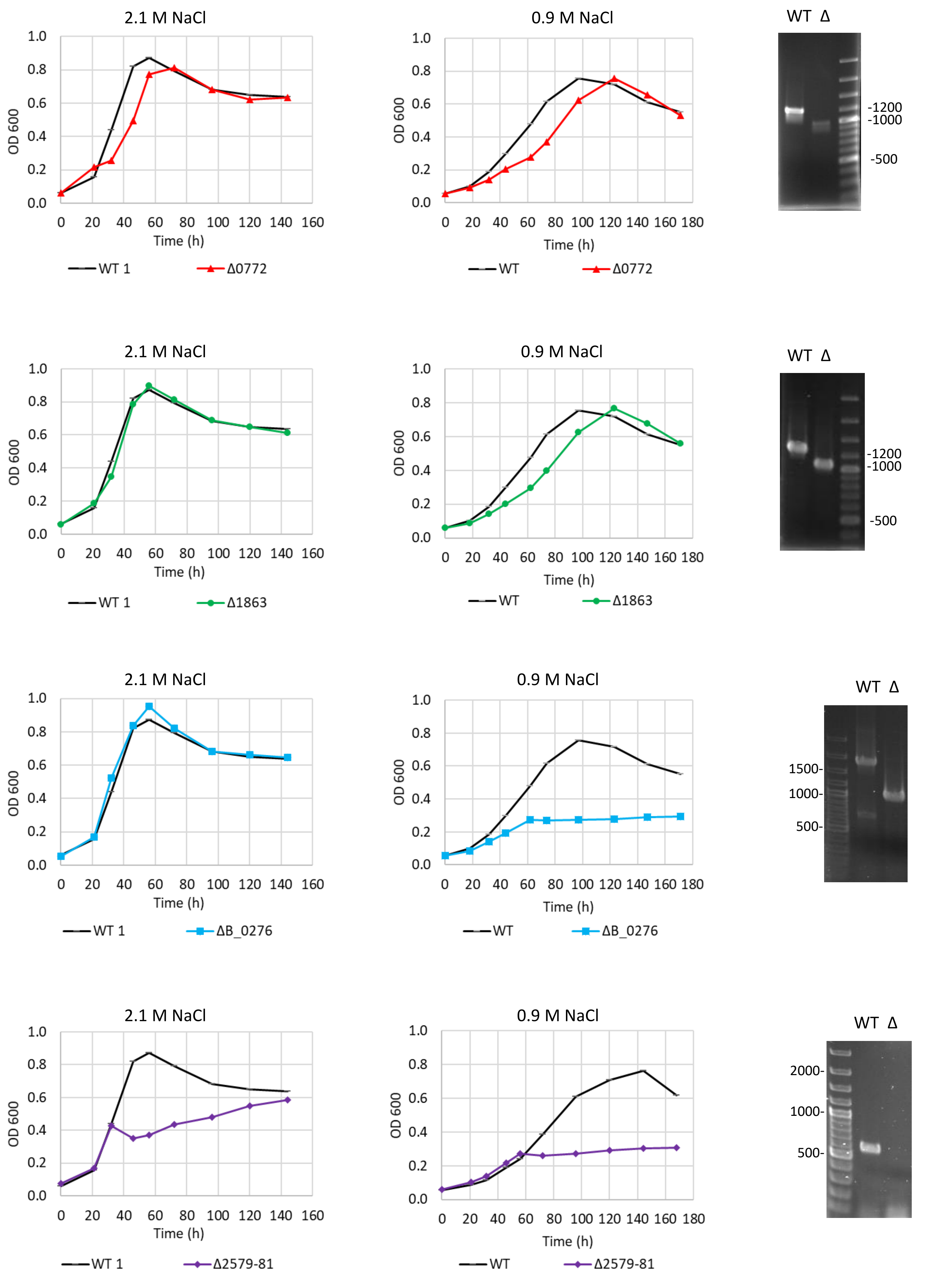

2.1 M NaCl
0.9 M NaCl
WT Δ
-1200
-1000
-500
OD 600
Time (h)
WT 1
Δ0772
WT
Δ0772
2.1 M NaCl
0.9 M NaCl
WT Δ
-1200
-1000
-500
WT 1
Δ1863
WT
Δ1863
2.1 M NaCl
0.9 M NaCl
WT Δ
1500-
1000-
500-
WT 1
ΔB_0276
WT
ΔB_0276
2.1 M NaCl
0.9 M NaCl
WT Δ
2000-
1000-
500-
WT 1
Δ2579-81
WT
Δ2579-81

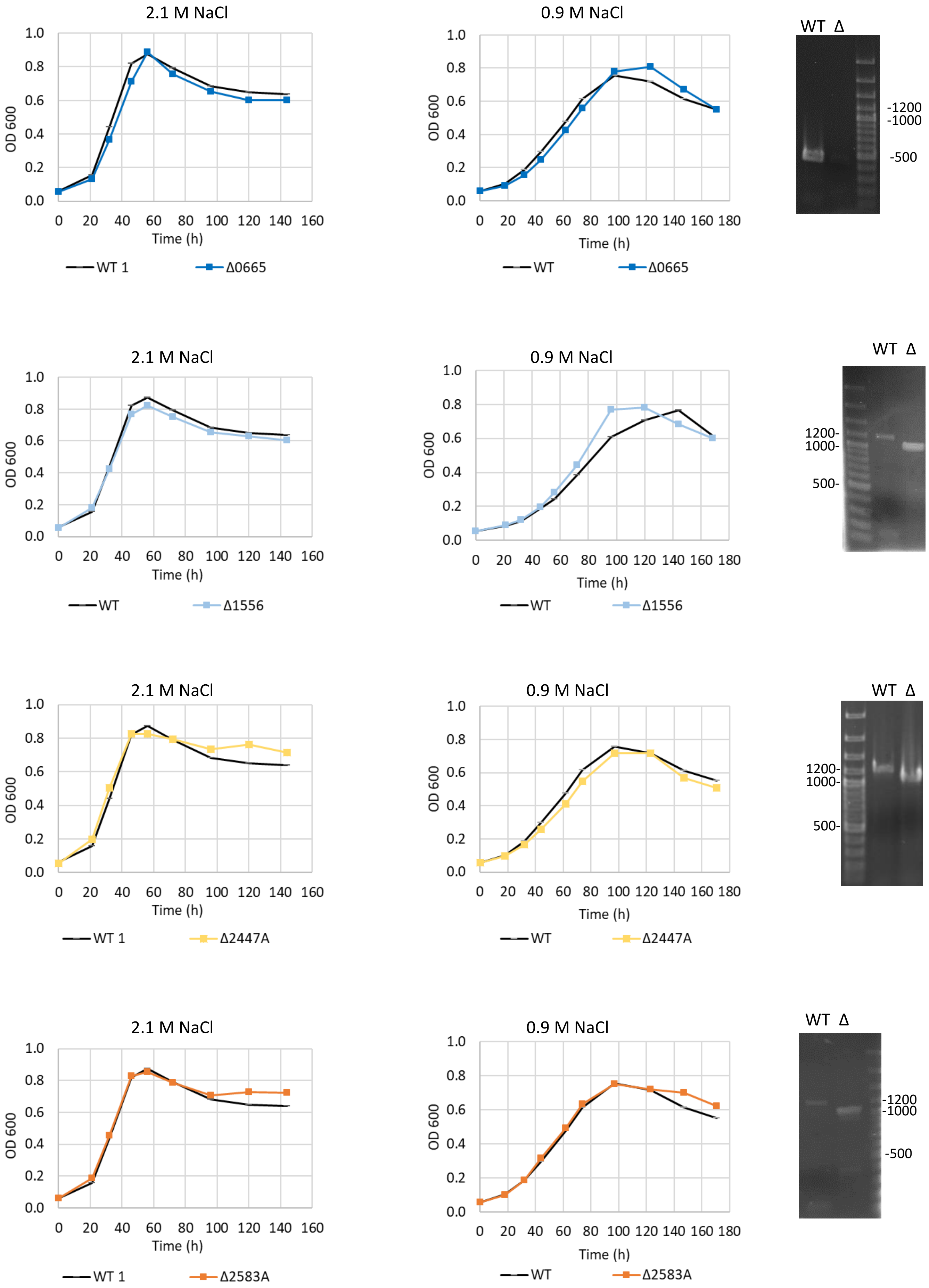
2.1 M NaCl
0.9 M NaCl
OD 600
Time (h)
WT 1
Δ0665
WT
WT Δ
-1200
-1000
-500
Δ1556
1200-
1000-
500-
Δ2447A
Δ2583A

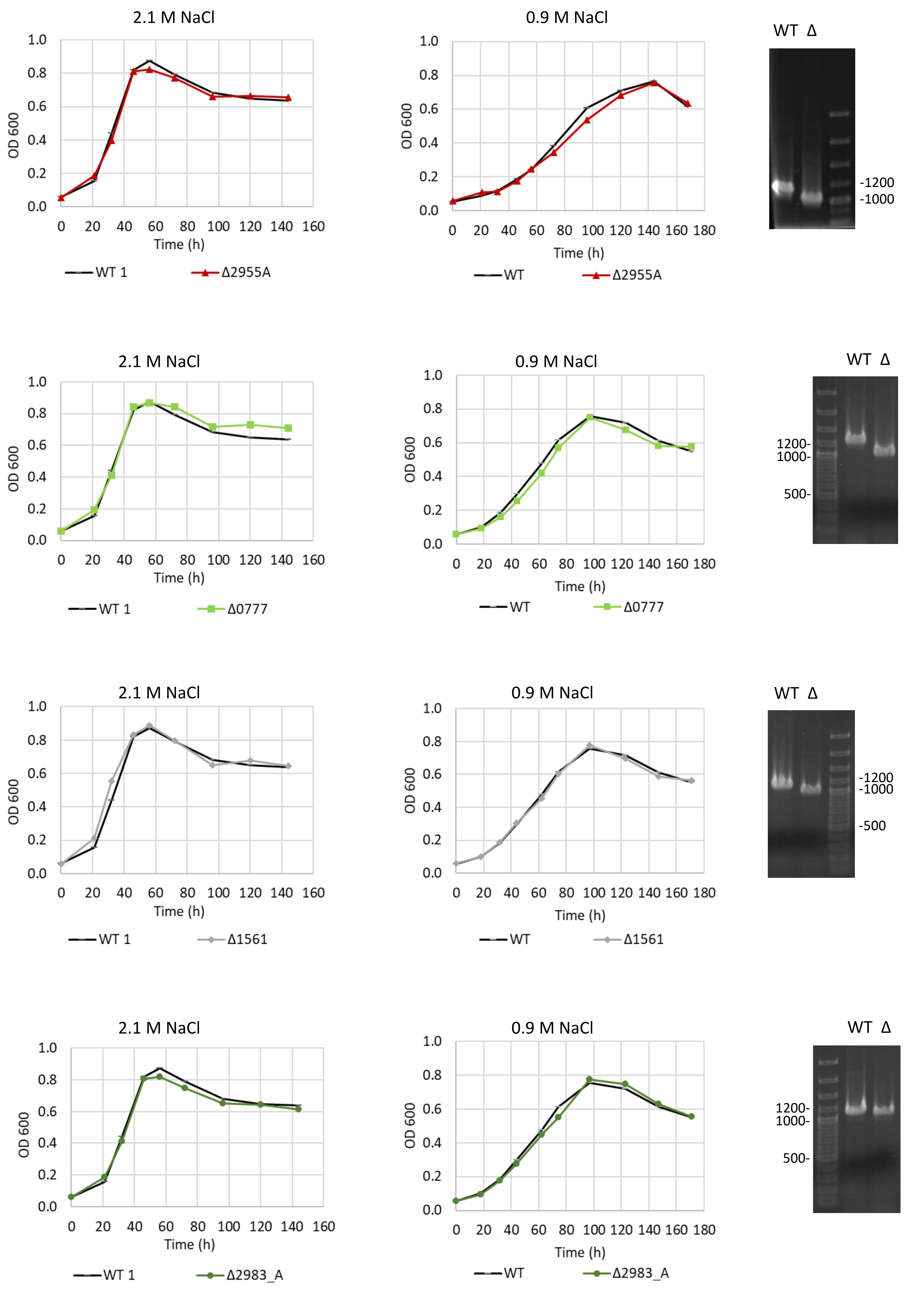
2.1 M NaCl
0.9 M NaCl
OD 600
Time (h)
WT 1
Δ2955A
WT
Δ2955A
WT Δ
-1200
-1000
2.1 M NaCl
0.9 M NaCl
OD 600
Time (h)
WT 1
Δ0777
WT
Δ0777
WT Δ
1200-
1000-
500-
2.1 M NaCl
0.9 M NaCl
OD 600
Time (h)
WT 1
Δ1561
WT
Δ1561
WT Δ
-1200
-1000
-500
2.1 M NaCl
0.9 M NaCl
OD 600
Time (h)
WT 1
Δ2983_A
WT
Δ2983_A
WT Δ
1200-
1000-
500-

Primer P1 & P4

Name	Size (nt) deletion fragment	Size (nt) wild-type fragment	Size (nt) deletion	Size (nt) gene(s)
**HVO_0665**	1026	1872	846	924
**HVO_0772**	1003	1225	222	273
**HVO_0777**	1037	1253	216	282
**HVO_1556**	1055	1217	162	237
**HVO_1561**	1022	1136	114	174
**HVO_1863**	1042	1246	204	246
**HVO_2447A**	1051	1243	192	267
**HVO_2583A**	1048	1201	153	201
**HVO_2955A**	1019	1103	84	108
**HVO_2983_A**	1020	1104	84	117
**HVO_B0276**	1026	1893	867	972
**HVO_2579-2581**	1110	4548	3438	3561

Analysis Primer

Name	Size (nt) deletion fragment	Size (nt) wild-type fragment	Size (nt) deletion	Size (nt) gene(s)
**HVO_0665**	not present	449	846	924
**HVO_2579-2581**	not present	607	3438	3561

Supplementary Figure S6: H26 WT cells and indicated in-frame deletion mutants grown in synthetic glucose medium with 2.1 M (left panel) and 0.9 M (middle panel) NaCl in test glasses and a total volume of 12 ml inoculated from a preculture (2.1 M NaCl; glucose; start OD600 of 0.05). 150µl from each culture were taken and OD600 measured frequently. Curves are representative for at least three biological replicates. Multicycle PCR was used to frequently check for the deletion of the respective gene (right panel) using the primers P1 & P4. For HVO_0665 and the cluster HVO_2579-81 the analysis primers were used instead. Expected fragment sizes for the PCR products can be found in the table above.
