# Supplementary material for: Studying the long-term adaptation of Haloferax volcanii to low salt conditions: transcriptomic and genetic analyses
Source: Front Microbiol. 2026 Jan 15;16:1697018. doi: 10.3389/fmicb.2025.1697018 (PMC12852389; doi:10.3389/fmicb.2025.1697018)
Supplement: Supplementary file 7 [file Data_Sheet_7.pdf]

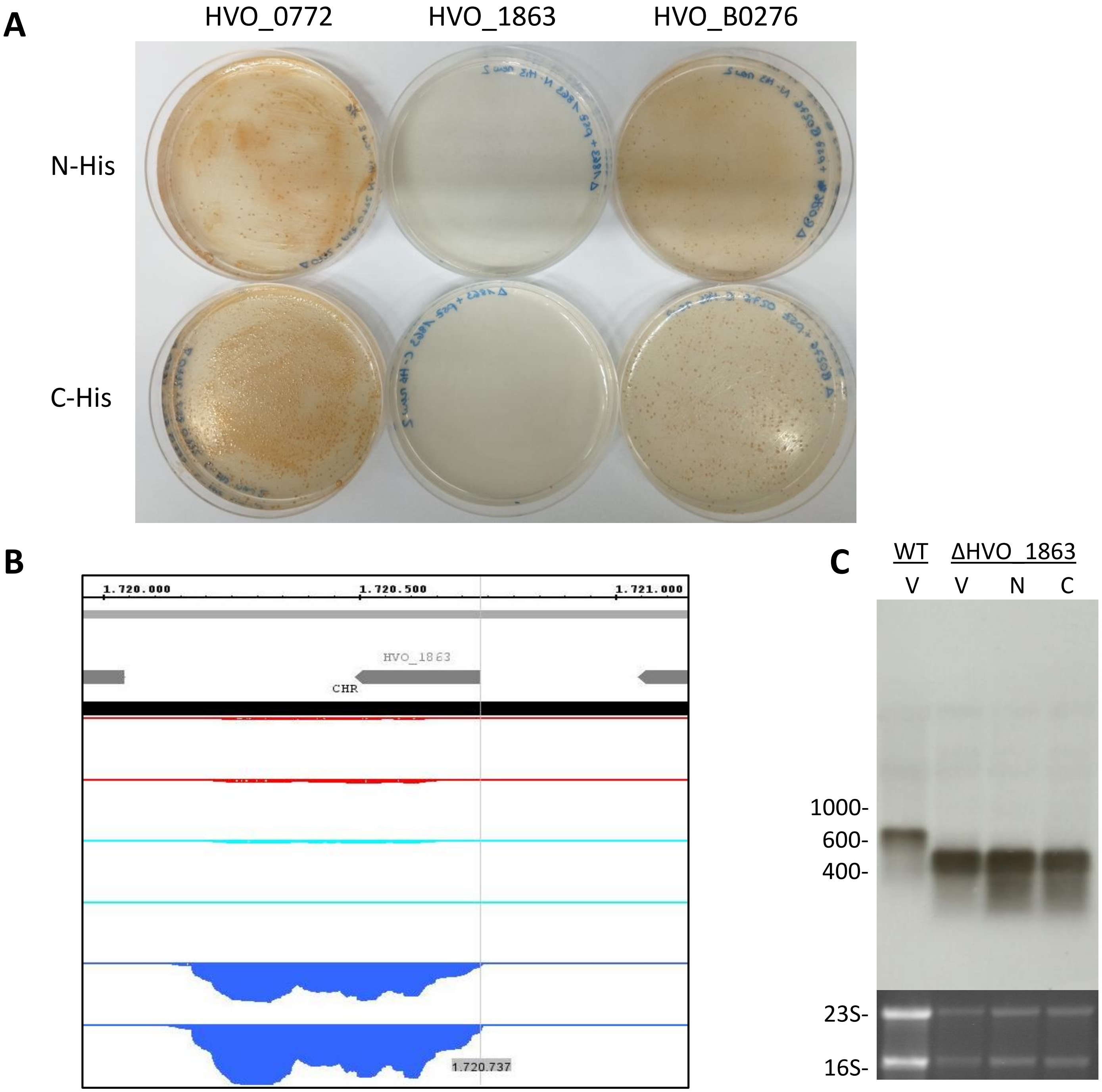


**Supplementary Figure S7:** Overexpression of HVO_1863 was not possible. A) Petri dishes showing Haloferax cells after transformation with expression vectors carrying the ORF of the indicated genes modified with N- or C-terminal Hexa-Histidin tag. B) Screenshot from the IGB of the HVO_1863 locus showing RNA-Seq data and visualized read counts for two replicates for the control condition (red), the 26 h low salt condition (teal), and the 68 h low salt condition (blue). Gene annotation is shown in grey (reverse strand). The ORF of HVO_1863 counts 246 base pairs. C) Northern blots for the HVO_1863 gene. Cells were harvested under control condition with Novobiocin in exponential growth phase and RNA was extracted as described. H26 wild type cells and the HVO_1863 deletion mutant transformed with the empty vector (V), vectors carrying the N-terminal (N) and the C-terminal (C) His-tagged variant of the HVO_1863 gene were used. Base pair sizes are indicated on the left.
