# Supplementary material for: Studying the long-term adaptation of Haloferax volcanii to low salt conditions: transcriptomic and genetic analyses
Source: Front Microbiol. 2026 Jan 15;16:1697018. doi: 10.3389/fmicb.2025.1697018 (PMC12852389; doi:10.3389/fmicb.2025.1697018)
Supplement: Supplementary file 8 [file Data_Sheet_8.pdf]

HVO_0665

adenosine diphosphate thiazole synthase, cysteine-dependent

A

FUNCTION

Function Class:	COM	(coenzyme metabolism)
Superclass:	MET	(metabolism (AA, CHM, CIM, COM, EM, LIP, NUM))

ORF PROPERTIES

Length(nucleic bases):	924	Start Codon:	ATG	Stop Codon:	TGA
%GC:	70%	CAI:	0.85		

PROTEIN PROPERTIES

Length(amino acids):	307	MolWeight(Da):	32,404
pI value:	4.4	GRAVY index:	-0.15
TM domains:	0	signal sequence cleavage site:	No
TAT export signal prediction:	No	RR..LAGC motif:	No

B

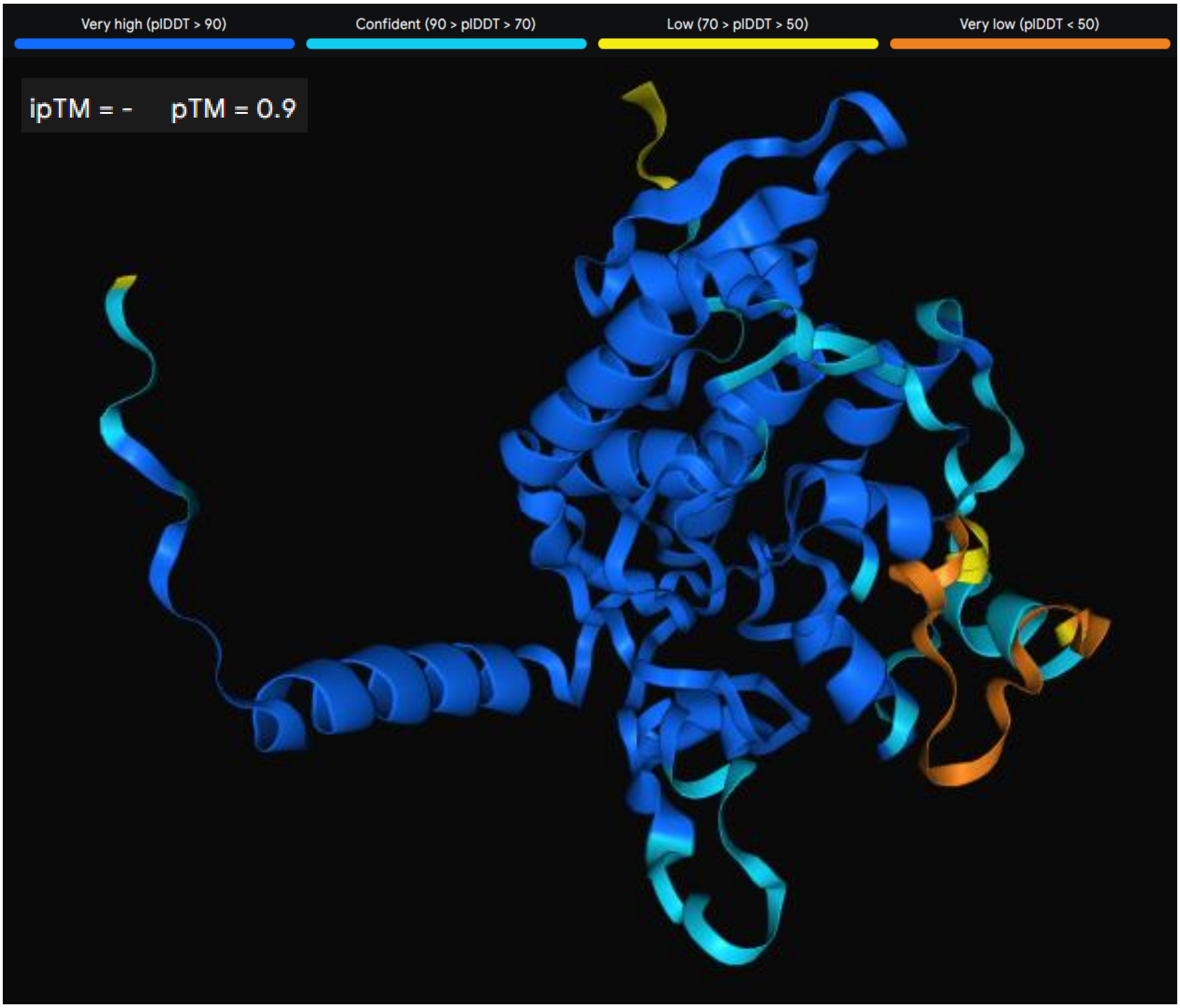

HVO_0772

NP_1176A family transcription regulator

A

FUNCTION

Function Class:	REG	(gene regulation)
Superclass:	ENV	(environmental information processing (REG, SIG))

ORF PROPERTIES

Length(nucleic bases):	273	Start Codon:	ATG	Stop Codon:	TAA
%GC:	64%	CAI:	0.7		

PROTEIN PROPERTIES

Length(amino acids):	90	MolWeight(Da):	10,198
pI value:	4.7	GRAVY index:	-0.6
TM domains:	0	signal sequence cleavage site:	No
TAT export signal prediction:	No	RR..LAGC motif:	No

B

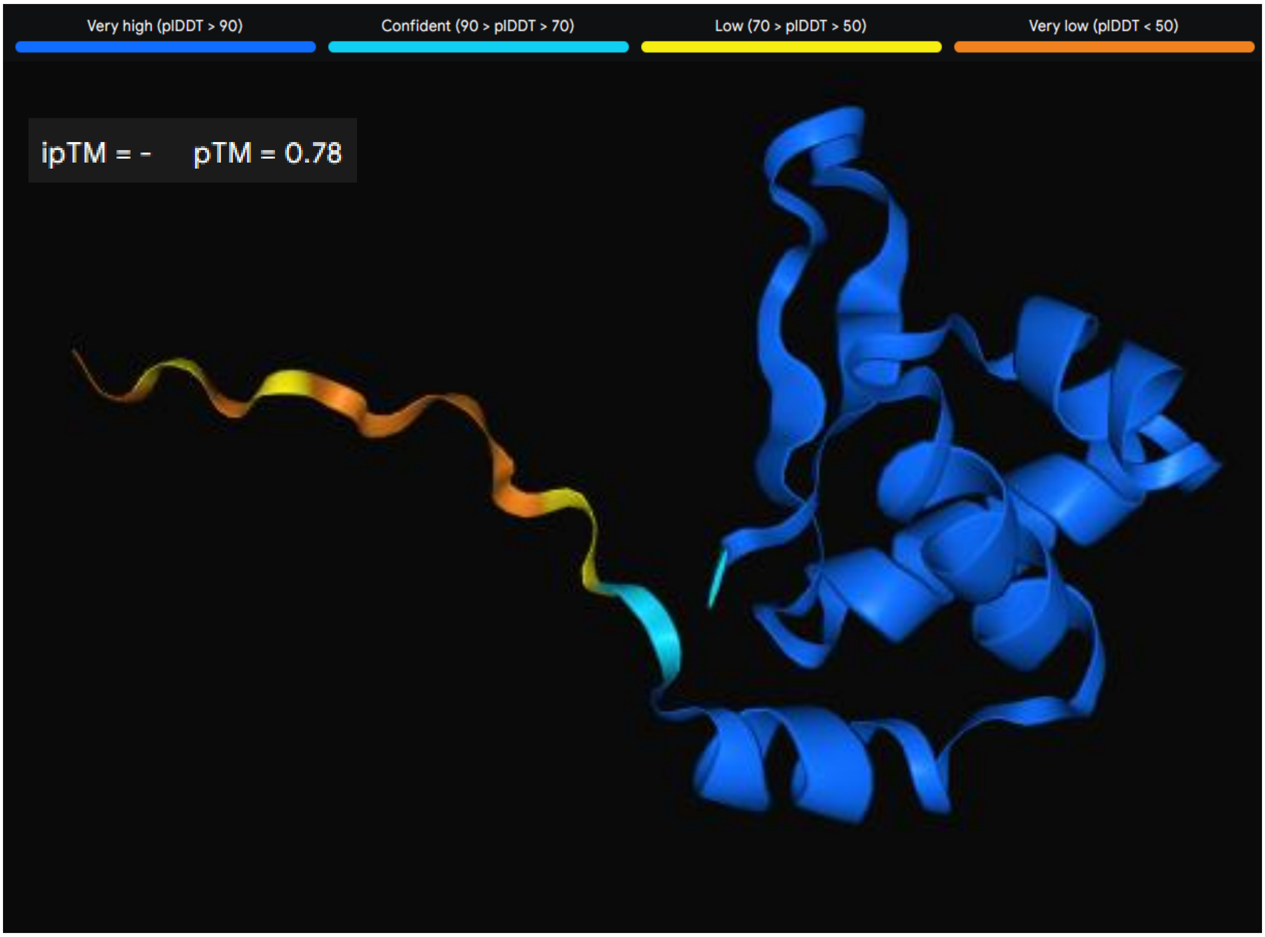

HVO_0777

HalOD1 domain protein

A

FUNCTION

Function Class:	GEN	(general enzymatic function)
Superclass:	MIS	(miscellaneous (GEN, ISH, MIS))

ORF PROPERTIES

Length(nucleic bases):	282	Start Codon:	ATG	Stop Codon:	TAG
%GC:	66%	CAI:	0.63		

PROTEIN PROPERTIES

Length(amino acids):	93	MolWeight(Da):	9,948
pI value:	4.1	GRAVY index:	0.03
TM domains:	0	signal sequence cleavage site:	No
TAT export signal prediction:	No	RR..LAGC motif:	No

B

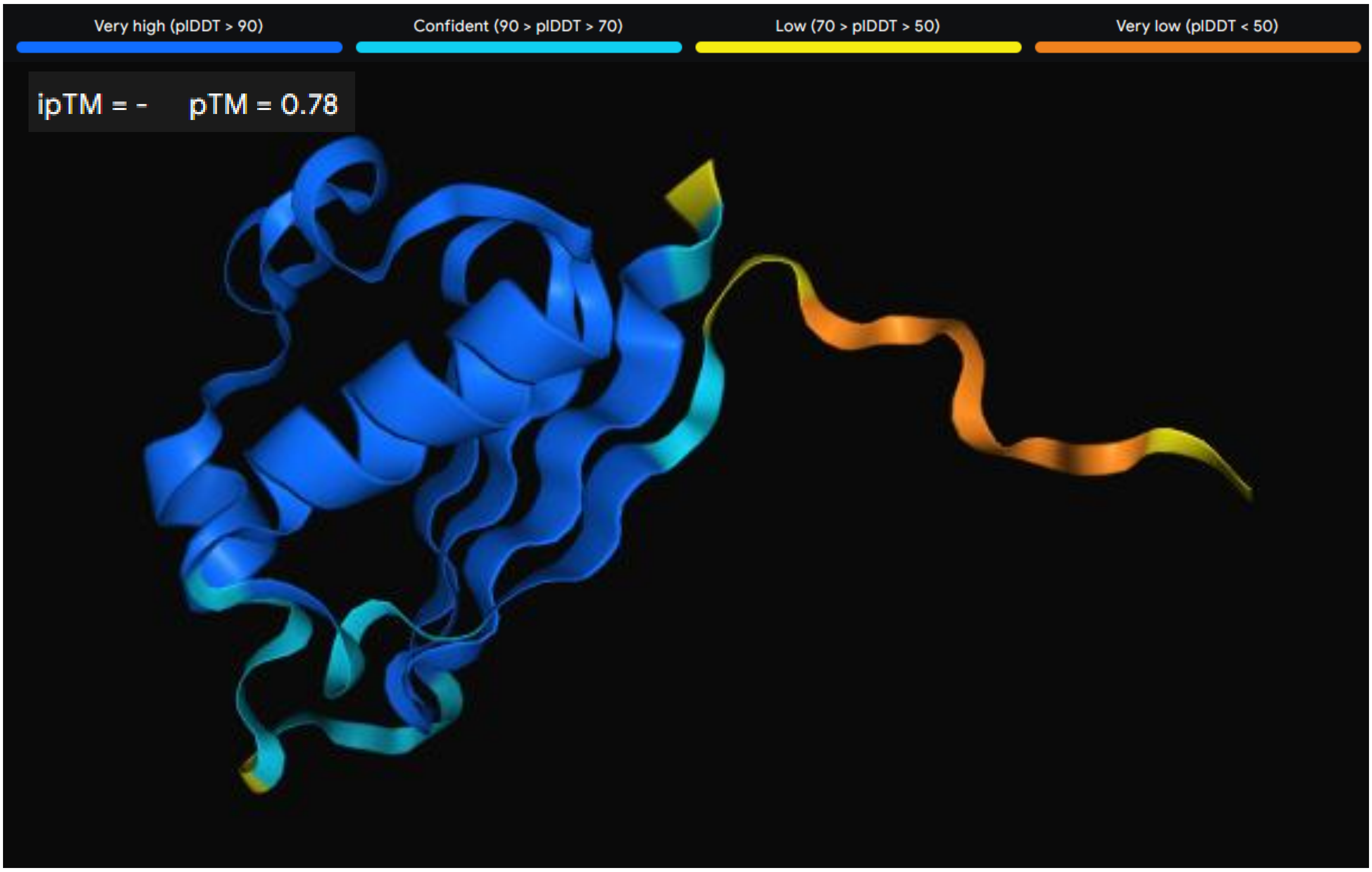

HVO_1561
conserved hypothetical protein

A

FUNCTION

Function Class:	CHY	(conserved hypothetical protein)
Superclass:	UNASS	(unassigned)

ORF PROPERTIES

Length(nucleic bases):	174	Start Codon:	ATG	Stop Codon:	TAA
%GC:	68%	CAI:	0.64		

PROTEIN PROPERTIES

Length(amino acids):	57	MolWeight(Da):	6,215
pI value:	11.3	GRAVY index:	0.54
TM domains:	1	signal sequence cleavage site:	No
TAT export signal prediction:	No	RR..LAGC motif:	No

B

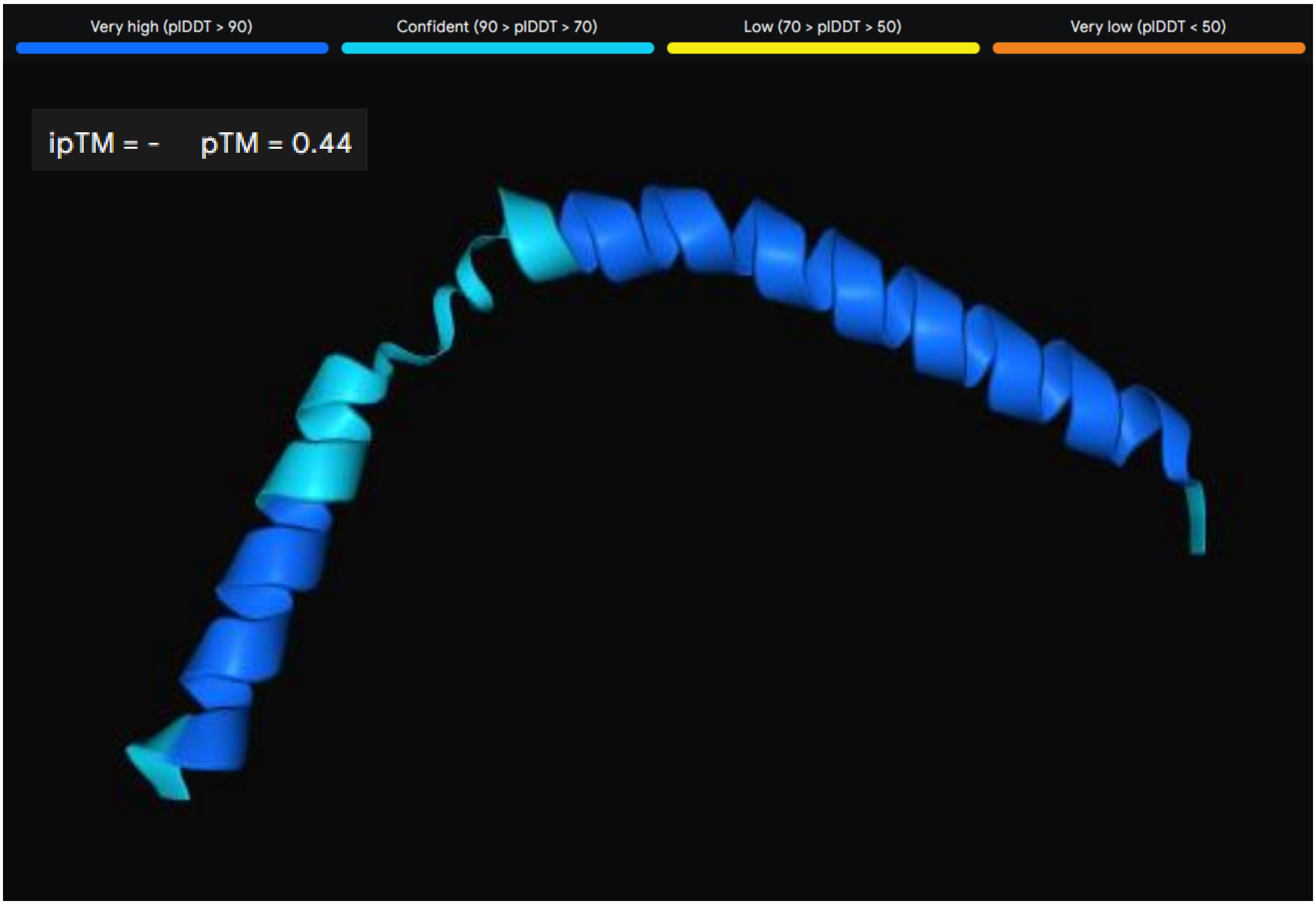

HVO_1556
conserved hypothetical protein

A

FUNCTION

Function Class:	CHY	(conserved hypothetical protein)
Superclass:	UNASS	(unassigned)

ORF PROPERTIES

Length(nucleic bases):	237	Start Codon:	ATG	Stop Codon:	TAA
%GC:	70%	CAI:	0.8		

PROTEIN PROPERTIES

Length(amino acids):	78	MolWeight(Da):	8,010
pI value:	4.8	GRAVY index:	0.75
TM domains:	2	signal sequence cleavage site:	No
TAT export signal prediction:	No	RR..LAGC motif:	No

B

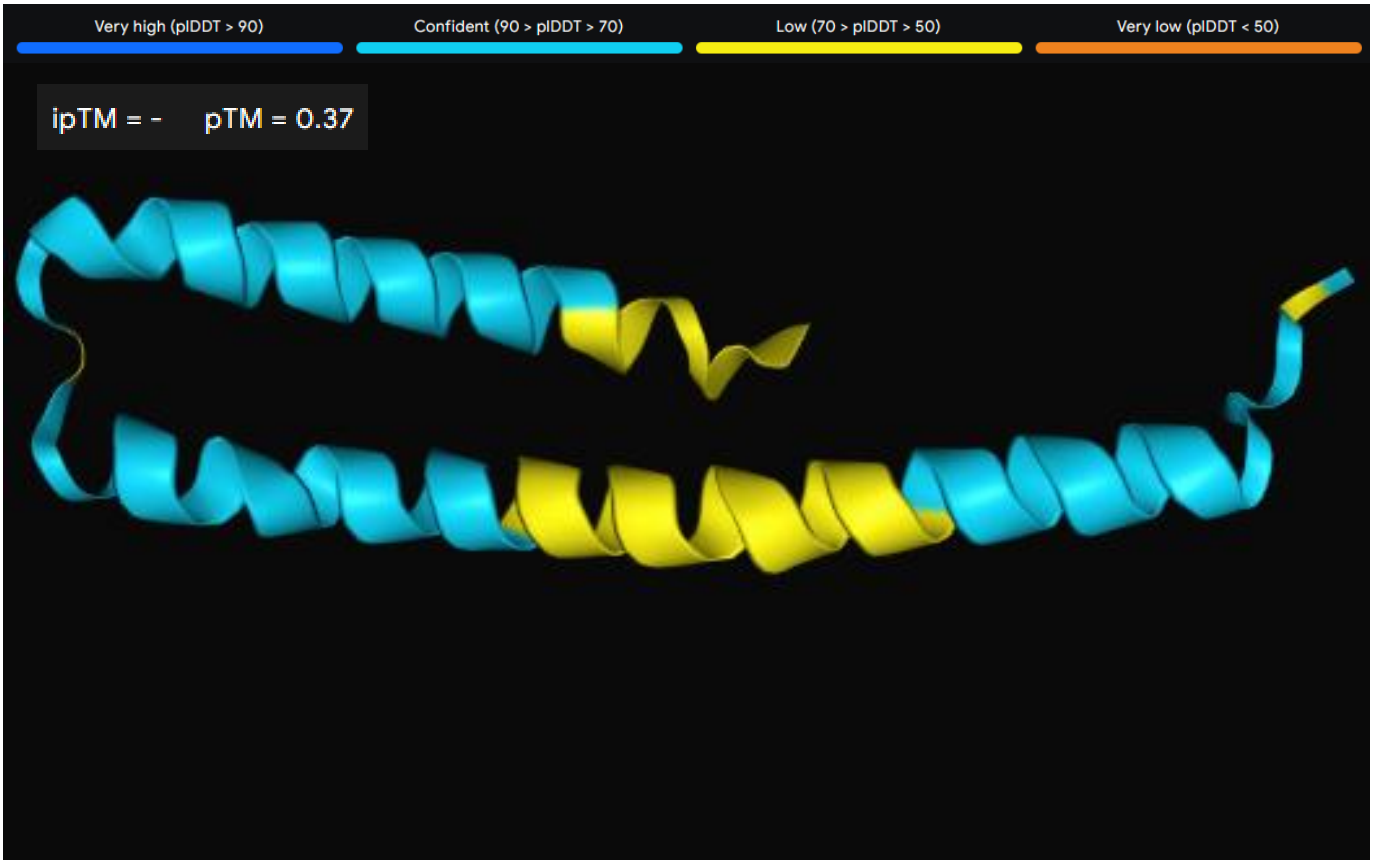

HVO_1863

conserved hypothetical protein

A

FUNCTION

Function Class:	CHY	(conserved hypothetical protein)
Superclass:	UNASS	(unassigned)

ORF PROPERTIES

Length(nucleic bases):	246	Start Codon:	ATG	Stop Codon:	TGA
%GC:	61%	CAI:	0.7		

PROTEIN PROPERTIES

Length(amino acids):	81	MolWeight(Da):	9,089
pI value:	5	GRAVY index:	-0.56
TM domains:	0	signal sequence cleavage site:	No
TAT export signal prediction:	No	RR..LAGC motif:	No

B

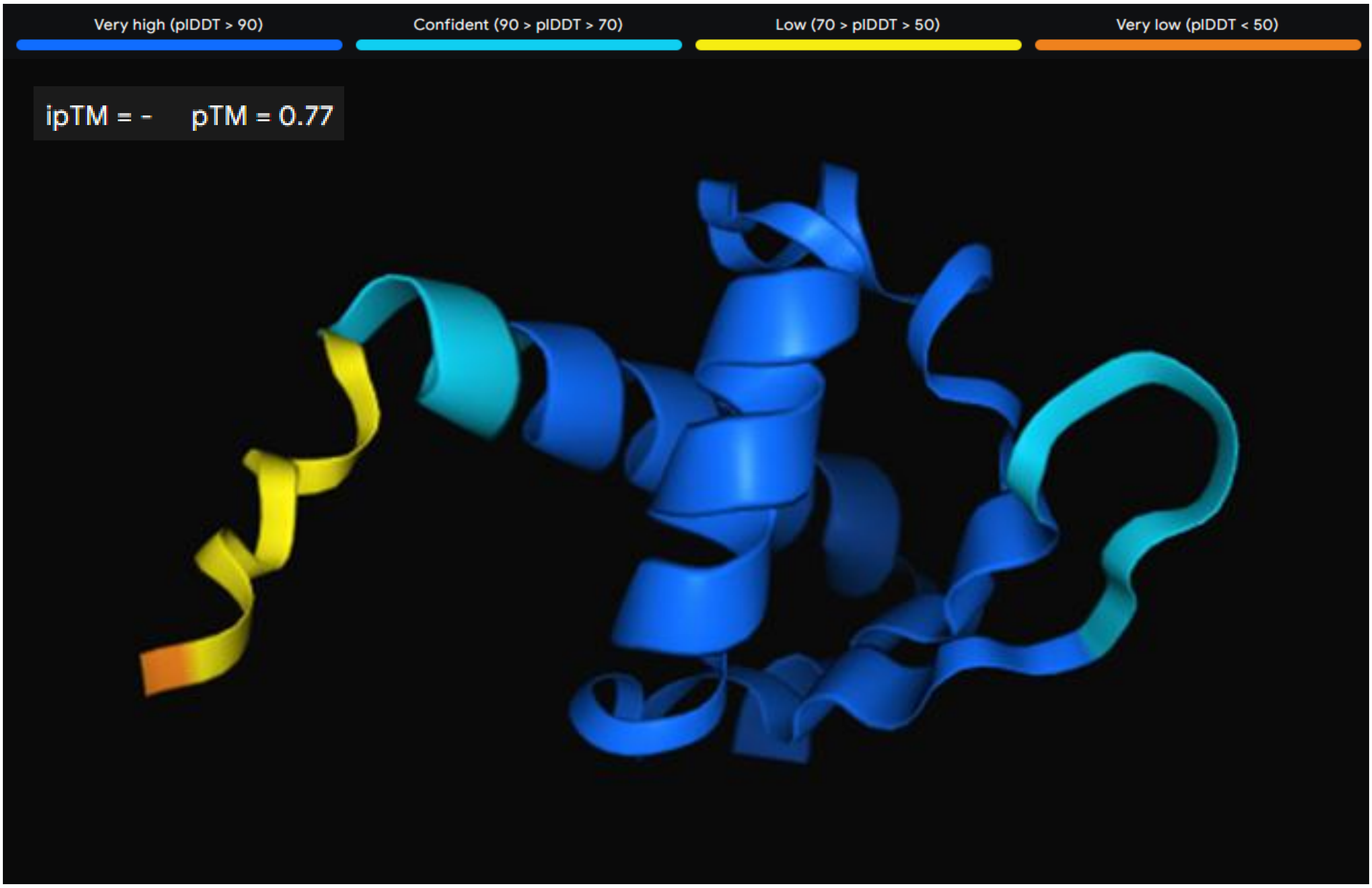

HVO_2447A
conserved hypothetical protein

A

FUNCTION

Function Class:	CHY	(conserved hypothetical protein)
Superclass:	UNASS	(unassigned)

ORF PROPERTIES

Length(nucleic bases):	267	Start Codon:	ATG	Stop Codon:	TAG
%GC:	65%	CAI:	0.57		

PROTEIN PROPERTIES

Length(amino acids):	88	MolWeight(Da):	9,565
pI value:	6.6	GRAVY index:	0.51
TM domains:	2	signal sequence cleavage site:	No
TAT export signal prediction:	No	RR..LAGC motif:	No

B

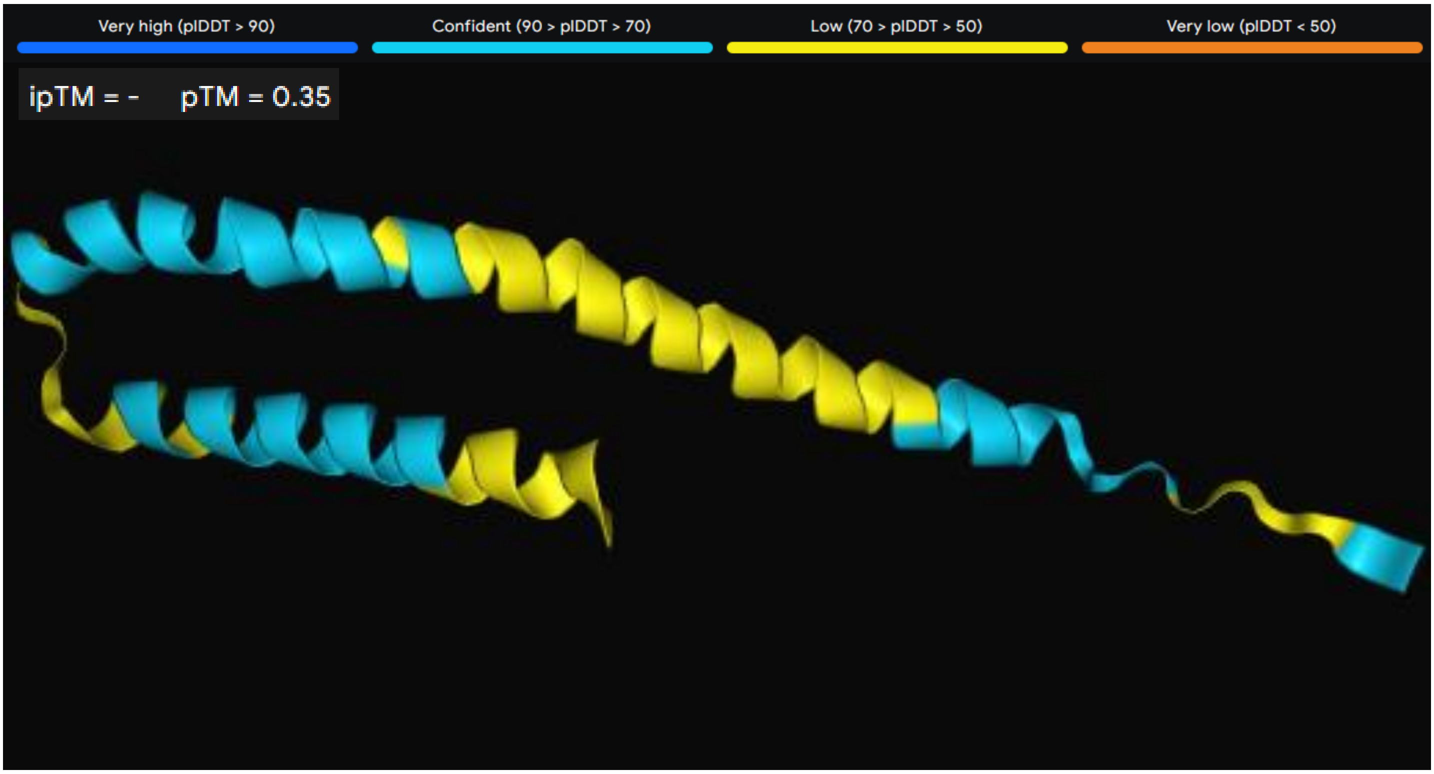

HVO_2583A

conserved hypothetical protein

A

FUNCTION

Function Class:	CHY	(conserved hypothetical protein)
Superclass:	UNASS	(unassigned)

ORF PROPERTIES

Length(nucleic bases):	201	Start Codon:	ATG	Stop Codon:	TGA
%GC:	72%	CAI:	0.6		

PROTEIN PROPERTIES

Length(amino acids):	66	MolWeight(Da):	6,993
pI value:	10.2	GRAVY index:	-0.02
TM domains:	1	signal sequence cleavage site:	No
TAT export signal prediction:	No	RR..LAGC motif:	No

B

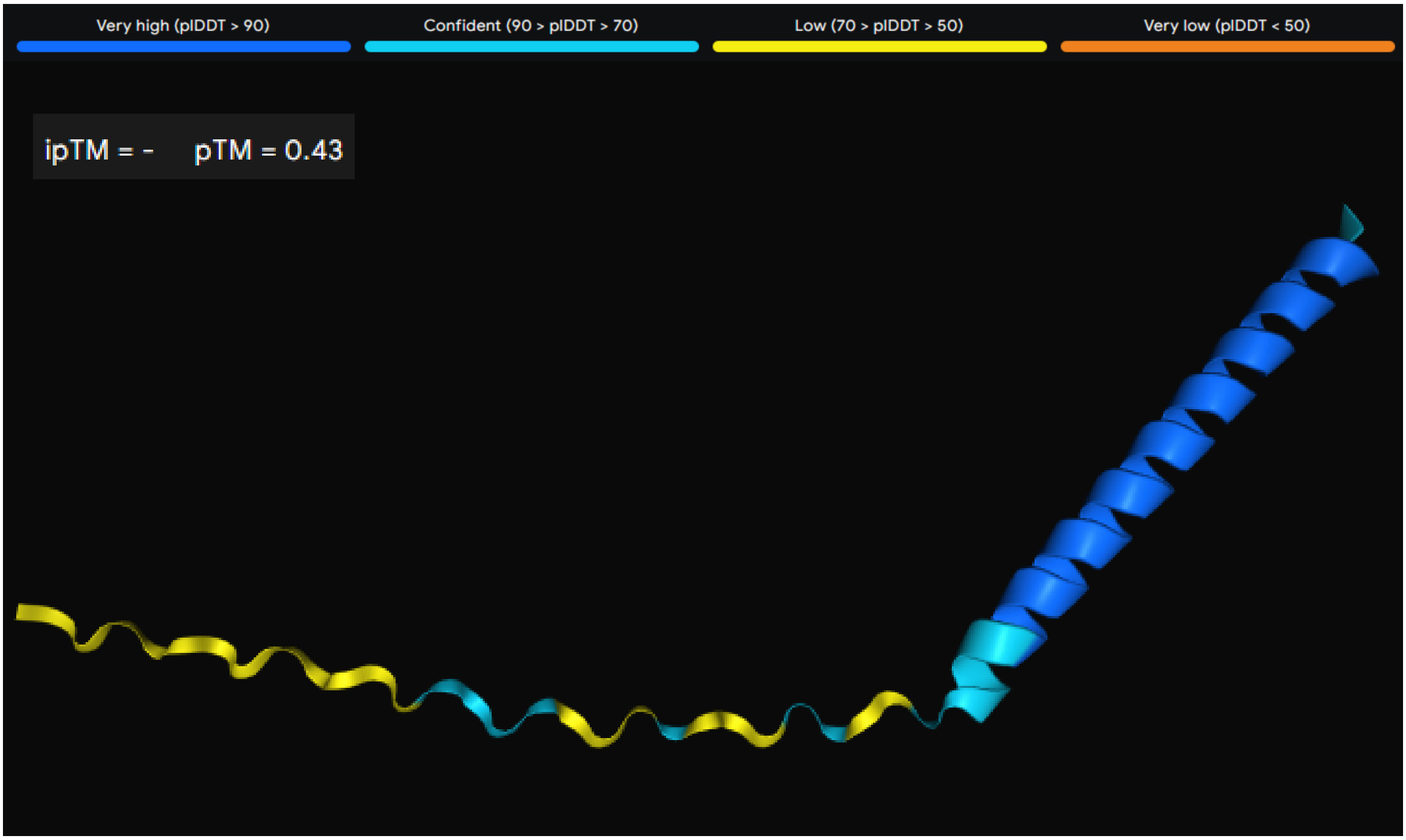

HVO_2955A

conserved hypothetical protein

A

FUNCTION

Function Class:	CHY	(conserved hypothetical protein)
Superclass:	UNASS	(unassigned)

ORF PROPERTIES

Length(nucleic bases):	108	Start Codon:	ATG	Stop Codon:	TAA
%GC:	63%	CAI:			

PROTEIN PROPERTIES

Length(amino acids):	35	MolWeight(Da):	3,885
pI value:	10	GRAVY index:	-0.73
TM domains:	not available	signal sequence cleavage site:	not available
TAT export signal prediction:	not available	RR..LAGC motif:	not available

B

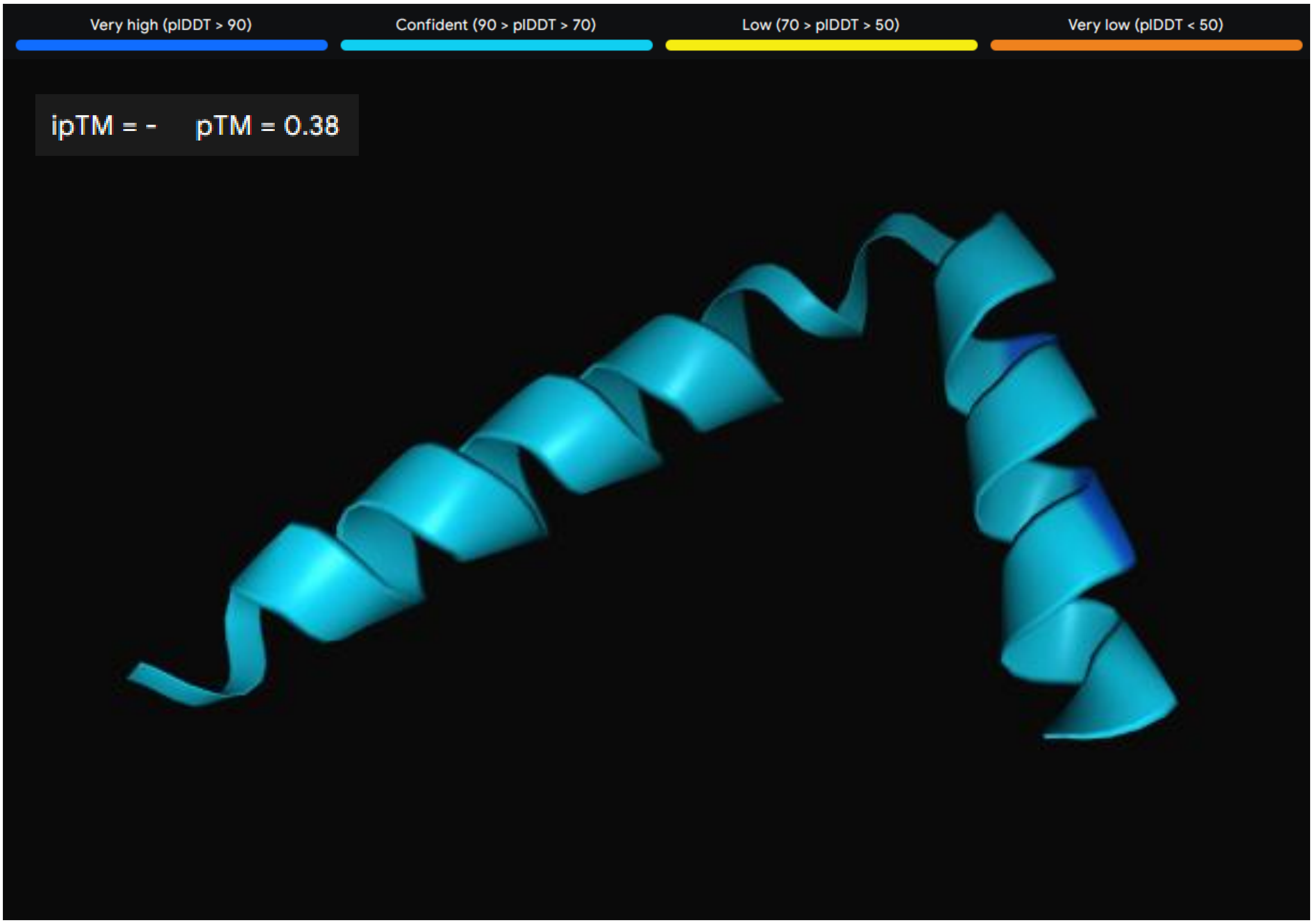

HVO_2983_A

conserved hypothetical protein

A

FUNCTION

Function Class:	CHY	(conserved hypothetical protein)
Superclass:	UNASS	(unassigned)

ORF PROPERTIES

Length(nucleic bases):	117	Start Codon:	ATG	Stop Codon:	TAG
%GC:	62%	CAI:	0.73		

PROTEIN PROPERTIES

Length(amino acids):	38	MolWeight(Da):	4,043
pI value:	5.4	GRAVY index:	0.15
TM domains:	0	signal sequence cleavage site:	No
TAT export signal prediction:	No	RR..LAGC motif:	No

B

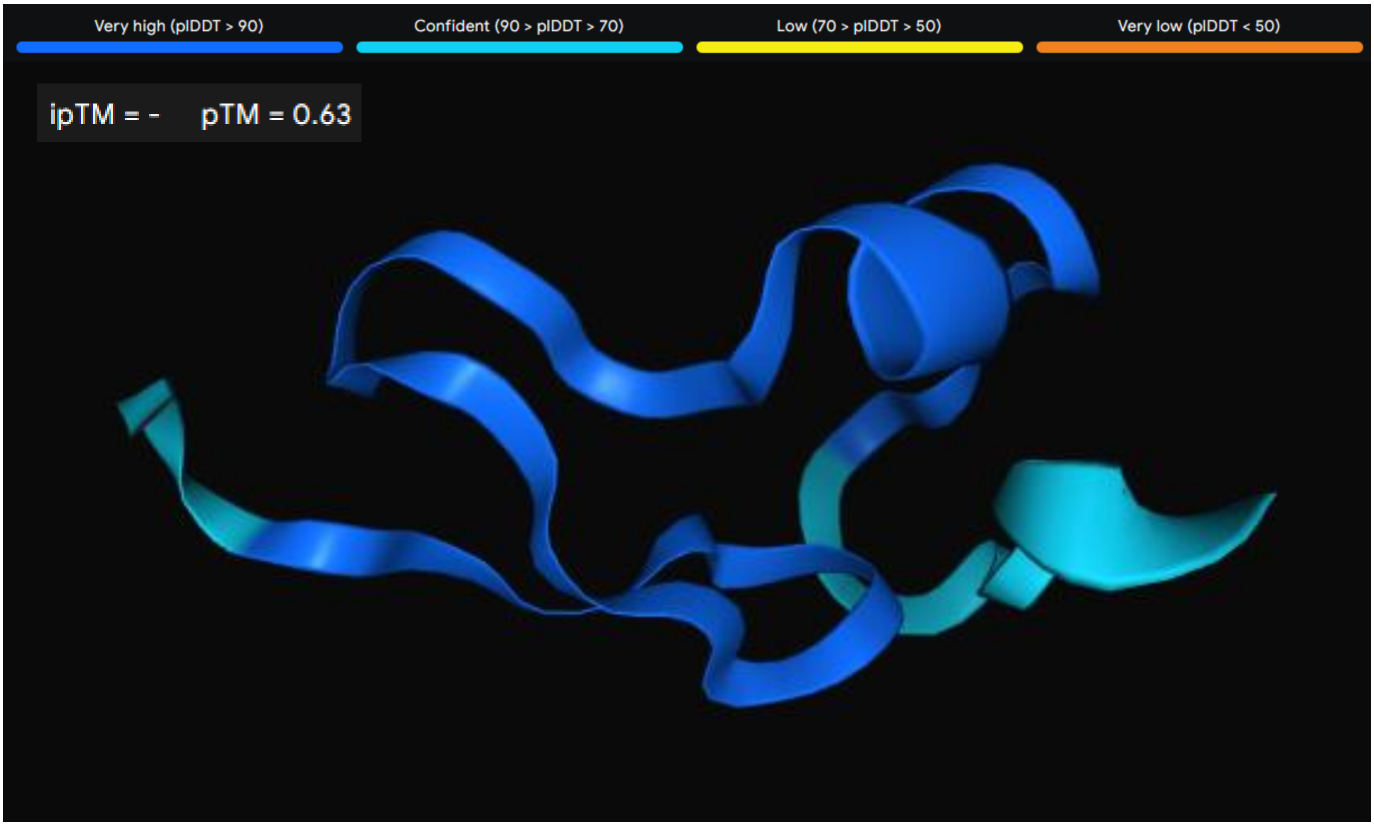

HVO_B0276

DMT superfamily transport protein

A

FUNCTION

Function Class:	TP	(small molecule transport)
Superclass:	TP_CP	(transport and cellular processes (CE, CP, MOT, SEC, TP))

ORF PROPERTIES

Length(nucleic bases):	972	Start Codon:	GTG	Stop Codon:	TGA
%GC:	71%	CAI:	0.76		

PROTEIN PROPERTIES

Length(amino acids):	323	MolWeight(Da):	33,199
pI value:	6.7	GRAVY index:	0.97
TM domains:	9	signal sequence cleavage site:	Yes
TAT export signal prediction:	No	RR..LAGC motif:	No

B

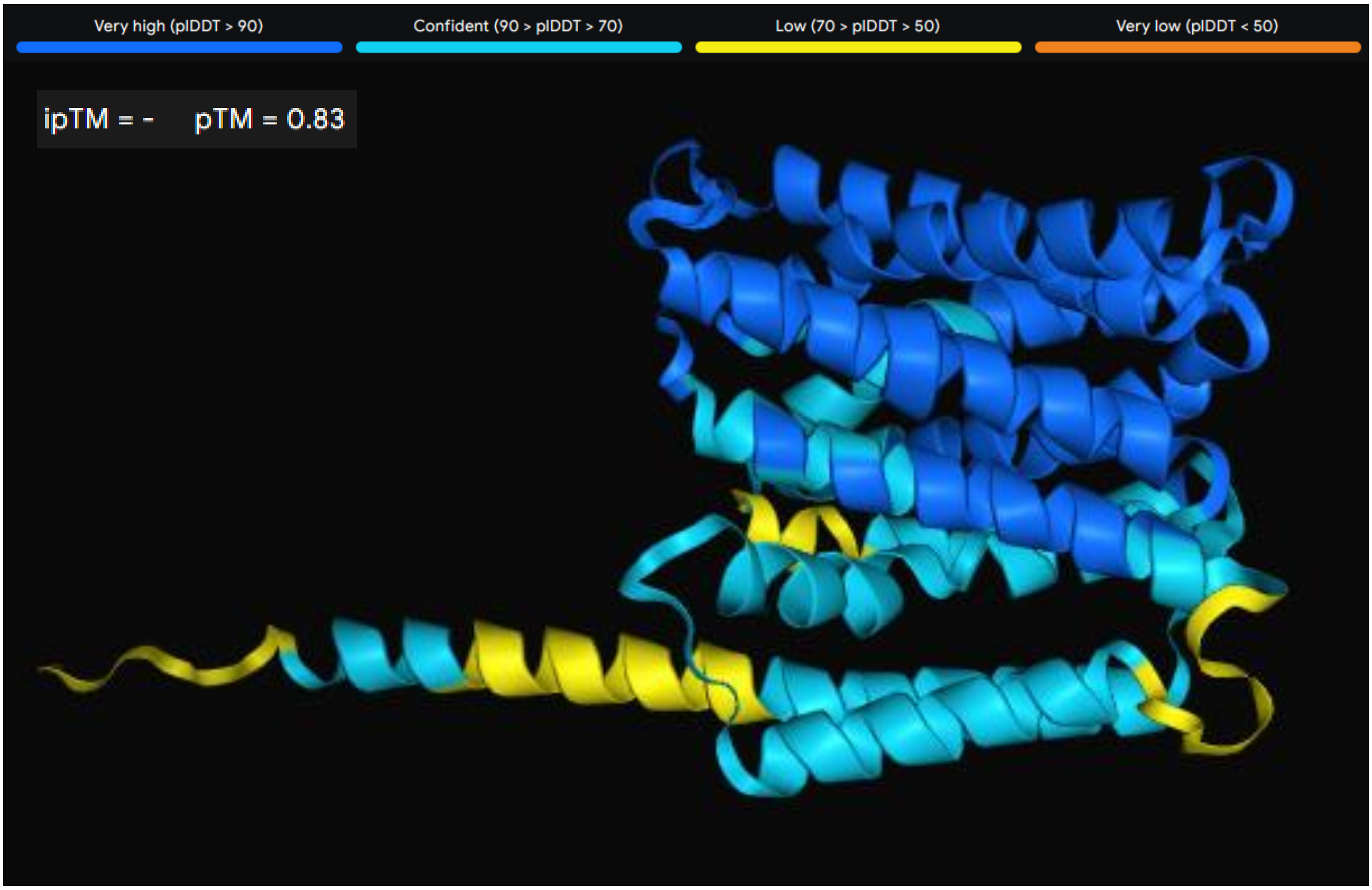

A

HVO_2579 - nicotinate-nucleotide pyrophosphorylase (carboxylating)

FUNCTION

Function Class:	COM	(coenzyme metabolism)
Superclass:	MET	(metabolism (AA, CHM, CIM, COM, EM, LIP, NUM))

ORF PROPERTIES

Length(nucleic bases):	807	Start Codon:	GTG	Stop Codon:	TGA
%GC:	72%	CAI:	0.78		

PROTEIN PROPERTIES

Length(amino acids):	268	MolWeight(Da):	27,929
pI value:	4.8	GRAVY index:	-0.07
TM domains:	0	signal sequence cleavage site:	No
TAT export signal prediction:	No	RR..LAGC motif:	No

HVO_2580 - L-aspartate oxidase

FUNCTION

Function Class:	COM	(coenzyme metabolism)
Superclass:	MET	(metabolism (AA, CHM, CIM, COM, EM, LIP, NUM))

ORF PROPERTIES

Length(nucleic bases):	1614	Start Codon:	ATG	Stop Codon:	TGA
%GC:	75%	CAI:	0.79		

PROTEIN PROPERTIES

Length(amino acids):	537	MolWeight(Da):	55,753
pI value:	4.4	GRAVY index:	-0.21
TM domains:	0	signal sequence cleavage site:	Yes
TAT export signal prediction:	No	RR..LAGC motif:	No

HVO_2581 - quinolinate synthase A

FUNCTION

Function Class:	COM	(coenzyme metabolism)
Superclass:	MET	(metabolism (AA, CHM, CIM, COM, EM, LIP, NUM))

ORF PROPERTIES

Length(nucleic bases):	1158	Start Codon:	GTG	Stop Codon:	TGA
%GC:	69%	CAI:	0.78		

PROTEIN PROPERTIES

Length(amino acids):	385	MolWeight(Da):	41,990
pI value:	4.3	GRAVY index:	-0.17
TM domains:	0	signal sequence cleavage site:	No
TAT export signal prediction:	No	RR..LAGC motif:	No

B

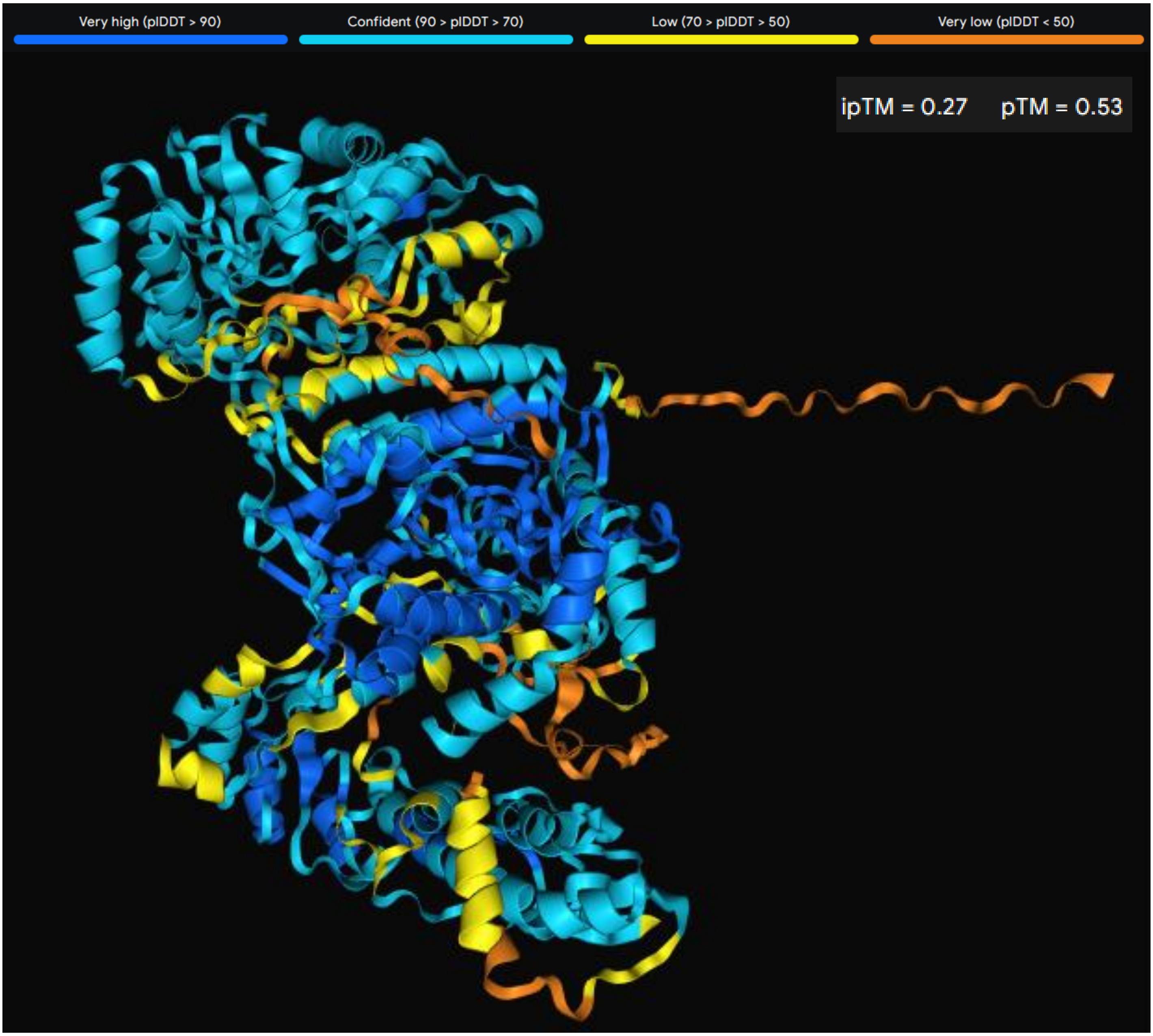
Very high (plDDT > 90)
Confident (90 > plDDT > 70)
Low (70 > plDDT > 50)
Very low (plDDT < 50)
ipTM = 0.27 pTM = 0.53

Supplementary Figure S8: Predicted alpha fold 3 structures of upregulated genes and knock out candidates. A) Gene and protein infos of the respective gene obtained from the halolex data base. B) predicted protein structure using the AlphaFold Server/AlphaFold 3 (https://alphafoldserver.com). In case of HVO_2579, HVO_2580 and HVO_2581, the structure of a heterotrimeric complex of these three proteins is predicted. Confidence metrics pLDDT and pTM/ipTM scores are also shown.
