# Supplementary material for: Studying the long-term adaptation of Haloferax volcanii to low salt conditions: transcriptomic and genetic analyses
Source: Front Microbiol. 2026 Jan 15;16:1697018. doi: 10.3389/fmicb.2025.1697018 (PMC12852389; doi:10.3389/fmicb.2025.1697018)
Supplement: Supplementary file 9 [file Data_Sheet_9.pdf]

1 10 20 30 40 50 60 70 80 90

HVO_0772 MSTTTAEPDT EDLLSEAEFR ERLRELPPSA KLVAKVLESD APLSQGQLAE ESLLPDRTVR YALNRLEESD LVGSRYSFKD ARKQVYFLNT

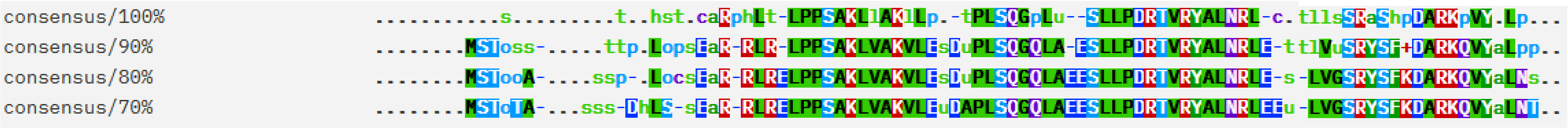

1 10 20 30 40 50 60 70 80

HVO_1863 MSATVPPSTD DSSKEDRLKQ YLLDRAKDGE MYFKSKFIAD DVGLSPKEIG ALMVKLRDSA TDLSIEKWSY TSATTWRVET A

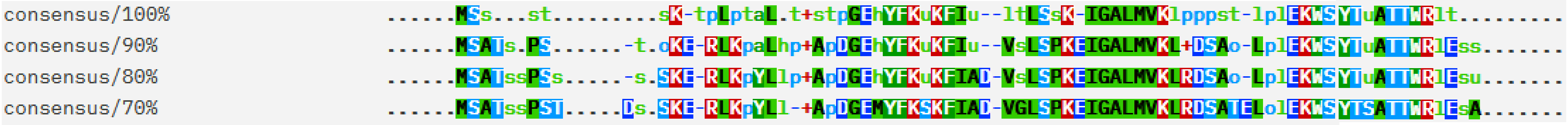

	1	10	20	30	40	50	60	70	80

HVO_B0276

MTATRRRLVL FVLTAFFFGG TFVAAKAGLD YLPPLLFVAL RFDIAAVLLV GYVVATRSRA ELLPRSVRDV VGILATGVFV
IGLANALLFV GQEHVSSGVG SIIFSLNPIL TPVFAMALLA DERLSTRGVL GMLIGLLGVGLVVGVDPANL LDGEALWKGV
VFLGAVSGAL GTVLIRWADT SLSSTVRTAW ALPVSAALTH GMSVASGESL AAATWSPTAL VALAYVGVFA GAVAYLTYFG
FLDDVGPIRG NLVFYAVPIV ATLGGTALLG ESISTLTVVG FATIFTGFAV LATESLRGTV VRVYDELGSR VAGLAGGAAP
GRD

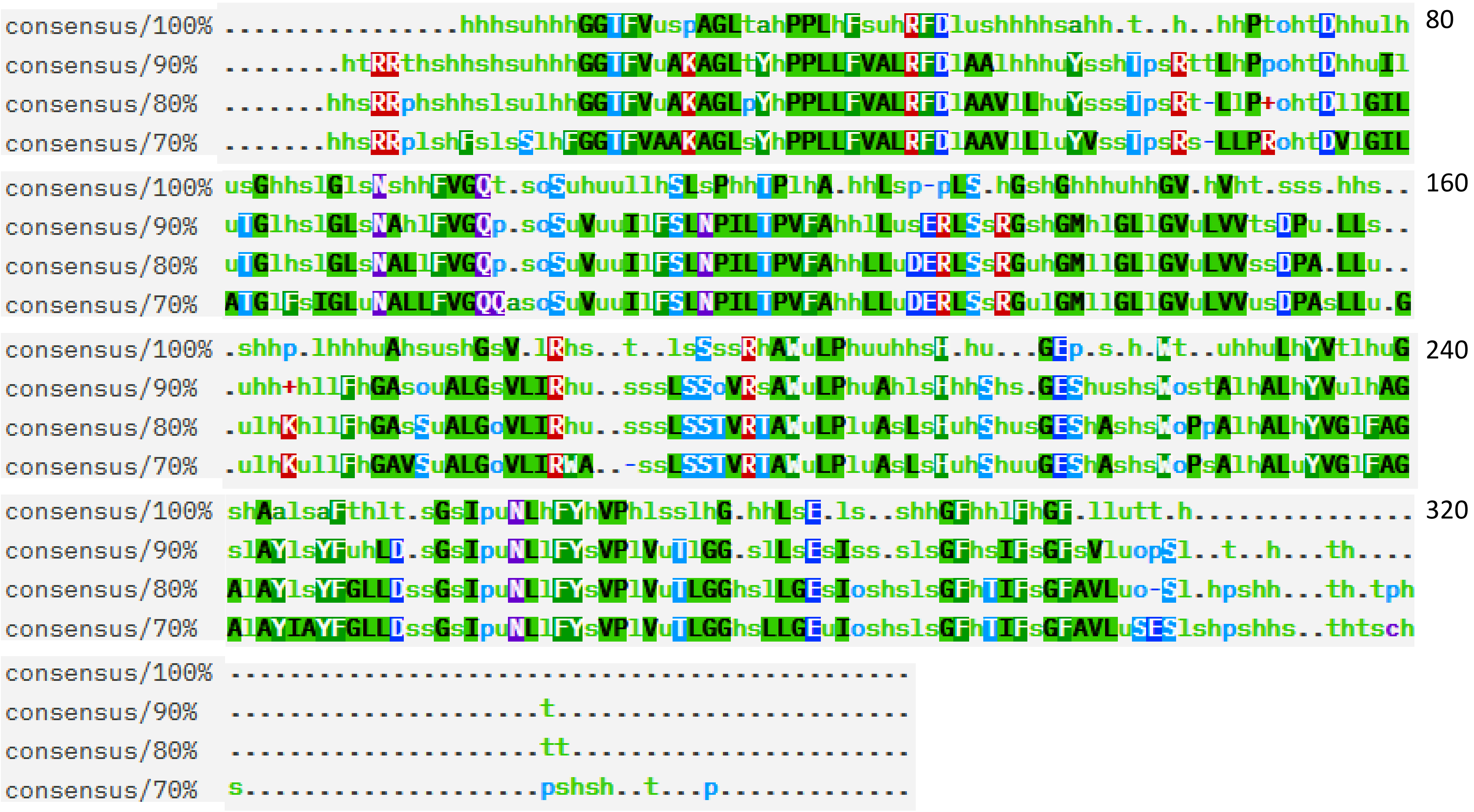

Supplementary Figure S9: Degree of conservation via multiple sequence alignment (MSA) of the proteins HVO_0772, HVO_1863 and HVO_B0276. A MSA was generated (using the ClustalOmega program and MView tool at the EMBL-EBI site) of the indicated protein and the top 99 hits of a protein blast search at NCBI. At each position the residues are shown that are, respectively, 100%, 90%, 80%, or 70% conserved. Lower-case letter legend: a → aromatic, h → hydrophobic, l → aliphatic, o → alcohol, p → polar, s → small (A, C, D, G, N, P, S, T, V), t → turnlike, u → tiny (A, G, S). The color identity palette is shown below and was taken from (https://desmid.github.io/mview/manual/manual.html#colouring-modes).

```
[P1]
#protein: highlight amino acid physicochemical properties
*         ->  dark-gray           #mismatch
?         ->  light-gray          #unknown
Aa        =>  bright-green        #hydrophobic
Bb        =>  dark-gray           #D or N
Cc        =>  yellow              #cysteine
Dd        =>  bright-blue         #negative charge
Ee        =>  bright-blue         #negative charge
Ff        =>  dark-green          #large hydrophobic
Gg        =>  bright-green        #hydrophobic
Hh        =>  dark-green          #large hydrophobic
Ii        =>  bright-green        #hydrophobic
Kk        =>  bright-red          #positive charge
Ll        =>  bright-green        #hydrophobic
Mm        =>  bright-green        #hydrophobic
Nn        =>  purple              #polar
Pp        =>  bright-green        #hydrophobic
Qq        =>  purple              #polar
Rr        =>  bright-red          #positive charge
Ss        =>  dull-blue           #small alcohol
Tt        =>  dull-blue           #small alcohol
Vv        =>  bright-green        #hydrophobic
Ww        =>  dark-green          #large hydrophobic
Xx        ->  dark-gray           #any
Yy        =>  dark-green          #large hydrophobic
Zz        =>  dark-gray           #E or Q
```
