# Supplementary material for: Studying the long-term adaptation of Haloferax volcanii to low salt conditions: transcriptomic and genetic analyses
Source: Front Microbiol. 2026 Jan 15;16:1697018. doi: 10.3389/fmicb.2025.1697018 (PMC12852389; doi:10.3389/fmicb.2025.1697018)
Supplement: Supplementary file 14 [file Table_5.docx]

**Supplementary Table S1**

| **Gene Name** | **Primer Name** | **Sequence** |
| --- | --- | --- |
| **HVO_0772** | P1 0772 | CGATGAGCCGACCGCCCTTG |
|  | P2 0772 | GACCTGCTTTCGCGCTGCGGTGGTGGTGCTCATAC |
|  | P3 0772 | AGCACCACCACCGCAGCGCGAAAGCAGGTCTACTTCCTC |
|  | P4 0772 | CGTGTCGAGCACCTCGACGC |
| **HVO_1556** | P1 1556 | CGGCCTCGCTCGCTTCGATG |
|  | P2 1556 | GCGCCCGAGGACGAGGTCGAGCGCGATCGAGTCGTTC |
|  | P3 1556 | TCGATCGCGCTCGACCTCGTCCTCGGGCGCGACCAC |
|  | P4 1556 | ACACGGCCTCATCGCCCTCG |
| **HVO_1863** | P1 1863 | TCCGCGTCCTCATGCACCAC |
|  | P2 1863 | GTGTCTCAGGCGGTCTCGTCGGTGGAGGGGGGTACTG |
|  | P3 1863 | CCCCTCCACCGACGAGACCGCCTGAGACACGCACTG |
|  | P4 1863 | GGCGCGAAGTGCCTCCACCG |
| **HVO_2983_A** | P1 2983_A | CCGTCAACGACCAGGGACGG |
|  | P2 2983_A | GGAATCGACGCGCTAACAGCACATGCAACTGCGCATGG |
|  | P3 2983_A | AGTTGCATGTGCTGTTAGCGCGTCGATTCCGGTTCGAATCC |
|  | P4 2983_A | TGCCGCCGTAGTAGCGCGTC |
| **HVO_2583A** | P1 2583A | CCAACGTCGTCGCCGCGATG |
|  | P2 2583A | AGCTTCGCCACGGTCCTCCCCGACGGACGCATTTTCATC |
|  | P3 2583A | GCGTCCGTCGGGGAGGACCGTGGCGAAGCTCCTCGGC |
|  | P4 2583A | GGCAGGCGACACCAGACGAAG |
| **HVO_2955A** | P1 2955A | GAACACCGGTCGCCCATGAG |
|  | P2 2955A | CGCAGGCCCGCTTACCGGCGATGTTTCCAGTTCATACTC |
|  | P3 2955A | CTGGAAACATCGCCGGTAAGCGGGCCTGCGCCTCG |
|  | P4 2955A | GCTCGCCGGTGACGACGTAGAC |
| **HVO_0777** | P1 0777 | CGGGTGCTCTACGGCGTCAG |
|  | P2 0777 | GACGGTGAGCGTCCCGGACCCAACTCCATCTTCGAACGG |
|  | P3 0777 | GATGGAGTTGGGTCCGGGACGCTCACCGTCCACCC |
|  | P4 0777 | GCGAGGAGTTCGCCGGTGAG |
| **HVO_2447A** | P1 2447A | TCGAGTCCGCCTTCGGCCTG |
|  | P2 2447A | CGTAGTTGTGGCCGAGGAGTTGGGAGAGGCGTTCACGG |
|  | P3 2447A | GCCTCTCCCAACTCCTCGGCCACAACTACGCCCGTTC |
|  | P4 2447A | TTGGCGTCCTCGCGGGTGGG |
| **HVO_1561** | P1 1561 | CTCGACGTACTCCTTGTCCTTCTG |
|  | P2 1561 | GCGGCGCGTTCGTAGGCGAACAGGACGTACTTGGTTAGG |
|  | P3 1561 | GTACGTCCTGTTCGCCTACGAACGCGCCGCGGGTC |
|  | P4 1561 | GGAGGGAGCTGATGAGCGACTG |
| **HVO_0665** | P1 0665 | CGACTGCGCGACCGACGAAG |
|  | P2 0665 | TGCTCACGTCGGGCGCTTCCGTCGCGTCGGTGAAGC |
|  | P3 0665 | CCGACGCGACGGAAGCGCCCGACGTGAGCATCTCC |
|  | P4 0665 | GAGTCTCCTGTGGGCGCTCGG |
| **HVO_B0276** | P1 B0276 | TTTGGCGTCGGTGTCGAGGATG |
|  | P2 B0276 | GCGGGACCCGAGTTCGAACGCCGTCAGCACGAACAGG |
|  | P3 B0276 | GTGCTGACGGCGTTCGAACTCGGGTCCCGCGTGGC |
|  | P4 B0276 | TCCTGCGTCTGGCCGAAATC |
| **HVO_2579-2581** | P1 2579-2581 | CGCCGAGGTCGAATCCGACG |
|  | P2 2579-2581 | TCGGAACTCGGCGCGTCGCGGTGGCTCTCCGGTAG |
|  | P3 2579-2581 | GGAGAGCCACCGCGACGCGCCGAGTTCCGACTTCTCC |
|  | P4 2579-2581 | GCGGCGAACTCGTGGGTCTC |

Oligonucleotides (P1-P4) for the generation of in-frame deletion mutants:

Oligonucleotides for checking deletion:

| **Gene Name** | **Primer Name** | **Sequence** |
| --- | --- | --- |
| **HVO_0665** | Ana d0665 fw | TCGTCGCGGCCAAGGAACTC |
|  | Ana d0665 rv | GCGCTCGGAGAGCTTCGAGAGG |
| **HVO_2579-2581** | Ana 2579-81 fw | CATGAACTCCTACGCCGACCTG |
|  | Ana 2579-81 rv | GAGCACCCACGTCAGGTAGTTG |

Oligonucleotides for the generation of pSZ-based expression vectors:

| **Gene Name** | **Primer Name** | **Sequence** |
| --- | --- | --- |
| **HVO_0772** | HVO_0772 for His | GACTAGCATATGCACCACCACCACCACCACAGCACCACCACCGCAGAGCCAGATAC |
|  | HVO_0772 for | GACTAGCATATGAGCACCACCACCGCAGAGCCAGATAC |
|  | HVO_0772 rev His | GACTAGGGTACCTCAGTGGTGGTGGTGGTGGTGCGTGTTGAGGAAGTAGACCTGCTTTCG |
|  | HVO_0772 rev | GACTAGGGTACCTCACGTGTTGAGGAAGTAGACCTGCTTTCG |
| **HVO_1863** | HVO_1863 for His | GACTAGCATATGCACCACCACCACCACCACAGCGCAACAGTACCCCCCTCCACC |
|  | HVO_1863 for | GACTAGCATATGAGCGCAACAGTACCCCCCTCCACC |
|  | HVO_1863 rev His | GACTAGGGTACCTTAGTGGTGGTGGTGGTGGTGGGCGGTCTCGACGCGCCACG |
|  | HVO_1863 rev | GACTAGGGTACCTTAGGCGGTCTCGACGCGCCACG |
| **HVO_B0276** | HVO_B0276 for His | GACTAGCATATGCACCACCACCACCACCACACGGCGACGCGCCGGCGGCTCGTC |
|  | HVO_B0276 for | GACTAGCATATGACGGCGACGCGCCGGCGGCTCGTC |
|  | HVO_B0276 rev His | GACTAGGGTACCTTAGTGGTGGTGGTGGTGGTGGTCGCGTCCCGGAGCCGCGCCC |
|  | HVO_B0276 rev | GACTAGGGTACCTTAGTCGCGTCCCGGAGCCGCGCCC |

Oligonucleotides for the generation of Digoxigenin-labeled probes:

| **Gene Name** | **Primer Name** | **Sequence** | **Probe lenght (nt)** |
| --- | --- | --- | --- |
| **HVO_0772** | 0772 Probe fw | CCCTTAACACGGAGCAACCGACTG | 382 |
|  | 0772 Probe rv | AGAGAAGTGGTGGCTTGGTCTG |  |
| **HVO_1863** | 1863 Probe fw | TTCAGGCTGAAAGCGGCCGAAC | 491 |
|  | 1863 Probe rv | AGTCGCGTCTCGGTTCGGTTTC |  |
| **HVO_B0276** | B0276 Probe fw | GTTCGTCATCGGCCTCGCCAAC | 457 |
|  | B0276 Probe rv | GGCGAAGACGCCGACGTAGG |  |
